# The impact of life stage and pigment source on the evolution of novel warning signal traits

**DOI:** 10.1111/evo.14443

**Published:** 2022-02-10

**Authors:** Carita Lindstedt, Robin K. Bagley, Sara Calhim, Mackenzie Jones, Catherine R. Linnen

**Affiliations:** ^1^ Department of Biological and Environmental Sciences University of Jyväskylä Jyväskylä 40014 Finland; ^2^ Department of Forest Science, P.O. Box 27, 00014 University of Helsinki Finland; ^3^ Department of Biology University of Kentucky Lexington Kentucky 40506; ^4^ Department of Evolution, Ecology, and Organismal Biology The Ohio State University at Lima Lima Ohio 45804

**Keywords:** Aposematism, carotenoids, chemical defense, ecological genetics, host adaptation, polytypic coloration

## Abstract

Our understanding of how novel warning color traits evolve in natural populations is largely based on studies of reproductive stages and organisms with endogenously produced pigmentation. In these systems, genetic drift is often required for novel alleles to overcome strong purifying selection stemming from frequency‐dependent predation and positive assortative mating. Here, we integrate data from field surveys, predation experiments, population genomics, and phenotypic correlations to explain the origin and maintenance of geographic variation in a diet‐based larval pigmentation trait in the redheaded pine sawfly (*Neodiprion lecontei*), a pine‐feeding hymenopteran. Although our experiments confirm that *N. lecontei* larvae are indeed aposematic—and therefore likely to experience frequency‐dependent predation—our genomic data do not support a historical demographic scenario that would have facilitated the spread of an initially deleterious allele via drift. Additionally, significantly elevated differentiation at a known color locus suggests that geographic variation in larval color is currently maintained by selection. Together, these data suggest that the novel white morph likely spread via selection. However, white body color does not enhance aposematic displays, nor is it correlated with enhanced chemical defense or immune function. Instead, the derived white‐bodied morph is disproportionately abundant on a pine species with a reduced carotenoid content relative to other pine hosts, suggesting that bottom‐up selection via host plants may have driven divergence among populations. Overall, our results suggest that life stage and pigment source can have a substantial impact on the evolution of novel warning signals, highlighting the need to investigate diverse aposematic taxa to develop a comprehensive understanding of color variation in nature.

Determining how novel traits originate and become abundant is a core goal of evolutionary biology. To this end, vibrant colors that animals use to communicate their unprofitability to predators have made aposematic species attractive model systems (Cuthill et al. 2017; Briolat et al. 2018). Aposematic colors, such as orange, yellow, red or white combined with black pattern elements, can make naïve predators reluctant to attack prey due to innate biases such as neophobia (Mappes and Alatalo 1997; Thomas et al. 2004). Furthermore, predators learn to associate warning color pattern with the unprofitability of the prey and avoid attacking prey individuals with a similar appearance in future encounters (Ruxton et al. 2004). As a result, the fitness of a particular aposematic color morph is expected to be dependent on its local abundance and evolve under positive frequency‐dependent selection (Rowland et al. 2007; Briolat et al. 2018). This frequency‐dependence is expected to reduce genetic and phenotypic variation in signal design because rare or novel color patterns can suffer increased predation risk (Kapan 2001; Arias et al. 2016; Chouteau et al. 2016). However, intraspecific variation in warning color is surprisingly common both within populations (polymorphism) and among populations (polytypism) of aposematic species (Briolat et al. 2018). Such variation offers valuable opportunities for characterizing the evolutionary and ecological processes that generate novel warning color traits and signal divergence (Dasmahapatra et al. 2012).

Three evolutionary scenarios could explain the origin and spread of novel warning color alleles that produce color differences among aposematic populations. First, under some circumstances, novel color alleles may evolve neutrally or under weak selection from predators. For example, when predators focus only on a specific element or combination of elements in an aposematic signal, variation in other signal elements may have little to no impact on predation rates (Winters et al. 2017; Rönkä et al. 2018a). There can also be variation in predator cognition and foraging behavior due to neophobia or dietary conservatism, which can result in weaker selection against novel signal forms by predators (e.g., Thomas et al. 2004; Aubier and Sherratt 2015), especially if the prey is aggregated (Mappes and Alatalo 1997; Mappes et al. 1999). Under this scenario, among‐population differentiation at color loci evolves under migration‐drift balance.

Second, even when novel color alleles evolve under negative (purifying) selection, stochastic shifts in color allele frequencies that occur when a small number of individuals colonize a new area or survive a population bottleneck could enable novel color alleles to reach threshold frequencies at which they are common enough to be protected (Mallet and Singer 1987; Turner et al. 1996; Mallet and Joron 1999; Mallet 2010). For both of the first two scenarios (neutrality and purifying selection), novel color alleles increase in frequency via genetic drift. The main difference between these scenarios is in the degree of isolation and drift needed to explain differentiation at color loci: the stronger the selection against a novel color allele, the more isolation and drift required to enable the allele to spread.

A third explanation for polytypic warning color is that novel warning color alleles are favored in some populations. Because a multitude of selection pressures can act on color alleles, the net selection coefficient for a novel color allele can be positive, even when the color morph it produces experiences an elevated predation risk. For example, warning signal efficacy may trade off with enhanced thermoregulation (Lindstedt et al. 2009; Hegna et al. 2013) or improved defense against pathogens (Friman et al. 2009). Less efficient warning signal forms can also be favored under certain dietary conditions (Talloen et al. 2004; Lindstedt et al. 2010, 2020).

Ultimately, determining the relative contribution of drift and selection and the relative importance of different selection pressures to the evolution of warning color requires integrating genetic, demographic, and ecological analyses. Despite several promising study systems (e.g., Galarza et al. 2014; Hegna et al. 2015; Lawrence et al. 2019), this level of integration remains rare with one notable exception: Müllerian mimics in the genus *Heliconius* (e.g., Merrill et al. 2015; Nadeau et al. 2016). These iconic butterflies have contributed substantially to our understanding of warning color evolution based on endogenously produced pigmentation. However, the extent to which lessons learned from *Heliconius* apply to other aposematic species remains unclear.

For example, key factors such as the life stage that expresses warning signals (larval vs. adult) (Willmott et al. 2011; Gaitonde et al. 2018) could have profound impacts on color evolution that are not typically considered in models for warning color evolution. Specifically, adult coloration of many aposematic taxa is often subject to positive frequency‐dependent selection via not only predator learning, but also positive assortative mating by color (Summers et al. 1999; Jiggins et al. 2001; Reynolds and Fitzpatrick 2007; Gordon et al. 2015). Color‐based mating preferences in the adults can impact warning coloration evolution in two ways. First, when reproductive adults mate assortatively by color and color varies among populations, gene flow will be reduced and genome‐wide differentiation can accumulate more readily between populations via genetic drift and divergent natural selection. Second, positive assortative mating by warning color may make it even more difficult for shifts in coloration to occur because rare color morphs will not only experience increased predation risk, but also reduced mating success (Jiggins et al. 2001; Naisbit et al. 2001). As a result, this can further strengthen selection against novel warning signal forms (Jiggins et al. 2001; Reynolds and Fitzpatrick 2007). By contrast, because coloration tends to be decoupled across ontogeny (Gaitonde et al. 2018; Galarza et al. 2019; Medina et al. 2020; Herrig et al. 2021; but see Lindstedt et al. 2016), aposematic larvae are not usually subject to direct sexual selection. Furthermore, aposematic larvae can occur in aggregations (Sillén‐Tullberg 1988; Terbot et al. 2017; Wang et al. 2021), which can weaken selection against novel signals (Mappes and Alatalo 1997; Riipi et al. 2001). Thus, all else equal, we hypothesize that the cost of being a rare aposematic color allele may be lessened in the larval stage, making the evolutionary shift to a novel color morph comparatively easier for larvae. Currently, we have very little data on how novel warning signals evolve and become abundant in immature stages in natural populations (Willmott et al. 2011), which prevents comparisons with the mechanisms found to be important in the adult stage.

Similarly, the source of color pigments—whether they are produced endogenously or acquired from the diet—can influence the relative importance of top‐down and bottom‐up selective agents acting on warning color variation (Grill and Moore 1998; Bezzerides et al. 2007; Blount et al. 2012). Bottom‐up selection may have an especially strong impact in herbivorous species, for which variation in host‐plant use across space can lead to differences in access to defensive compounds acquired from the host (Codella and Raffa 1995), the visual background against which coloration is displayed (Nosil et al. 2018), and access to diet‐derived pigments (Carroll et al. 1997). Although host shifts may also impact the availability of nutrients needed to produce pigments endogenously, warning signals based on endogenous pigments seem to be more robust and less variable under nutritional stress (Lindstedt et al. 2020). As a consequence, spatial variation in diet quality and nutrient availability is hypothesized to have a much stronger impact on diet‐derived warning signal pigmentation than on endogenously produced pigments (Blount et al. 2012). Yet, few studies have evaluated the contribution of diet quality to geographic variation in warning color pigmentation derived from the diet.

As a starting point to test these hypotheses and to address key gaps in currently available study systems, we investigated diet‐based warning color evolution in redheaded pine sawfly (*Neodiprion lecontei*) larvae. *Neodiprion lecontei* are specialist herbivores of pines and occur over a wide geographical and climatic range in eastern North America (Fig. 1). They are semisocial hymenopterans, meaning that larvae feed in large groups until the final instar, at which point they disperse from the feeding site to spin cocoons and pupate (Terbot et al. 2017). Based on their bright coloration, *N. lecontei* larvae are assumed to be aposematic. They defend against predators and parasitoids collectively using a synchronized display in which they raise their heads and regurgitate resinous droplets of sticky fluid sequestered from the host plant (Eisner et al. 1974; Codella and Raffa 1996; Costa 2006).

**Figure 1:**
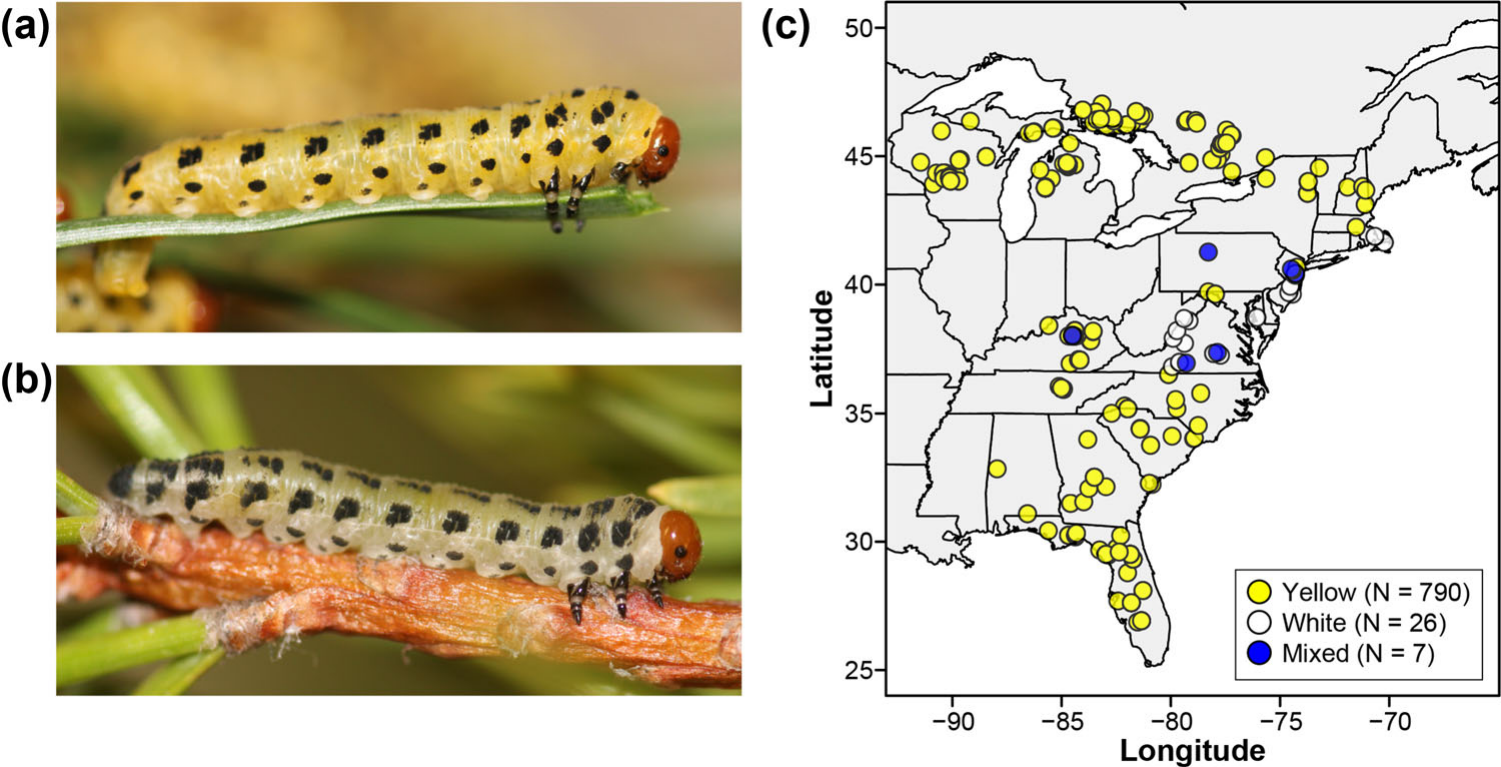
Appearance and distribution of white and yellow color morphs of *Neodiprion lecontei* larvae. Photographs depict representative yellow‐bodied (a) and white‐bodied (b) larvae (both photos by R. K. Bagley). (c) Approximate collecting locations and recorded color of 823 *N. lecontei* larval colonies collected between 2001–2004 and 2009–2016.

Throughout most of *N. lecontei*’s range, larvae have a bright, carotenoid‐based yellow body color overlaid with several rows of melanic black spots (Fig. 1) (Linnen et al. 2018). Like most insects, *N. lecontei* cannot synthesize carotenoids, which must therefore be obtained from the host plant. In addition, carotenoids are also thought to be involved in protecting the insect's own tissues from defensive toxins (scavenging free‐radicals) and in supporting immune defense (Cornet et al. 2007; Babin et al. 2010; Blount et al. 2012; Dhinaut et al. 2017; Koch et al. 2018). Thus, diet quality impacts *N. lecontei* fitness in multiple ways, including the availability of defensive terpene compounds (Codella and Raffa 1995) as well as acting as a direct source of carotenoids for conspicuous warning coloration (Linnen et al. 2018) and immune function. By contrast, *N. lecontei* adults are not aposematic, and expression of pigmentation genes is decoupled between larval and adult stages of this species (Herrig et al. 2021). Therefore, larval color is not likely to be subject to sexual selection.

Although most *N. lecontei* populations have yellow larvae, field surveys have revealed the presence of white‐bodied larvae in the eastern United States (Fig. 1). Previous demographic and genetic mapping analyses provide insight into the origin of this larval color variation. First, a range‐wide demographic analysis indicates that the white‐body phenotype is the derived state for *N. lecontei* larval pigmentation (Bagley et al. 2017). Second, a quantitative trait locus (QTL) mapping analysis of *N. lecontei* larval color suggests that the loss of yellow pigmentation in white‐bodied populations is attributable to small number of large‐effect loci that reduce or halt the transport of carotenoids from the gut to the integument (Linnen et al. 2018). Thus, although larvae obtain yellow pigments from their diet, the difference between white‐bodied and yellow‐bodied larvae is largely genetic, with a multiple‐QTL model explaining ∼86% of the phenotypic variation in recombinant F2 larvae derived from a cross between white‐bodied and yellow‐bodied populations (Linnen et al. 2018). Although previous studies have shed light on the origin of white‐bodied larvae, additional data are needed to explain how novel white‐bodied alleles persisted and spread in some *N. lecontei* populations.

To explain spatial variation in diet‐based larval warning color, we combined field surveys, predation experiments, population genomic analyses, and analysis of trait correlations. First, we compiled hundreds of field observations collected over 12 years to characterize the spatial distribution of the white‐bodied phenotype. Second, we combined avian vision modelling and predation assays to test if *N. lecontei* larvae are aposematic and how the white allele affects the efficacy of their warning coloration. Third, we used population genomic analyses to make inferences about the historical context of white allele spread and the evolutionary mechanisms responsible for among‐population variation in color. Fourth, we examined phenotypic correlations between larval color and other larval defense traits (immune function, chemical defense) to determine whether changes in larval coloration were likely to have correlated effects on other traits. Fifth, we combined field observations and chemical analysis of common pines to determine whether white‐bodied larvae are found disproportionately on low‐carotenoid hosts. We then integrated these data to make inferences regarding the most likely scenario under which the white allele spread (negative selection, neutrality, or positive selection), as well as the relative importance of top‐down and bottom‐up selection pressures. When contrasted with study systems involving aposematic adults that use endogenous warning pigments, our results suggest that life stage and pigment source may have profound impacts on warning color evolution.

## Materials and Methods

### GEOGRAPHIC DISTRIBUTION OF LARVAL COLOR IN *N. lecontei*

To describe the geographic distribution of the derived white‐body morph in *N. lecontei* larvae, we examined field notes and collection logs from multiple collecting trips that took place over 12 years (2001–2004 and 2009–2016). During this time, we collected 823 distinct *N. lecontei* larval colonies, each containing anywhere from a few individuals to over 100 individuals (Table S1). We used colonies—not individual larvae—as our unit of replication because each colony typically comprises the offspring of a single mated female. Because we consistently noted all colonies that were not uniformly yellow (color was assessed by eye), we could confidently assign each collected colony to one of three color categories: white‐bodied (*N* = 26), mixed‐color (*N* = 7), or yellow‐bodied (*N* = 790). To visualize the geographical data, we used R version 3.6 (R Core Team 2020) and the “maps” (version 3.3.0), “maptools” (version 0.9‐5), “mapplots” (version 1.5.1), and “mapdata” (version 2.3.0) packages.

### LARVAL COLOR AS AN ANTIPREDATOR DEFENSE

#### Conspicuousness of yellow‐ and white‐bodied N. lecontei larvae against different host plants

To determine if the color of *N. lecontei* larvae could function as a warning signal to predators (as has been assumed [Costa 2006]), we first asked whether birds can distinguish (1) the white‐ and yellow‐bodied larvae from each other and (2) the white‐ and yellow‐bodied larvae from their natural background. We quantified the conspicuousness of larvae against the three most common pine hosts for Central *N. lecontei*: *Pinus virginiana* (VA pine), *Pinus echinata* (shortleaf pine), and *Pinus rigida* (pitch pine) by using mathematical models that simulate the vision of an insectivorous avian predator (blue tit, *Cyanistes caeruleus*) (Vorobyev and Osorio 1998; Vorobyev et al. 1998). This approach assumes that the blue tit visual model available is representative of avian predators *N. lecontei* may encounter in North America.

Larvae used for these analyses were derived from a laboratory colony that was established from a single mixed‐color colony collected on an introduced host, *Pinus sylvestris*, in Piscataway, NJ (40° 32ʹ58.4ʺN, 74° 25ʹ50.9ʺ'W) in August 2013. For host material, we collected five clippings from each of three individual trees. All clippings were collected in July 2018 from the University of Kentucky Arboretum in Lexington, KY. This does not cover the whole geographical variation in color among host plants, but will give a rough estimate of the potential variation in contrast between the larval color and host plant species.

To quantify color of *N. lecontei* larvae and their host plants, we recorded reflectance spectra from each larval and host sample with a USB2000 spectrophotometer equipped with a 200‐μm probe and a PX‐2 pulsed xenon light source (Ocean Optics, Largo, FL). To minimize stray light, all reflectance measurements were taken in a dark, windowless room. Plant and larval spectra were recorded with SpectraSuite software (Ocean Optics) relative to a WS‐1‐SL reflectance standard (Ocean Optics). Each spectral measurement was taken with an integration time of 0.2 s, a boxcar width of 4, and 10 spectra averaged per measurement. For larvae, we recorded nine reflectance spectra across the dorsal, lateral, and ventral surfaces (three spectra per surface) of 20 individuals (10 white and 10 yellow) chosen at random from our laboratory population and immobilized with CO_2_. The reflectance probe was held at a 90° angle to each larva and as close to the larvae as possible, while still enabling us to ensure that we were recording measures from nonmelanic parts of the larval body. For host plants, we recorded 15 spectra across the new foliage (current year's growth), old foliage (previous years’ growth), and bark (five spectra per region) from each of 45 clippings (15 fresh clippings from each *Pinus* species). The reflectance probe was held at a 90° angle and as close as possible to the plant material. We used the program CLR: Color Analysis Programs version 1.05 (Montgomerie 2008) to bin and trim the raw spectra to 1‐nm intervals between 300 and 750 nm. We then averaged each bin for each sample to obtain a single summary spectrum for the dorsolateral (dorsal + lateral) and ventral surfaces of each larva and for the foliage and bark portions of each plant clipping. These averages were used in vision model analyses.

To predict whether a blue tit could discriminate between *N. lecontei* larvae and their natural pine backgrounds in terms of their color and luminance, we used a discrimination threshold model that assumes that noise in the receptors limits discrimination ability (Vorobyev and Osorio 1998; Vorobyev et al. 1998). The model uses information about the visual system, such as the sensitivity and relative abundance of different receptor types, and estimates of noise that arises in the photoreceptors. An average spectrum per each stimulus type was modeled for a blue tit's photon catch values for the single and double cones with a standard D65 irradiance spectrum. Color vision in birds stems from the four single cone types, whereas luminance‐based tasks apparently stem from the double cones (Osorio and Vorobyev 2005). For the color model, we therefore used the four single cones, whereas the luminance model was based on the double cones (Siddiqi et al. 2004). We used a Weber fraction of 0.05 for the discrimination model for the most abundant cone type and the relative proportion of cone types in the blue tit retina (long wave = 1.00, medium wave = 0.99, short wave = 0.71, and ultraviolet [UV] sensitive = 0.37).

Using the same approach as in Nokelainen et al. (2012), we calculated “just noticeable difference” (JND) values for every combination of larval body region (dorsolateral and ventral) and host background (foliage and bark for *P. virginiana*, *P. echinata*, and *P. rigida*) for each larva. For reference, JND values for prey/background combinations that are <1 are indistinguishable, values between <1 and 3 are hard to distinguish unless under optimal conditions, and values >5 are easy to tell apart under most conditions (Vorobyev and Osorio 1998).

#### Predator avoidance learning assays with white and yellow N. lecontei larvae

If *N. lecontei* larvae are aposematic, their unpalatability and conspicuousness should yield avoidance learning in potential predators. To test this prediction, we conducted avoidance learning experiments in plywood cages (plywood 50 × 50 × 70 cm [*w* × *d* × *h*]) at Konnevesi Research Station (described in, e.g., Lindstedt et al. 2008). Prey were offered through a hatch behind a visual barrier, which enabled us to record the exact time of prey detection because the bird had to go around the barrier or fly on top of it to see the prey.

Eleven great tits were tested with white *N. lecontei* larvae, 11 great tits with yellow *N. lecontei* larvae, and 10 great tits with light green *Diprion pini* larvae. *Diprion pini* were included as a cryptic control: although they are similarly chemically defended, they lack the conspicuous coloration of *N. lecontei* larvae (Lindstedt et al. 2011b) (Fig. S1). *Neodiprion lecontei* larvae were obtained from the same mixed‐color colony described above. After sorting by color (white or yellow), we froze live larvae at −80°C until needed. We used spectrophotometric measurements (collected as described above) to verify that human‐sorted larvae reflected true underlying differences in color and that color differences between thawed larvae that had been frozen recapitulated differences between living larvae (Table S2). *Diprion pini* larvae were obtained from laboratory insect culture described in Lindstedt et al. (2018) and killed by freezing. We thawed all larvae for 30 min prior to the experiments with birds. Because variation in larval defensive behaviors could affect how larvae appear to birds (see *Results* and also Lindstedt et al. 2018), we used dead larvae to isolate the effects of color and taste on bird behavior independent of larval behavior. Although we do not know how freezing and thawing impact taste, we note that birds readily ate similarly treated mealworms (see below). Due to the oily structure of the pine sawfly larvae's defensive fluid, the chemical composition of the fluid stored in the defensive glands is stable over long time periods (Eisner et al. 1974).

After birds were acclimated to the cage, *N. lecontei* larvae were offered one at a time and dorsal side up on a white dish (i.e., all the prey items were easy to detect for birds) in three consecutive trials similar to Lindstedt et al. (2008). At the beginning of each experiment, a bird's motivation to feed was confirmed by offering them a mealworm that had been killed by freezing and thawed prior to the experiment. To measure learning rate, we used attack latency (the time from when the bird noticed the prey to when it touched/attacked the prey with its beak [Lindstedt et al. 2008, 2011a]). Because hunger can affect a predator's readiness to attack defended prey (Sandre et al. 2010), we also quantified the hunger level as the mass of thawed mealworms eaten after the experimental trials.

We used delta attack latency, that is., change in attack latency in between first and last trial (trial 3 – trial 1 hesitation times), as a measure of avoidance learning. If *N. lecontei* larvae are aposematic, we expected to see an increase in attack latency between the first and third encounter, indicative of an increased reluctance to attack similarly colored prey once birds learned to associate their bright color with an unpleasant taste. For green and chemically defended *D. pini* larvae, we expected either no change or a less pronounced change in attack latency, indicative of reduced avoidance learning in the absence of a conspicuous color cue. We used a linear model (“lm” function from the base R package), because each bird had only one data point. Model diagnostics were performed on the residuals. We used planned contrasts to test for differences in delta attack latency: (i) between *D. pini* and (the average) *N. lecontei* and (ii) between white and yellow *N. lecontei*. To control for hunger level, the linear model included the amount of meal worms (mg) eaten after all trials as a covariate. We used the “summary” function to obtain the results table.

### POPULATION GENOMICS OF LARVAL COLOR

Assuming that a novel white allele would have been deleterious in a predominantly yellow‐bodied population, isolation and drift would have been required for the allele to persist and eventually reach high frequencies in some parts of the *N. lecontei* range. Such a scenario would have impacted not only color loci, but also loci distributed throughout the rest of the genome, giving rise to color‐associated population structure. Thus, although there is no way to definitively test whether the white allele was deleterious when it first arose, we can ask whether patterns of genomic variation are consistent with a historical scenario that would have facilitated the spread of a deleterious allele. To answer this question, we used double‐digest restriction‐associated DNA (ddRAD) sequencing to genotype white‐ and yellow‐bodied larvae collected across the eastern United States.

#### Sampling for population genomic analyses

A previous range‐wide analysis revealed that *N. lecontei* populations fall into three main genetic and geographic clusters that were isolated in different pine refugia during the Pleistocene, which were dubbed North, Central, and South (Bagley et al. 2017). Because white‐bodied populations are restricted to the Central cluster, we focused on increasing sampling in this region only to avoid confounding color‐associated divergence with refugia‐associated divergence. In total, we sampled larvae from 29 locations throughout the Central region, for a total of 65 larvae. This sample consisted of 19 white‐bodied larvae, 2 larvae from mixed‐color colonies, and 44 yellow‐bodied larvae (Table S3). Because colonies typically consist of siblings, each larva came from a different colony. Because *Neodiprion*, like all Hymenoptera, are haplodiploid and many of the analyses we used assume diploid data, we preferentially chose large larvae (which tend to be female) for DNA extraction and used genome‐wide heterozygosity estimates (–*het* option in vcftools (version 0.1.15 [Danecek et al. 2011]) to confirm that each individual was diploid (as in Bagley et al. 2017).

#### DNA extraction, library preparation, and genotyping

We extracted DNA and generated ddRAD sequencing libraries following protocols outlined in Bagley et al. (2017), but used a new set of sequencing adapters and PCR primers. Briefly, we digested DNA with the restriction enzymes *EcoRI* and *NlaIII*. We then ligated an adapter containing one of 48 unique 5 to 10 bp variable length barcodes to each sample (Burford Reiskind et al. 2016) (Table S4). Following pooling and size selection (379 ± 76 bp) on a PippinPrep (SageScience, Beverly, MA), we amplified libraries using high‐fidelity polymerase (Phusion; NEB, Ipswich, MA) with primers that incorporated an Illumina multiplexing index (Table S5), as well as a string of four degenerate bases for PCR duplicate detection. We then sequenced these libraries with 150‐bp reads on two replicate lanes of an Illumina HiSeq 4000 housed at the University of Illinois Roy J. Carver Biotechnology Center.

To quality‐filter and demultiplex raw sequencing reads, we used the default settings of the *process_radtags* module in stacks (version 1.46 [Catchen et al. 2013]). We then aligned the resulting reads to a high‐coverage, linkage‐group anchored *N. lecontei* genome assembly (version 1.1; GenBank assembly Accession no. GCA_001263575.2 [Vertanick et al. 2016; Linnen et al. 2018]) using the “very sensitive” end‐to‐end alignment mode in bowtie2 (version 2.3.1 [Langmead and Salzberg 2012]). We then used samtools (version 1.3 [Li et al. 2009]) to retain only uniquely mapping reads with Mapping Quality (MAPQ) scores ≥30. Putative PCR duplicates were identified based on the sequence of the four degenerate bases in the index read (provided as a second fastq file) and removed using a custom python script (defRemove_ddRAD_PCRduplicates.py, files in Supporting Information). We then constructed RAD loci from the filtered alignments in stacks’ *ref_map.pl* pipeline (version 1.46 [Catchen et al. 2013]). To ensure high‐confidence genotype calls (Kenny et al. 2010; Peterson et al. 2012), we required a minimum stack depth of 10 (*‐m* 10). We then used stacks’ *populations* module to call SNPs present in ≥70% of individuals (*‐r 0.7*). Depending on the analysis (see below), we also used stacks to retain SNPs with a minimum minor allele frequency of 0.01 (*–min_maf 0.01*) and/or to randomly sample one SNP per locus (–*write_random_snp* function in stacks) to minimize linkage disequilibrium between markers. Finally, as an additional step to remove paralogously mapping loci, we performed exact tests of Hardy‐Weinberg equilibrium in vcftools (version 0.1.15 [Danecek et al. 2011]) and excluded markers displaying significant heterozygote excess (*P* < 0.01). Summaries of the number of reads and loci per sample are included in Table S6.

#### Historical context of white allele spread

We evaluated the historical demographic context in which the white allele spread in two ways. First, we asked whether there was evidence of discrete population structure separating white‐bodied and yellow‐bodied larvae. The presence of strong color‐associated structure would be indicative of a corresponding historical event that could have facilitated the spread of a deleterious white allele, such as a rapid range expansion, population bottleneck, barrier to gene exchange, or rare long‐distance migration. To evaluate population structure, we used both model‐based and model‐free clustering approaches. For the model‐based approach, we used the program admixture (version 1.23 [Alexander et al. 2009])Alexander et al. 2009) to assign the proportion of ancestry for each individual from *K* ancestral populations. We performed 100 independent runs for each *K* value between *K* = 1 through *K* = 10. The optimal *K* was selected by comparing fivefold cross‐validation (CV) error values for each value of *K* as suggested in the admixture manual. We then used clumpak (version 1.1 [Kopelman et al. 2015]) to summarize and evaluate stability of assignment solutions across the 100 replicates of each *K*. For the model‐free approach, we used a discriminant analysis of principal components (DAPC) implemented using the *dapc* function in the adegenet r package (version 1.3‐9.2 [Jombart 2008; Jombart et al. 2010]). We identified the optimal number of clusters from *K* = 1 through *K* = 10 using a *K*‐means clustering algorithm. To avoid overfitting of discriminant functions, following α‐score optimization (Fig. S2), we performed this analysis using one principal component (PC) that explained ∼7% of total variation (Fig. S3). The clustering solutions were then compared using Bayesian information criterion (BIC), following Jombart et al. (2010). For both population‐structure analyses, we used an SNP dataset filtered on minor allele frequency (MAF >0.01) and linkage disequilibrium (LD, 1 SNP per RAD locus). This dataset contained 11,603 SNPs.

Second, we asked whether there was evidence to support a scenario in which a novel white allele spread by allele surfing, the rapid propagation of a new—and possibly deleterious—mutation at the edge of a range expansion via successive founder events (Edmonds et al. 2004; Peischl et al. 2013). This scenario predicts that there will be a decline in genetic diversity corresponding to the path of range expansion (due to successive founder events), with white‐bodied populations exhibiting reduced genetic diversity and located at the furthest edges of the range. To evaluate this prediction, we used the –*het* option in vcftools to compute observed heterozygosity for each individual from a SNP dataset that included all variable sites and no MAF filters (42,262 SNPs). To determine whether white‐bodied, mixed‐color, and yellow‐bodied colonies differ in heterozygosity, we used a Kruskal‐Wallis test (“kruskal.test” in R). To determine whether heterozygosity correlates with either latitude or longitude, we used a Spearman's rank correlation test (cor.test with “method = spearman”). Because we detected strong population structure between a western yellow‐only group and an eastern group with all colors (see below), we restricted our heterozygosity analyses to the eastern group to avoid confounding different sources of population structure.

#### Evolutionary processes maintaining color differentiation in extant populations

If body‐color alleles evolve neutrally, among‐population differentiation at color loci should reflect a balance between genetic drift and migration. Under this scenario, differentiation at color loci should be similar to neutral differentiation across the rest of the genome. Alternatively, if natural selection maintains geographic variation in larval color in the face of gene flow, differentiation at color loci should exceed genome‐wide levels of differentiation. To determine whether any SNPs exhibited evidence of selection between white‐bodied and yellow‐bodied populations collected from different geographic locations, we conducted an *F*_ST_ outlier analysis with BayeScan version 2.1 (Foll and Gaggiotti 2008). This method assumes a model in which subpopulations share a common migrant gene pool from which they differ due to varying degrees of isolation and drift, and uses a Bayesian approach to estimate the probability that each locus is under selection (Foll and Gaggiotti 2008). We converted the original VCF file to GESTE/BayeScan format using PGDSpider (version 2.1.1.5 [Lischer and Excoffier 2011]). Our pilot analysis consisted of 20 runs with 5000 iterations each; and our Markov chain was run for a total of 100,000 iterations, with a burn‐in of 50,000 generations, a thinning interval of 10, and a prior odds ratio of 100. To maximize our chances of retaining informative SNPs in linkage disequilibrium with a color locus, we retained all SNPs with MAF > 0.01 (38,852 SNPs). As in the heterozygosity analysis, we restricted this analysis to the eastern mixed‐color group.

### CORRELATION BETWEEN LARVAL COLOR AND OTHER DEFENSIVE TRAITS

Because dietary carotenoids serve several important functions in insects, there may be trade‐offs between the use of carotenoids for warning coloration and allocation to essential functions such as immunity and protection of an insect's own tissues from the toxic compounds used in chemical defense (Cornet et al. 2007; Blount et al. 2009, 2012; Babin et al. 2010; Dhinaut et al. 2017). This hypothesis predicts that there will be genetic correlations between larval body color and other carotenoid‐related traits. To test this prediction, we evaluated trait correlations in recombinant F_2_ progeny produced from a cross between *N. lecontei* females from a laboratory line derived from a white‐bodied population (Valley View, VA; 37°54ʹ47ʺN, 79°53ʹ46ʺW) to males from a laboratory line derived from a yellow‐bodied population (Bitley, MI; 43°47ʹ46ʺN, 85°44ʹ24ʺW) (same cross as in Linnen et al. 2018, but different families and individuals). Because *Neodiprion* are haplodiploid, these crosses generated hybrid, diploid F_1_ females and nonhybrid haploid males. To generate haploid F_2_ males, we reared the offspring of virgin F_1_ females. The haploid F_2_ male offspring, the products of recombination in virgin hybrid F_1_ females, therefore represent the whole diversity of different combinations of color alleles and their associated traits. This allowed us to test whether white/yellow alleles are likely to have correlated effects on other defense traits through pleiotropy or linkage (i.e., whether there are genetic correlations). Because estimation of genetic correlations requires prohibitively large sample sizes, we used phenotypic correlations as a proxy (Cheverud 1988; Roff 1996; but see Willis et al. 1991). Although the magnitude of phenotypic correlations should be interpreted with caution because they can differ substantially from genetic correlations (Kruuk et al. 2008), they are nevertheless informative with respect to the presence and direction of underlying genetic correlations (Roff 1996; Kruuk et al. 2008; Dochtermann 2011; but see Hadfield et al. 2007).

All larvae were fed *Pinus banksiana* (jack pine) foliage ad libitum. Conditions for the insect cultures are described in Linnen et al. (2018). We measured color as a proxy for carotenoid content and defensive traits (presence, quantity, and quality of defensive regurgitant; encapsulation response) in *N* = 212 F_2_ male larvae from 10 F_1_ mothers. We measured these traits in mature feeding instars only (Linnen et al. 2018).

#### Carotenoid content of recombinant F_2_ males

To estimate carotenoid content for each larva, we took five measurements with the spectrophotometer from each of three body regions: the dorsum, lateral side, and ventrum. All other details of spectra collection were as described above. We then used the program CLR: Color Analysis Programs version 1.05 (Montgomerie 2006) to process raw spectra and compute color saturation (S1B) values for each larva, which correlate negatively with carotenoid content in multiple taxa (Butler et al. 2011). To obtain a single S1B value per larva, we averaged across the 15 reflectance spectra.

#### Chemical defense of recombinant F_2_ males

To evaluate larval chemical defense, we gently poked each larva twice with a capillary tube (the capillary tube did not break the skin of the larva) between the front legs on the ventral side and recorded presence/absence of a defensive regurgitant (see Lindstedt et al. 2018). For consistency, all pokes were performed by the same researcher and larva were chosen at random (without respect to color) from their rearing boxes. If a defensive regurgitant was produced, we collected it in a 5‐μL capillary tube. To estimate defense fluid quantity, we measured the length of the regurgitant in the capillary tube with digital calipers. To control for the effect of body size on the amount of defense fluid produced, we also measured larval body length. Larval defense fluid samples in capillary tubes were placed in 1.5‐mL microcentrifuge tubes containing 500 μL *n*‐hexane and stored in a −20°C freezer until analysis.

To determine monoterpenes and other terpene compounds in the defensive regurgitates, a Shimadzu, GC‐2010 Plus (Shimadzu Corp., Kyoto, Japan) gas chromatograph fitted with a flame ionization detector (FID) was used. Samples dissolved in hexane were injected (splitless injection of 1 μL sample, inlet temperature 290°C) into a Zebron ZB‐5MSi capillary column (length 30 m, inner diameter 0.25 mm, film thickness 0.25 μm; Phenomenex Inc., Torrance, CA). Helium was used as the carrier gas at a flow rate of 1 mL min^−1^ (83.6 kPa). The temperature program started at 50°C (1.5‐min hold) and the column oven was heated with a rate of 10°C min^−1^ to 180°C, and then at a rate of 2.5°C min^−1^ to 290°C (10‐min hold). The temperature of FID was 290°C. We used L‐fenchone as an internal standard. We identified the compounds with separate runs using a GC equipped with a mass spectrophotometer (Shimadzu 2010 GC/MS; Shimadzu Corp., Kyoto, Japan) and otherwise similar conditions as in the GC/FID analyses. For the identification of terpenes, we compared the mass spectra (electron ionization 70 eV) of the obtained peaks to those of the library. We used the retention times of terpene peaks in GC/MS chromatograms to assign the compounds in GC/FID chromatograms. Masses (μg) of different compounds in the defense fluid samples were calculated by dividing the area of a compound's peak by area of the internal standard's peak, and then multiplying it with the concentration of the internal standard (20 ppm). Concentration (%) of monoterpene compounds (total mass of all the monoterpene compounds/sample) and other terpene compounds (total mass of all the other terpene compounds/sample) in the defense fluid sample was calculated with the following formula: (HPLC value in mg/defense fluid sample size [μL]) × 100.

#### Encapsulation response of recombinant F_2_ males

To evaluate immune defense, we measured encapsulation response as described in Lindstedt et al. (2011b) after chemical defense measurements were taken. Larvae were first anaesthetized with CO_2_ (Lindstedt et al. 2018). A sterilized hypodermic needle was then used to puncture the skin on the dorsal part of the individual and a nylon implant (length 3 mm, diameter 0.11 mm) was inserted into the resulting hole. After 24 h, the implant was removed, dried, and photographed under a Zeiss DiscoveryV8 microscope equipped with an Axiocam 105 camera and ZEN lite 2012 software (Carl Zeiss Microscopy, LLC, Thornwood, NY). Every implant was photographed three times from three different angles (Rantala et al. 2000). Image analysis software ImageJ 1.47v (National Institutes of Health, USA) was then used to quantify the gray value of the implant, which was used as a measure of the encapsulation reaction. The gray value of the background was subtracted from the gray value of the implant to correct for potential variation in light source. The darker the implant, the stronger the immune response.

#### Statistical analyses for phenotypic correlations

Phenotypic correlations between color (carotenoid content), chemical defense (defensive behavior, quantity, and terpene concentration of defense fluid), and immune defense (encapsulation response) were evaluated in R (version 3.6.2 [R Core Team 2020]). We used generalized linear mixed models using the function “glmer” from the package “lme4” (version 1.1‐23 [Bates et al. 2014]) and linear mixed models using the function “lme” from the package “nlme” (version 3.1‐147 [Pinheiro et al. 2020]). For all models, colony identity was included as a random effect and the continuous predictors were centered, so that the intercept term estimates the outcome variable at the average predictor levels. Chemical concentration variables were modeled using total volume as an offset term and Gaussian errors. Encapsulation response was also modeled using Gaussian errors. To account for large skew and excess zeros in defense fluid quantity, this trait was transformed (0.00001 added to all data points) and modeled using Gamma family. As a binary outcome variable, the probability of defense was modeled using binomial family.

### CORRELATION BETWEEN LARVAL COLOR AND HOST‐PLANT CAROTENOID CONTENT

Because the yellow body color of *N. lecontei* larvae is derived from carotenoids acquired from the host plants (Linnen et al. 2018), a reduction in carotenoid availability in the larval diet (adults are nonfeeding) could potentially favor the loss of yellow body color in some populations. To evaluate this possibility, we first asked whether the prevalence of white‐bodied colonies differed among host plant species. To do so, we examined color and host species for the 823 colonies described in “GEOGRAPHIC DISTRIBUTION OF LARVAL COLOR IN *N. Lecontei*” section. We restricted our focus to the Central region only and, within this region, the three primary pine hosts for which we had substantial sample sizes: *P. echinata*, shortleaf pine (*N* = 63 colonies); *P. rigida*, pitch pine (*N* = 50 colonies); and *P. virginiana*, Virginia pine (*N* = 167 colonies). To determine whether the counts of different colony colors differed among the three hosts, we used a Fisher's exact test (“fisher.test”) followed by post hoc tests (“fisher.multcomp” function from the RVAideMemoire version 0.9‐73 R package).

Because the prevalence of white‐bodied colonies differed among the three pine hosts (see *Results*), we next asked whether the carotenoid content of these hosts differed. To measure carotenoid content in *P. rigida*, *P. echinata*, and *P. virginiana*, we sampled ∼10–20 needles from each of the 45 clippings (five clippings from three individual trees per species) that were used to measure host reflectance spectra (see “*Conspicuousness of yellow‐ and white‐bodied* N. lecontei *larvae against different host plants*” section). We then cut the needles into 2‐ to 5‐mm segments and stored them in 1.7‐mL microcentrifuge tubes in the −20°C freezer until pigment extraction. To extract pigments and quantify carotenoid content, we followed protocols outlined in Minocha et al. (2009). First, we placed 15.0 mg (± 0.3 mg) of chopped needle into a 2.0‐mL tube, to which we added 1.5 mL of 200‐proof ethanol. Next, samples were vortexed and stored in the dark at 65°C for 24 h. After cooling to room temperature, samples were vortexed for approximately 1 min at medium speed and centrifuged at 13,500 × *g* (rcf) for 5 min. For each sample, we then transferred 200 μl of the supernatant to each of three wells (replicates, to reduce measurement error) in a clear, flat‐bottomed 96‐well plate. Using a Synergy HT plate reader (Biotek Instruments, Winooski, VT) and Gen5 software, we read the absorbance of each sample at 664, 649, and 470 nm. We then used these values and an equation for ethanol solvent (Lichtenthaler 1987; Minocha et al. 2009) to compute carotenoid concentration. We used the *lme4* (version 1.1‐21), *lmertest* (version 3.1‐1), and *multcomp* (version 1.4‐12) R (version 3.6.2) packages to fit a linear mixed model to the data (with host species and individual tree as fixed and random effects, respectively) and, because the host effect was significant, to compare pairs of hosts with post hoc Tukey contrasts.

## Results

### WHITE‐BODIED LARVAE ARE RESTRICTED TO THE EASTERN COAST OF THE UNITED STATES

Our collecting records (summarized in Table S1) confirmed that all‐white colonies are restricted to the east of the Appalachian Mountains, primarily in coastal states in the eastern United States (Fig. 1c). Although some states had records of both all‐white and all‐yellow colonies, these were never collected at the same site. Mixed‐color colonies were rare and 4 out of 7 were collected on nonnative pines in disturbed areas (e.g., parking lots). Notably, a single mixed‐color colony was collected west of the Appalachians. This mixed‐color colony was the only colony out of 278 colonies collected in Lexington, KY to contain any white‐bodied larvae (Table S1). Given the rarity of white larvae in the area, the mixed‐color colony may have been the result of a rare long‐distance migration event or a de novo mutation. Overall, these results reveal that much of the variation in larval color is distributed among rather than within populations.

### WHITE AND YELLOW *N. lecontei* LARVAE ARE APOSEMATIC

**Figure 2:**
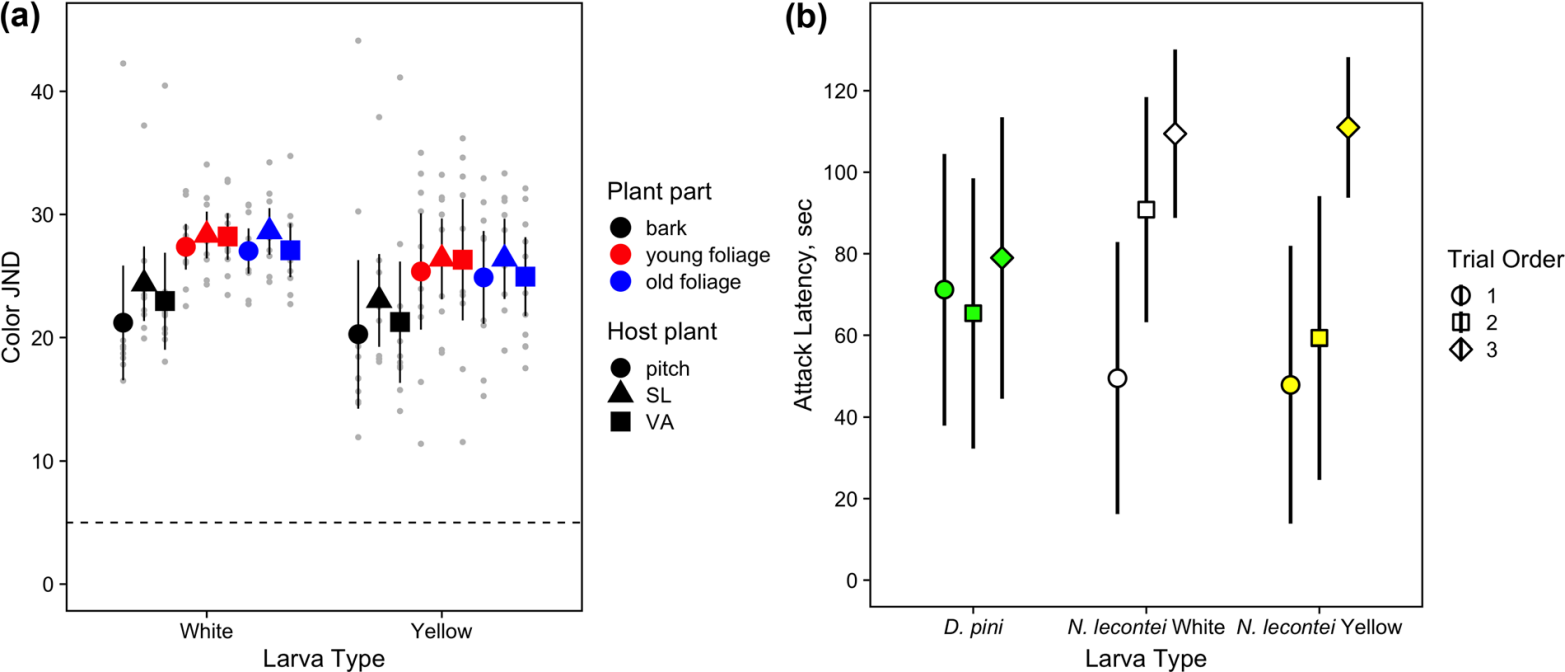
White and yellow *Neodiprion lecontei* larvae are aposematic and do not differ in signal efficacy. (a) Color contrasts against different host plants and host‐plant parts. For reference, JND values for prey/background combinations that are <1 are indistinguishable, values between <1 and 3 are hard to distinguish unless under optimal conditions, and values >5 are easy to tell apart under most conditions (Vorobyev and Osorio 1998). Dashed line shows the threshold value for JND = 5 above which objects should appear clearly conspicuous for blue tits (*Cyanistes caeruleus*). Changes in attack latency (seconds) (b) across three trials for three different types of pine sawfly in avoidance learning assays. Error bars are 95% confidence intervals.

Regardless of the larval body color (white or yellow), body region, host species, and host tissue type, *N. lecontei* larvae were highly conspicuous against a pine background in terms of color contrast (all JND values > 5; Fig. 2a). Most larval/host combinations were also highly conspicuous in terms of luminance, but some individual larvae had JND < 5 for some host background types (Fig. S4). These results demonstrate that a blue tit could distinguish between larvae and their host plant, which supports the hypothesis that *N. lecontei* larvae are conspicuous to their predators. Moreover, blue tits should be able to distinguish between white larvae and yellow larvae (color contrast JND 7.4, luminance contrast JND 3.6).

We also found that captive great tits learned to avoid brightly colored white and yellow *N. lecontei* larvae more quickly than light green and chemically defended *D. pini* sawfly larvae. Specifically, the change in the mean attack latency from the first to third trial was significantly larger for *N. lecontei* larvae than for *D. pini* larvae (Table 1; Fig. 2b). By contrast, predators learned to avoid white and yellow *N. lecontei* larvae equally quickly (Table 1; Fig. 2b). Combined with our finding that both morphs are conspicuous to an avian predator against pine backgrounds (Fig. 2a), our predation assay results suggest that white and yellow larvae do not differ in signal efficacy. Finally, hunger level of birds did not affect significantly attack latencies (Table 1).

**Table 1:** Predator response with respect to larval type. Output table from a linear model (*y* = change in attack latency, gaussian errors, *t* statistic) using planned contrasts (*D. pini* vs. pooled *N. lecontei*; white vs. yellow *N. lecontei*)

Change in attack latency	Estimate	SE	*T* or *z*	*P*
Intercept	11.428	22.778	0.502	0.6200
*D. pini* vs. *N. lecontei*	56.175	23.972	2.348	0.0265
White *N. lecontei* vs. Yellow *N. lecontei*	−4.356	25.668	−0.170	0.8665
Hunger (mg mealworms eaten)	−14.043	33.213	−0.423	0.6758

### HISTORICAL DEMOGRAPHY WOULD NOT HAVE FACILITATED THE SPREAD OF A DELETERIOUS ALLELE, AND DIFFERENTIATION AT A SINGLE COLOR LOCUS IS ELEVATED BETWEEN WHITE‐BODIED AND YELLOW‐BODIED POPULATIONS

**Figure 3:**
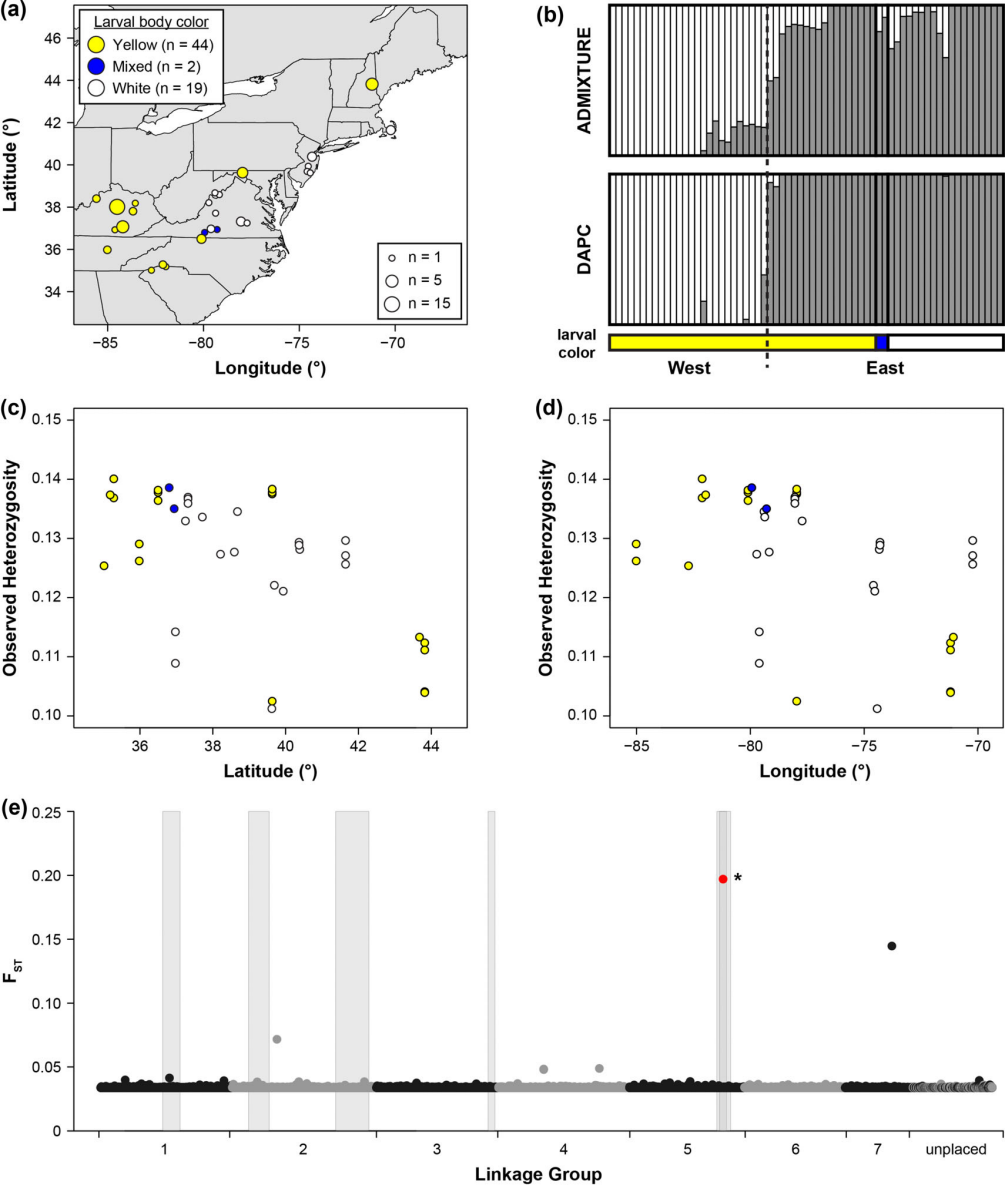
Genome‐wide markers reveal no evidence of color‐associated population structure in *Neodiprion lecontei*, but differentiation at a single color locus is elevated between white‐bodied and yellow‐bodied populations. (a) Color, location, and sample size for 65 individuals and 29 locations included in the population structure analyses. Points are colored based on the color of all larvae collected at that location, with size of the point reflecting number of individuals (colonies) sampled. (b) Inferred ancestry for each individual sorted by the color of the colony from which it was collected under a model of *K* = 2 based on the program admixture (top) and *adegenet* (bottom). Colors are denoted by the “larval color” bar, with colors indicated as in (a). Within each color, individuals are sorted in geographical order. The “West” genetic cluster contains only yellow larvae, whereas the “East” genetic cluster contains yellow, white, and mixed‐color colonies. (c) Relationship between observed heterozygosity and latitude for “East” individuals with colony color indicated as in panel A. Heterozygosity declines with latitude, and the lowest‐heterozygosity individuals are from yellow‐bodied populations collected in the northernmost part of the range. (d) Relationship between observed heterozygosity and longitude for “East” individuals with colony color indicated as in panel A. Heterozygosity declines with longitude. (e) Per‐site differentiation (*F*_ST_) between white‐bodied and yellow‐bodied populations of *N. lecontei* (“East” populations only) across seven linkage groups and remaining unplaced scaffolds. Shaded gray boxes denote the locations of six QTL associated with larval body color in a cross between white‐bodied and yellow‐bodied populations (Linnen et al. 2018). The red point and asterisk indicate the only significant *F*_ST_ outlier, which falls within two major‐effect QTL intervals.

Both model‐based and model‐free analyses of discrete population structure recovered *K* = 2 as the optimal number of clusters (Fig. S5). However, assignment patterns under *K* = 2 corresponded to geographical regions, not color. Specifically, individuals west of the Appalachian Mountains (“West”; i.e., those in Kentucky) belonged to one cluster and individuals sampled from within or east of the Appalachian Mountains assigned to the other (“East”) (Fig. 3a, b). Individual assignments were stable across all 100 admixture runs, and similar for most individuals between the admixture and DAPC methods (Fig. 3b). Thus, there is no discrete population structure indicative of a historical event that separated white‐bodied and yellow‐bodied populations.

In larvae collected from the “East” cluster, we found no relationship between heterozygosity and larval colony color (Kruskal‐Wallis chi‐squared = 3.17, df = 2, *P* = 0.20). Consistent with a post‐glacial range expansion to the north and east from a southern refugium, we found a significant decline in heterozygosity with both latitude (Spearman's rho = −0.51; *P* = 0.00084; Fig. 3c) and longitude (Spearman's rho = −0.46; *P* = 0.0032; Fig. 3d). However, the low‐heterozygosity populations at the northern edge of the range were yellow, not white (Fig. 3a, c). These data do not support an allele‐surfing scenario for the white allele. Instead, these data are consistent with a scenario in which the white allele arose and spread sometime after the eastern range expansion was complete.

Our *F*_ST_ outlier analysis yielded a single SNP on chromosome 5 (position 21793198) with significantly elevated differentiation between white‐bodied and yellow‐bodied populations (*F*_ST_ = 0.197, *q*‐value = 0.012; Fig. 3e). Notably, this SNP fell within the intervals of two QTL peaks previously identified for larval body color in *N. lecontei*, including one QTL that explains ∼52% of the difference in color between white‐ and yellow‐bodied populations (Linnen et al. 2018). This finding is consistent with the hypothesis that differences in pigmentation among *N. lecontei* populations are currently maintained by selection in the face of gene flow. We note, however, that our *F*_ST_ outlier results should be interpreted with caution because the demographic history of white‐bodied and yellow‐bodied populations likely deviates from the simple model assumed by BayeScan. Although this method is generally robust to more complex demographic scenarios (Foll and Gaggiotti 2008), additional data are needed to evaluate the impact of violating BayeScan assumptions on the rate of false positives and false negatives and to apply alternative tests of selection.

### LARVAL BODY COLOR DOES NOT CORRELATE WITH OTHER DEFENSIVE TRAITS

Our analysis of over 200 recombinant haploid males revealed that there was no correlation between body color (a proxy for carotenoid content) and any of the defensive traits (behavioral, chemical, immune) measured (Table 2). Together, these results do not support the hypothesis that the loss of yellow pigmentation was favored by natural selection because it enabled white‐bodied larvae to allocate more resources to defense and immune functions (but see *Discussion*).

**Table 2:** Correlation between a proxy for carotenoid concentration (S1B) and defensive behavior, quality and quantity of chemical defense against predators, and encapsulation response (defense against parasitoids). *t*‐statistic for Gaussian error LMMs (B, C, and E) and *z*‐statistic for GLMMs (A and D)

	Estimate ± SE	*t* or *z*	*P*
A: Defensive fluid volume (*N* = 91)			
Intercept	0.603 ± 0.251	2.40	0.0163
Body length	−0.072 ± 0.094	−0.76	0.445
S1B	1.506 ± 1.930	0.78	0.435
B: Monoterpene content of defensive fluid (per μL of fluid) (*N* = 66)			
Intercept	0.074 ± 0.024	6.20	<0.0001
S1B	0.084 ± 0.097	0.86	0.391
C: Total terpene content of defensive fluid (per μL of fluid) (*N* = 66)			
Intercept	0.118 ± 0.017	7.14	<0.0001
S1B	0.177 ± 0.130	1.36	0.180
D: Defensive behavior (deploys fluid or not) (*N* = 91)			
Intercept	1.94 ± 0.45	4.34	<0.0001
S1B	3.99 ± 4.62	0.864	0.388
E: Encapsulation response (*N* = 83)			
Intercept	51.21 ± 8.71	5.88	<0.0001
S1B	−13.39 ± 36.42	−0.368	0.714

### WHITE‐BODIED LARVAL COLONIES ARE ASSOCIATED WITH LOW‐CAROTENOID HOSTS

**Figure 4:**
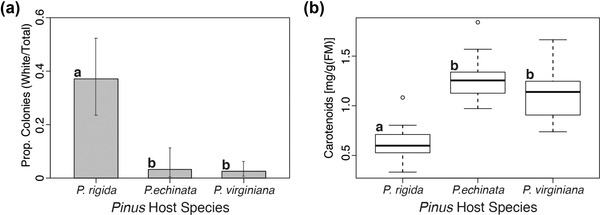
Relationship between host use and larval body color in *N. lecontei*.  (a) Compared to *Pinus echinata* and *P. virginiana*, *P. rigida* had a significantly higher proportion of white‐bodied larval colonies. Error bars are Clopper‐Pearson 95% confidence intervals. (b) Compared to *P. echinata* and *P. virginiana*, *P. rigida* foliage had significantly lower carotenoid content. In all panels, letters indicate significant pairwise differences between colony color or host plants at *P* < 0.05.

The proportion of colonies that were white bodied differed significantly across host plants (Fisher's exact test, *P* < 1 × 10^−9^), with white‐bodied colonies significantly more common on *P. rigida* than on either *P. echinata* (*P* = 9.6 × 10^−6^) or *P. virginiana* (*P* = 2.4 × 10^−9^) (Fig. 4a). Prevalence of white‐bodied colonies did not differ between *P. virginiana* and *P. echinata* (*P* = 0.66). We also found that the three hosts differed significantly in carotenoid content (*F*_2_ = 20.12, *P* = 0.0022), with *P. rigida* having the lowest carotenoid content of all three hosts (Fig. 4b; *P. rigida* vs. *P. echinata*: *z* = 6.108, *P* = 3.02 × 10^−9^; *P. rigida* vs. *P. virginiana*: *z* = 4.538, *P* = 1.13 × 10^−5^; *P. echinata* vs. *P. virginiana*: *z* = −1.570, *P* = 0.116). Notably, although *P. rigida* is widely distributed throughout the eastern United States, it is most abundant in the Atlantic Coastal plain (Gleason and Cronquist 1991; Critchfield and Little 1966), where white‐bodied populations are predominantly found (Fig. 1c). Overall, these results support the hypothesis that an increased tendency to use a low‐carotenoid host could have favored the spread of novel white‐body alleles in the eastern United States.

## Discussion

Here, we investigated the evolutionary processes and selective pressures shaping geographic variation in body color in conspicuously colored *N. lecontei* larvae. We first confirmed that what we consider to be aposematic coloration does, in fact, confer protection against visual predators of *N. lecontei* larvae. Therefore, it is possible that *N. lecontei* larval coloration evolves under positive‐frequency‐dependent selection, favoring familiar color morphs over novel color morphs (e.g., Kapan 2001; Chouteau et al. 2016). Indeed, as expected under purifying selection against novel warning color alleles, local populations of *N. lecontei* larvae tend to be monomorphic for color (Fig. 1c). However, in contrast to many studies on aposematic adult coloration (Summers et al. 1999; Jiggins et al. 2001), a lack of color‐associated population structure (Fig. 3) suggests that the demographic context in which the white allele arose would not have been conducive to the spread of a deleterious allele. Additionally, our finding that differentiation at a single SNP linked to a previously identified color locus exceeded genome‐wide levels of genetic differentiation between white‐ and yellow‐bodied populations (Fig. 3) suggests that geographical differences in color among extant populations are attributable to natural selection rather than migration‐drift balance. Together, these results suggest that for aposematic larvae, genetic drift need not be invoked to explain shifts in warning color.

Having determined that white‐body alleles could have evolved under positive selection, we next examined sources of selection that could have potentially offset (or exacerbated) the cost of being rare. Perhaps unexpectedly, we did not find any evidence that the loss of yellow pigmentation impacts defense against natural enemies. Body color had no impact on the efficacy of the warning signal (Fig. 2b). We also found no evidence that white‐body alleles have indirect effects on chemical defense or immune function via pleiotropy or physical linkage (Table 2). Instead, we found that white‐bodied larvae were disproportionately abundant on a pine species with low carotenoid content (Fig. 4). Together, these findings suggest that bottom‐up selection via host‐plant quality may play an important role in driving warning color polytypism in immature stages that rely on diet‐derived pigmentation. Overall, these results suggest that life stage and pigment source can greatly impact the evolutionary processes and selective pressures shaping warning color variation. To explore this idea further, we contrast our results with other aposematic study systems and highlight priorities for future work on this and other aposematic systems.

The lack of color‐associated population structure in *N. lecontei* contrasts with several other aposematic study systems that implicate geographic isolation and genetic drift as key facilitators of polytypic warning signals. For example, in *Heliconius* butterflies, genetic drift is thought to promote the establishment of novel wing pattern variants in accordance with Wright's shifting balance model (Wright 1948; Mallet and Singer 1987; Mallet 2010) (but see Brown et al. 1974; Sheppard et al. 1985; Turner et al. 1996). In support of this hypothesis, multiple *Heliconius* species have pronounced population genetic structure indicative of historical subdivision and drift (Kronforst and Gilbert 2008). Color‐associated population structure has also been detected in other aposematic species, such as the strawberry poison frog (*Oophaga pumilio*) (Wang and Summers 2010) and the polytypic dyeing dart poison frog (*Dendrobates tinctorius*) (Lawrence et al. 2019). One important difference between *N. lecontei* and many other aposematic study systems is the role of warning coloration in mate choice. Although larval color variation in *Neodiprion* sawflies is unlikely to have any impact on mate preferences in the non‐aposematic adults, positive assortative mating based on the color of aposematic adults has been demonstrated experimentally in both *Heliconius* (Jiggins et al. 2001; Naisbit et al. 2001; Merrill et al. 2014) and *O. pumilio* (Reynolds and Fitzpatrick 2007). The absence of this added barrier to evolving a novel color morph may explain, in part, why a shift in *N. lecontei* larval coloration seemingly did not require external factors to increase isolation and drift.

In addition to a lack of sexual selection, several other features of larval defensive displays in *N. lecontei* (and other taxa) have the potential to reduce the strength of purifying selection against novel warning color alleles, thereby facilitating their spread in the absence of isolation and genetic drift. First, *N. lecontei* larvae present their defensive chemicals externally (Codella and Raffa 1995; Costa 2006), a strategy that has been shown to reduce attack rates against novel conspicuous prey as predators can estimate a prey individual's defensive capacity before tasting it (Halpin et al. 2008). Second, both yellow and white *N. lecontei* larvae have melanin‐based black spots across their body, which predators could use as an additional signal of unprofitability together with the bright pigmentation (but see Aronsson and Gamberale‐Stille 2008). This similarity in one aspect of the aposematic pattern could have decreased initial costs of rarity for a white larval form, thereby facilitating the shift from yellow to white pigmentation via generalization (Balogh et al. 2010; Lawrence et al. 2019; but see Rönkä et al. 2018b). Third, and perhaps most importantly, the costs of being a low frequency color morph could be mitigated by gregariousness in chemically defended prey. *N. lecontei* larvae feed in large aggregations, which have been shown to increase avoidance learning efficacy and initial wariness of predators toward both conspicuous and cryptic unprofitable prey (Sillén‐Tullberg 1990; Alatalo and Mappes 1996; Riipi et al. 2001). Gregariousness could have therefore facilitated shifts in warning coloration among *N. lecontei* populations. Additional experiments are needed to determine the interactive effects of predator's generalization, larval group size, larval defensive displays, and color. To assess whether predation risk for yellow‐ and white‐bodied larvae varies among *N. lecontei* populations, future research should also test for differences in the survival of white‐ and yellow‐bodied larvae across their geographical range and across different visual backgrounds (Rojas et al. 2014; Rönkä et al. 2020).

In terms of possible benefits that could have facilitated the spread of novel white‐body alleles via positive selection, our results suggest that geographic variation in the abundance of a low‐carotenoid host (*P. rigida*) may have favored the loss of carotenoid‐based larval coloration in some *N. lecontei* populations. Thus, rather than strong top‐down selection by predators and pathogens, bottom‐up selection via host‐plant characteristics may have driven shifts in warning color among *N. lecontei* populations. This finding contrasts with strong top‐down selection pressures that may explain endogenously produced polymorphic and polytypic warning color in *Arctia plantaginis* (Rönkä et al. 2020) and polytypic warning color in *Heliconius melpomene*. Historical demographic analyses suggest that *H. melpomene* radiated into areas already occupied by another, more abundant aposematic species, *H. erato* (e.g., Turner et al. 1996; Mallet and Joron 1999; Kronforst and Gilbert 2008; Quek et al. 2010). Although warning color polytypisms were thought to have evolved via drift in *H. erato*, later radiating *H. melpomene* are thought to have evolved under positive selection to match locally abundant *H. erato* color phenotypes (Kronforst and Gilbert 2008; Quek et al. 2010). Consistent with this “advergence” hypothesis, early‐radiating *erato*‐clade species exhibit much more pronounced population substructure than *melpomene*‐clade species (Kronforst and Gilbert 2008). Like *N. lecontei*, *H. melpomene* exhibits geographic variation in host use (Smiley 1978; Brown 1981). But unlike *N. lecontei*, *Heliconius* butterflies primarily rely on endogenous pigments for warning coloration (reviewed in Nadeau 2016). Although these differences are intriguing, comparative analysis of color evolution in diverse taxa is needed to test our hypothesis that pigment source impacts the primary selective agents shaping pigment variation.

In the *N. lecontei* system, additional experimental work is also needed to clarify causal links between differences in host use and differences in fitness between *N. lecontei* color morphs. Our observation that *P. rigida*, a low‐carotenoid host, harbors disproportionately more white‐bodied larvae (Fig. 4) is consistent with the hypothesis that white alleles were favored due to carotenoid limitation. However, pine species differ in other characteristics besides carotenoid content that could potentially favor different carotenoid allocation strategies in the larvae. For example, differences in pine species color result in differences in larval luminance contrast values (Fig. S4). Nevertheless, larvae are conspicuous on all three hosts and therefore host color seems unlikely to drive color divergence among larval populations. *Pinus rigida* could also differ from the other pine species in nutritional profile, secondary compounds (terpene content), and/or abiotic environments in ways that increase the demand for antioxidant functions of carotenoid compounds (Blount et al. 2009). However, based on previous studies, *N. lecontei* seem relatively insensitive to variation in the host‐plant terpene content (Codella and Raffa 1995).

If carotenoid limitation is the primary selection pressure favoring white body color, there are two nonmutually exclusive mechanisms through which host carotenoid content could differentially impact the fitness of yellow and white *N. lecontei* larvae. First, production of conspicuous yellow coloration could be constrained under a carotenoid‐limited diet, potentially weakening the signal efficacy (saturation and brightness) of yellow genotypes that develop on a low‐carotenoid host. Second, because carotenoids are also involved in physiological processes such as immune defense (Cornet et al. 2007; Babin et al. 2010; Blount et al. 2012; Dhinaut et al. 2017; Koch et al. 2018), trade‐offs between warning signaling and these other functions could result in divergent selection on pigmentation on different host plants. These trade‐offs and constraints should be most evident when the availability of pigments or their precursors are limited in the diet (Lindstedt et al. 2020). However, such trade‐offs should also generate negative genetic (and phenotypic) correlations between levels of carotenoid‐based pigmentation and other fitness traits (especially immune or antioxidant function), which we did not find in this study (Table 2; see also Siiri‐Lii et al. 2007). That said, an important limitation of our data is that we did not measure trait correlations (trade‐offs) in larvae that developed on *P. rigida*. Because both diet‐derived pigmentation and defensive compounds (Blount et al. 2012) as well as immune responses (Cotter et al. 2003; Lindstedt et al. 2020) are sensitive to the pool of resources allocated to these different functions, they can be more easily detected under limited resources (King et al. 2011)—in this case, on the *P. rigida* host. Thus, to rigorously test the carotenoid trade‐off hypotheses due to host plant shifts, rearing experiments of white‐ and yellow‐bodied larvae on alternative host‐plant species are needed.

To conclude, our integrated analysis of color variation in *Neodiprion* sawflies provides a valuable point of comparison for other classic aposematic systems, such as *Heliconius* butterflies. Although both taxa exhibit striking among‐population variation in color attributable to major‐effect loci, our data indicate that the observed polytypisms likely evolved under very different evolutionary scenarios and selection pressures. We hypothesize that these differences ultimately stem from differences in the source of pigments and the role of color in mate choice. To evaluate these hypotheses more rigorously, comparable analyses of diverse aposematic taxa that vary in pigment sources (dietary vs. endogenous), life stages (immature vs. adult), and behavior (mobility and gregariousness in larval stage vs. adult stage) are needed (Willmott et al. 2011; Linnen et al. 2018; Galarza et al. 2019; Lindstedt et al. 2019). More generally, this kind of integrative study approach will give us a more comprehensive understanding of how organisms adapt to changes in their environment (Zaman et al. 2019) and how ecological selection during immature life stages impacts adaptation and speciation.

### AUTHOR CONTRIBUTIONS

CRL and CL conceived the original idea. CRL, CL, RKB, and MJ designed methodology and collected the data. CRL, CL, RKB, and SC analyzed the data. CRL and CL led the writing of the manuscript. All authors contributed critically to the drafts and writing and gave final approval for publication.

### CONFLICT OF INTEREST

The authors declare no conflict of interest.

### ETHICS STATEMENT FOR THE EXPERIMENTS WITH BIRDS

Predation experiments with wild great tits at Konnevesi Research Station (Jyväskylä, Finland) were carried out with permission from the Central Finland Centre for Economic Development, Transport and Environment (KESELY/1017/07.01/2010) and license from the National Animal Experiment Board (ESAVI‐2010‐087517Ym‐23).

### DATA ARCHIVING

All the datasets used in this study are available as source data files named and linked for each figure and table accordingly. Statistical models with the full list of parameters used are defined in the methods section and for some of the analyses as Supporting Information (referred in text as Fig. Sx or Table Sx). R‐codes and scripts (https://doi.org/10.5281/zenodo.5772251) included for the accepted final version of the manuscript together with the datasets (https://doi.org/10.5061/dryad.cz8w9gj4w) are openly available. Demultiplexed sequence files for all individuals will be available on the SRA (NCBI BioSample numbers: SAMN23893938‐SAMN23894002).

Associate Editor: Prof. Thomas Flatt

Handling Editor: Prof. Tracey Chapman

## Supporting information

Figure S1. Avoidance learning rates of birds towards white and yellow *Neodiprion lecontei* larvae were compared with light green‐black *Diprion pini* larvae with the similar defensive compounds (Photo Carita Lindstedt).

Figure S2. α score plot for DAPC.

Figure S3. Variance explained across PCs.

Figure S4. Luminance contrasts against different host plants and host plant parts.

Figure S5. Cluster number determined by model and non‐model based methods.

Table S1. Collection location, collection host, collection date, body color, and genetic cluster for all *Neodiprion lecontei* collected between 2001 and 2016.

Table S2. Human‐sorted white and yellow *Neodiprion lecontei* larvae reflected underlying differences in color. Similarly color of frozen white and yellow *N. lecontei* larvae recapitulate differences between fresh larvae.

Table S3. Sampling locations for all individuals included in population genomic analyses.

Table S4 – Sequences for adapters containing variable‐length barcodes from Burford Reiskind *et al*. (2016).

Table S5 – Sequences for PCR primers, including degenerate bases.

Table S6. Per‐individual sequence statistic summary.

Table S7. Random and fixed effects from the linear mixed model on *Neodiprion lecontei* and host plant (*P. virginiana* [VA], *P. echinata* [SL], and *P. rigida*) color traits.
