# Supplementary material for: The impact of life stage and pigment source on the evolution of novel warning signal traits
Source: Evolution. 2022 Feb 10;76(3):554–72. doi: 10.1111/evo.14443 (PMC9304160; doi:10.1111/evo.14443)

**Figure S1.** Avoidance learning rates of birds towards white and yellow *Neodiprion lecontei* larvae were compared with light green-black *Diprion pini* larvae with the similar defensive compounds (Photo Carita Lindstedt).


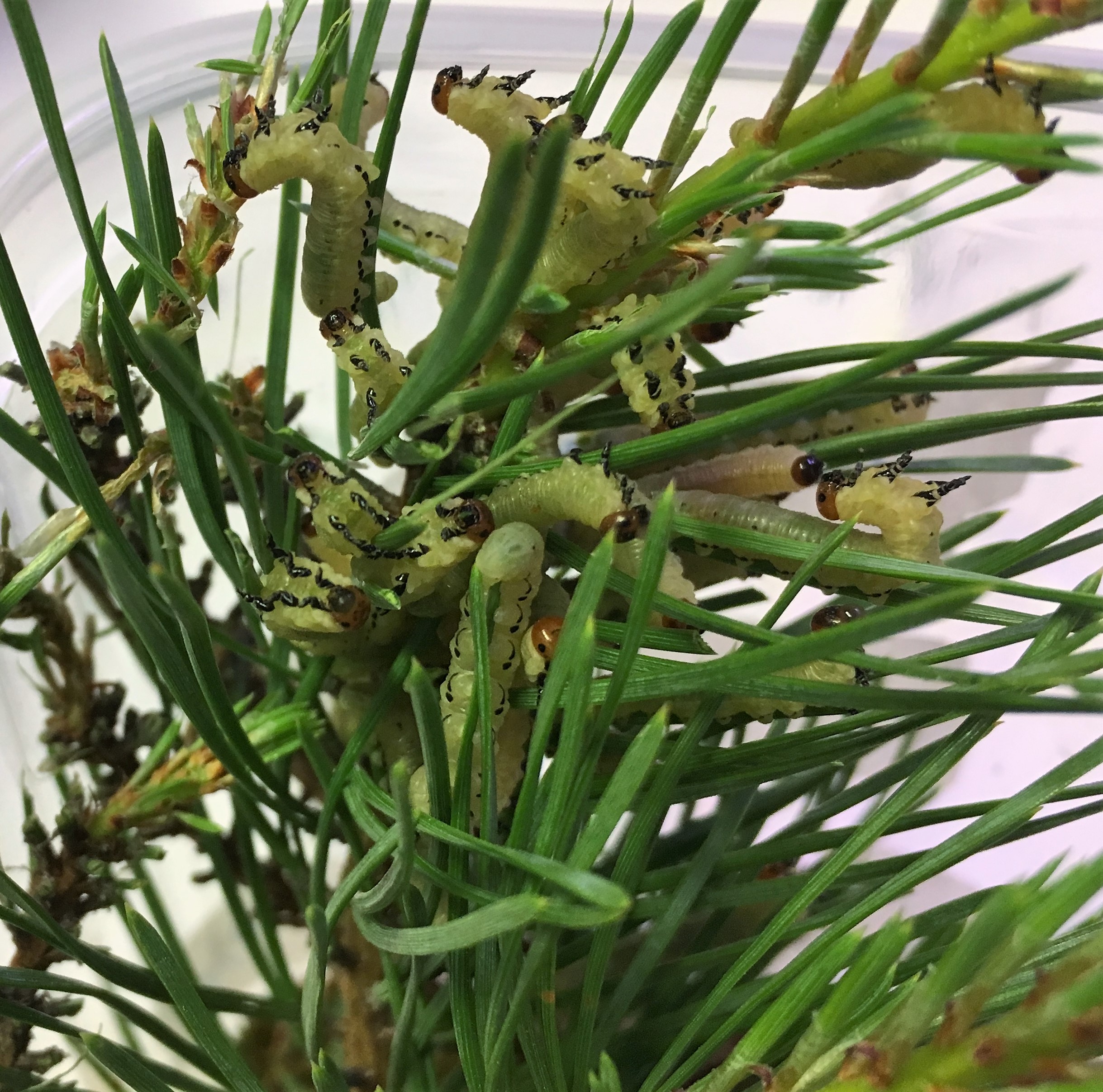

Supplement: Supplementary file 1 — Figure S1. Avoidance learning rates of birds towards white and yellow Neodiprion lecontei larvae were compared with light green‐black Diprion pini larvae with the similar defensive compounds (Photo Carita Lindstedt). [file EVO-76-554-s003.docx]
