# Supplementary figures and images for: The impact of life stage and pigment source on the evolution of novel warning signal traits
Source: Evolution. 2022 Feb 10;76(3):554–72. doi: 10.1111/evo.14443 (PMC9304160; doi:10.1111/evo.14443)

**Figure S2. α score plot for DAPC.** The optimal number of PCs to retain (1) is indicated by a red circle.

**
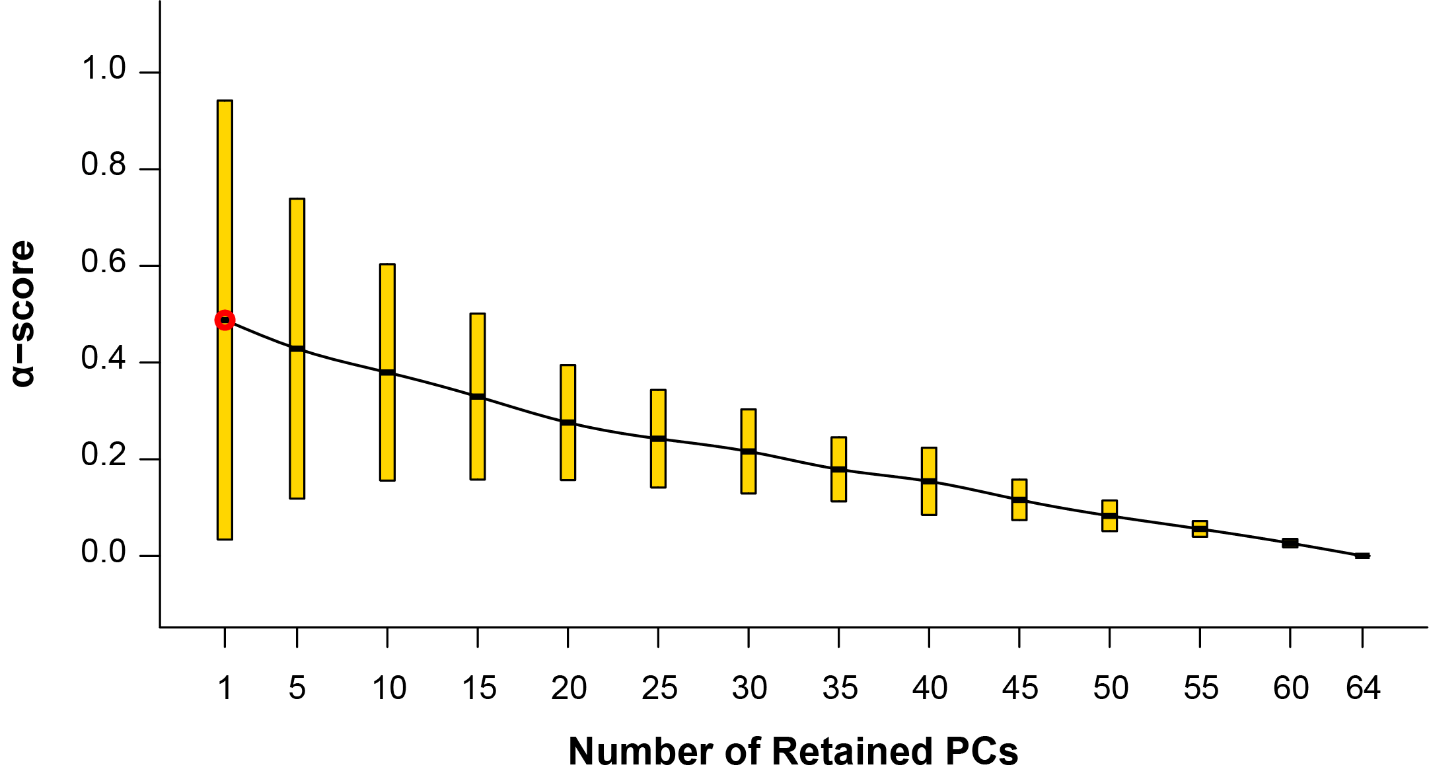
**

Supplement: Supplementary file 2 — Figure S2. α score plot for DAPC. [file EVO-76-554-s007.docx]

**Figure S3. Variance explained across PCs.**


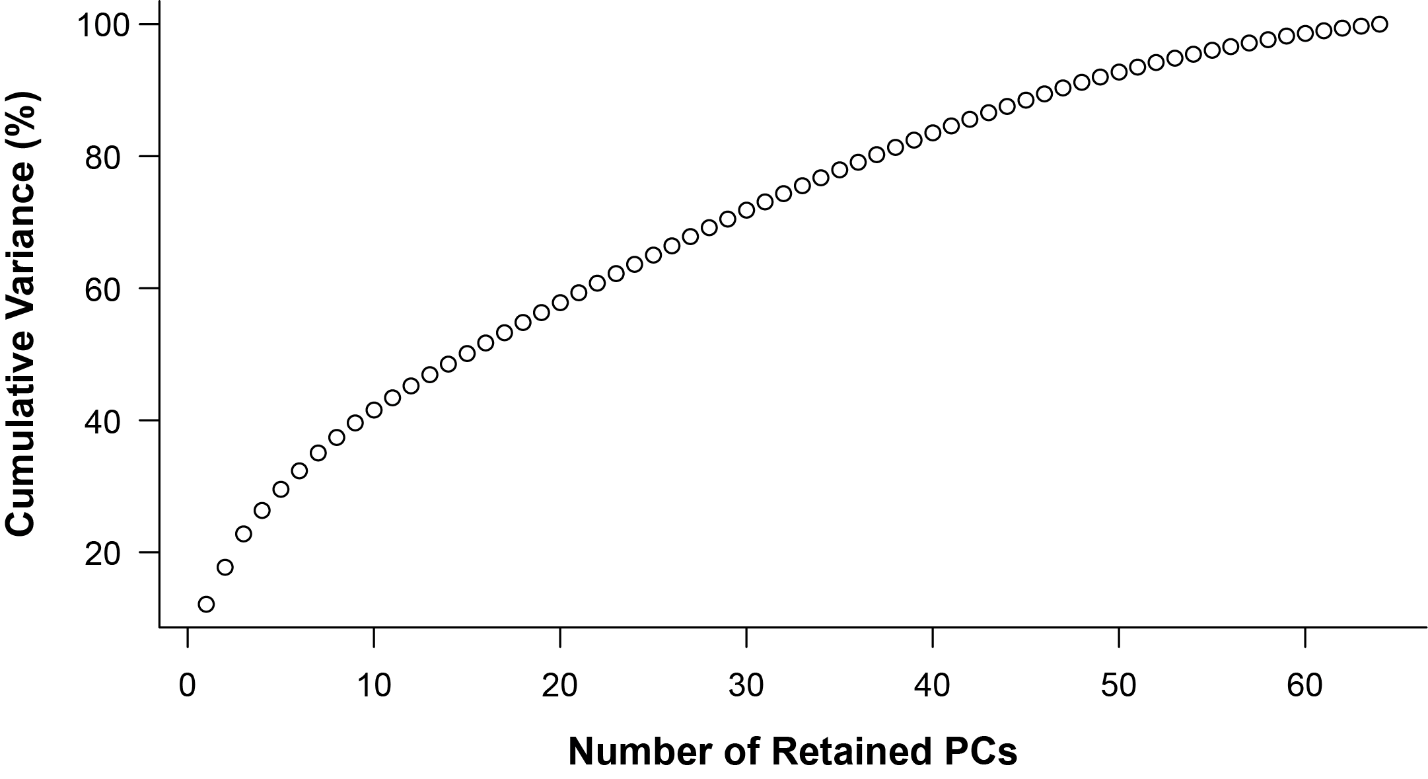

Supplement: Supplementary file 3 — Figure S3. Variance explained across PCs. [file EVO-76-554-s005.docx]
