# Supplementary material for: The impact of life stage and pigment source on the evolution of novel warning signal traits
Source: Evolution. 2022 Feb 10;76(3):554–72. doi: 10.1111/evo.14443 (PMC9304160; doi:10.1111/evo.14443)

**Figure S4.** Luminance contrasts against different host plants and host plant parts. For reference, JND values for prey/background combinations that are <1 are indistinguishable, values between <1 and 3 are hard to distinguish unless under optimal conditions, and values > 5 are easy to tell apart under most conditions (Vorobyev and Osorio 1998). Dashed lines show the threshold value for JND=5 above which objects should appear clearly conspicuous for blue tits (*Cyanistes caeruleus*). Error bars are 95% confidence intervals.


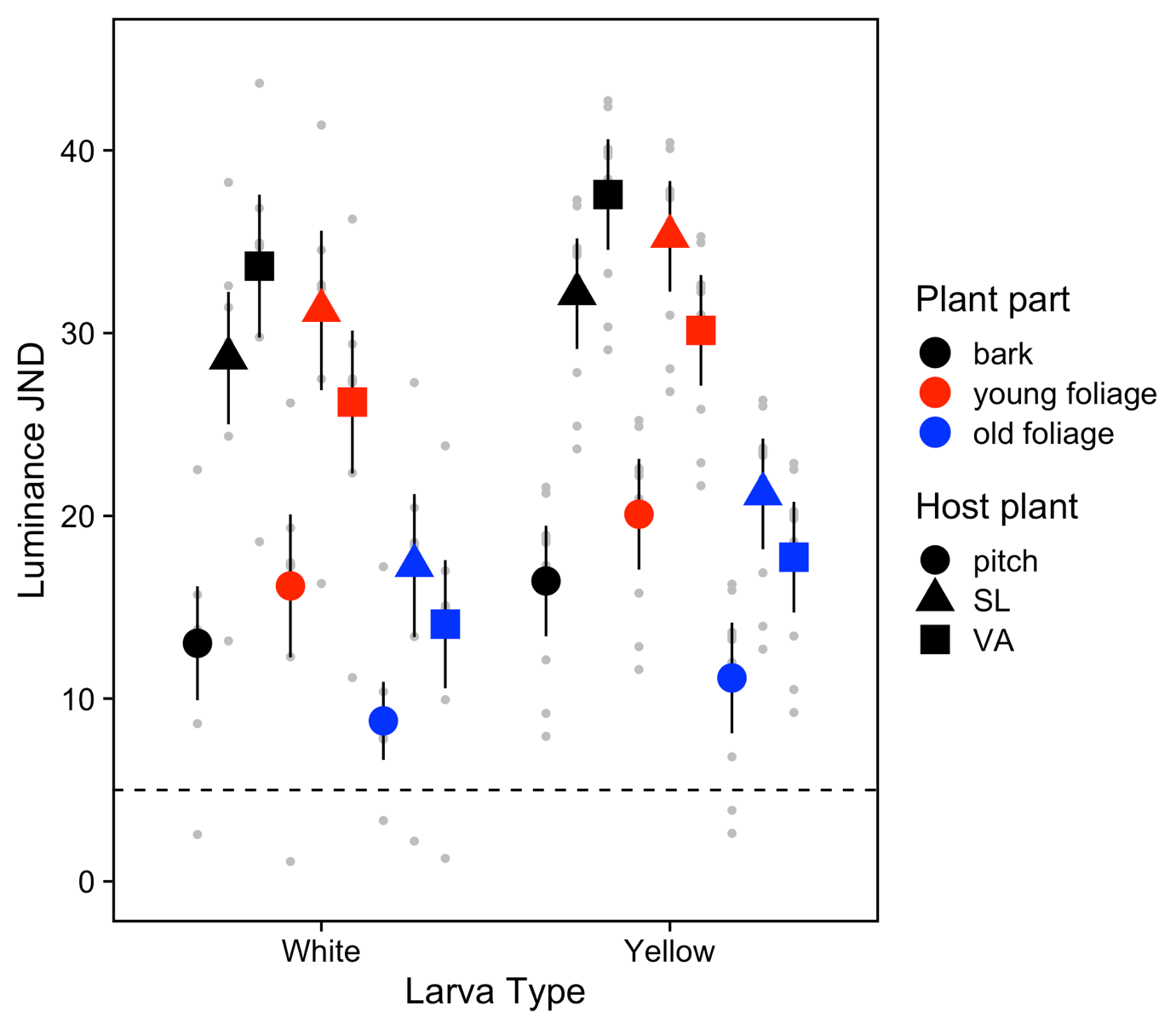

Supplement: Supplementary file 4 — Figure S4. Luminance contrasts against different host plants and host plant parts. [file EVO-76-554-s010.docx]
