# Supplementary material for: The impact of life stage and pigment source on the evolution of novel warning signal traits
Source: Evolution. 2022 Feb 10;76(3):554–72. doi: 10.1111/evo.14443 (PMC9304160; doi:10.1111/evo.14443)
Supplement: Supplementary file 5 — Figure S5. Cluster number determined by model and non‐model based methods. [file EVO-76-554-s002.docx]

**Figure S5. Cluster number determined by model and non-model based methods.** A *K* of 2 is suggested using both admixture’s 5-fold CV error score (A) and *adegenet*’s BIC score (B).

**
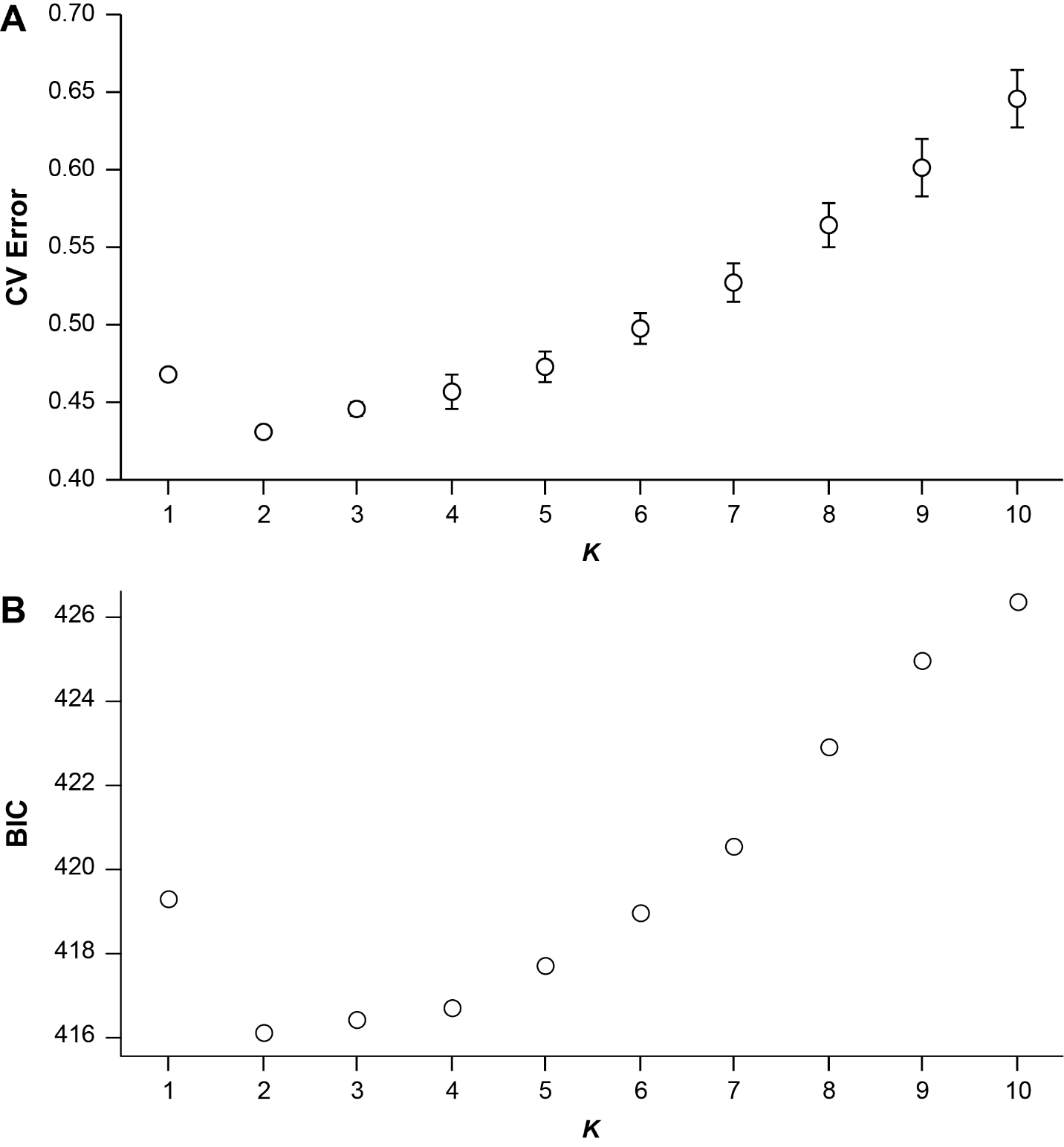
**
