# Supplementary material for: The impact of life stage and pigment source on the evolution of novel warning signal traits
Source: Evolution. 2022 Feb 10;76(3):554–72. doi: 10.1111/evo.14443 (PMC9304160; doi:10.1111/evo.14443)
Supplement: Supplementary file 6 — Table S1. Collection location, collection host, collection date, body color, and genetic cluster for all Neodiprion lecontei collected between 2001 and 2016. [file EVO-76-554-s011.pdf]

Table S1. Collection location, collection host, collection date, body color, and genetic cluster for all *N. lecontei* collected between 2001 and 2016.

Specimen ID	Latitude	Longitude	Larval Color	Genetic Cluster	City, State/Province	Collection Host	Collection Date
001-01	42.229	-71.523	YELLOW	CENTRAL	Hopkinton, MA	*P. banksiana*	19-Jul-2001
002-01	42.229	-71.523	YELLOW	CENTRAL	Hopkinton, MA	*P. banksiana*	19-Jul-2001
003-01	42.229	-71.523	YELLOW	CENTRAL	Hopkinton, MA	*P. banksiana*	19-Jul-2001
017-01	44.544	-73.215	YELLOW	CENTRAL	Malletts Bay, VT	*P. resinosa*	3-Aug-2001
018-01	44.544	-73.215	YELLOW	CENTRAL	Malletts Bay, VT	*P. resinosa*	3-Aug-2001
025-0263	44.137	-75.639	YELLOW	NORTH	Kemptville, ON	*P. resinosa*	20-Jul-2002
025-0309	44.395	-77.205	YELLOW	NORTH	Tweed, ON	*P. resinosa*	7-Aug-2002
025-0312	44.730	-79.169	YELLOW	NORTH	Orillia, ON	*P. resinosa*	20-Aug-2002
025-0335	46.017	-77.450	YELLOW	NORTH	Chalk River, ON	*P. resinosa*	24-Aug-2002
025-0339B	46.383	-82.650	YELLOW	NORTH	Elliot Lake, ON	*P. resinosa*	15-Aug-2002
075-04	28.096	-81.275	YELLOW	SOUTH	Canoe Creek, FL	*P. elliottii*	10-Jul-2004
076-04	28.096	-81.275	YELLOW	SOUTH	Canoe Creek, FL	*P. elliottii*	10-Jul-2004
077-04	26.923	-81.336	YELLOW	SOUTH	Palmdale, FL	*P. elliottii*	11-Jul-2004
078-04	26.923	-81.336	YELLOW	SOUTH	Palmdale, FL	*P. elliottii*	11-Jul-2004
079-04	26.923	-81.336	YELLOW	SOUTH	Palmdale, FL	*P. elliottii*	11-Jul-2004
085-04	29.718	-82.457	YELLOW	SOUTH	Gainesville, FL	*P. palustris*	12-Jul-2004
086-04	29.718	-82.457	YELLOW	SOUTH	Gainesville, FL	*P. palustris*	12-Jul-2004
087-04	29.748	-82.477	YELLOW	SOUTH	Gainesville, FL	*P. taeda*	13-Jul-2004
088-04	29.748	-82.477	YELLOW	SOUTH	Gainesville, FL	*P. palustris*	13-Jul-2004
096-04	31.498	-84.593	YELLOW	SOUTH	Morgan, GA	*P. taeda*	14-Jul-2004
097-04	31.498	-84.593	YELLOW	SOUTH	Morgan, GA	*P. glabra*	14-Jul-2004
102-04	31.555	-83.989	YELLOW	SOUTH	Sylvester, GA	*P. elliottii*	15-Jul-2004
106-04	32.074	-83.761	YELLOW	SOUTH	Vienna, GA	*P. elliottii*	17-Jul-2004
116-04	36.039	-85.109	YELLOW	CENTRAL	Crossville, TN	*P. virginiana*	19-Jul-2004
125-02	43.115	-71.100	YELLOW	CENTRAL	Nottingham, NH	*P. sylvestris*	21-Jul-2002

129-02	43.115	-71.100	YELLOW	CENTRAL	Nottingham, NH	*P. sylvestris*	21-Jul-2002
132-04	38.716	-76.064	WHITE	CENTRAL	Trappe, MD	*P. virginiana*	23-Jul-2004
133-04	38.716	-76.064	WHITE	CENTRAL	Trappe, MD	*P. taeda*	23-Jul-2004
145-04	43.781	-71.170	YELLOW	CENTRAL	Ossippee, NH	*P. rigida*	27-Jul-2004
164-02	45.073	-77.710	YELLOW	NORTH	Bancroft, ON	*P. resinosa*	9-Aug-2002
168-02	44.856	-77.859	YELLOW	NORTH	Apsley, ON	*P. banksiana*	9-Aug-2002
168-04	43.685	-71.117	YELLOW	CENTRAL	Ossippee, NH	*P. rigida*	30-Jul-2004
171-02	44.730	-79.169	YELLOW	NORTH	Sebrite, ON	*P. resinosa*	10-Aug-2002
173-02	44.730	-79.169	YELLOW	NORTH	Sebrite, ON	*P. resinosa*	10-Aug-2002
174-02	44.730	-79.169	YELLOW	NORTH	Sebrite, ON	*P. resinosa*	10-Aug-2002
174-03A	29.680	-83.257	YELLOW	SOUTH	Dixie Co, FL	*P. taeda*	23-Nov-2003
175-02	44.730	-79.169	YELLOW	NORTH	Sebrite, ON	*P. resinosa*	10-Aug-2002
176-02	44.730	-79.169	YELLOW	NORTH	Sebrite, ON	*P. resinosa*	10-Aug-2002
177-02	44.730	-79.169	YELLOW	NORTH	Sebrite, ON	*P. resinosa*	10-Aug-2002
178-02	44.730	-79.169	YELLOW	NORTH	Sebrite, ON	*P. resinosa*	10-Aug-2002
178-03	30.428	-85.603	YELLOW	SOUTH	Crystal Lake, FL	*P. palustris*	24-Nov-2003
180-03	28.787	-81.982	YELLOW	SOUTH	Lake Co, FL	*P. taeda*	25-Nov-2003
183-03	26.871	-81.521	YELLOW	SOUTH	Glades Co, FL	*P. palustris*	26-Nov-2003
185-03	26.923	-81.337	YELLOW	SOUTH	Palmdale, FL	*P. elliottii*	26-Nov-2003
188-04	44.759	-91.457	YELLOW	NORTH	Eau Claire, WI	*P. banksiana*	15-Aug-2004
196-04	43.912	-90.866	YELLOW	NORTH	Sparta, WI	*P. banksiana*	15-Aug-2004
207-04	45.975	-90.496	YELLOW	NORTH	Park Falls, WI	*P. banksiana*	17-Aug-2004
339-02	46.348	-79.334	YELLOW	NORTH	North Bay, ON	*P. banksiana*	19-Aug-2002
342-02	46.395	-79.244	YELLOW	NORTH	North Bay, ON	*P. banksiana*	19-Aug-2002
343-02	46.395	-79.244	YELLOW	NORTH	North Bay, ON	*P. banksiana*	19-Aug-2002
344-02	46.395	-79.244	YELLOW	NORTH	North Bay, ON	*P. resinosa*	19-Aug-2002
345-02	46.395	-79.244	YELLOW	NORTH	North Bay, ON	*P. resinosa*	19-Aug-2002
349-02	46.378	-78.867	YELLOW	NORTH	Mattawan, ON	*P. resinosa*	19-Aug-2002
352-02	46.378	-78.867	YELLOW	NORTH	Mattawan, ON	*P. resinosa*	19-Aug-2002

372-02	41.874	-70.652	WHITE	CENTRAL	Plymouth, MA	*P. rigida*	25-Sep-2002
379-02	41.866	-70.658	WHITE	CENTRAL	Plymouth, MA	*P. rigida*	25-Sep-2002
CAN002	44.736	-79.161	YELLOW	NORTH	Lindsay, ON	*P. banksiana*	27-Jul-2014
CAN003	44.736	-79.161	YELLOW	NORTH	Lindsay, ON	*P. banksiana*	27-Jul-2014
CAN004	44.736	-79.161	YELLOW	NORTH	Lindsay, ON	*P. banksiana*	27-Jul-2014
CAN005	44.736	-79.161	YELLOW	NORTH	Lindsay, ON	*P. resinosa*	27-Jul-2014
CAN006	44.732	-79.169	YELLOW	NORTH	Lindsay, ON	*P. resinosa*	27-Jul-2014
CAN007	44.863	-78.113	YELLOW	NORTH	Harcourt, ON	*P. resinosa*	27-Jul-2014
CAN008	44.863	-78.113	YELLOW	NORTH	Harcourt, ON	*P. resinosa*	27-Jul-2014
CAN009	44.863	-78.113	YELLOW	NORTH	Harcourt, ON	*P. strobus*	27-Jul-2014
CAN011	45.316	-77.764	YELLOW	NORTH	Combermere, ON	*P. strobus*	28-Jul-2014
CAN012	45.316	-77.764	YELLOW	NORTH	Combermere, ON	*P. strobus*	28-Jul-2014
CAN013	45.316	-77.764	YELLOW	NORTH	Combermere, ON	*P. resinosa*	28-Jul-2014
CAN014	45.316	-77.764	YELLOW	NORTH	Combermere, ON	*P. resinosa*	28-Jul-2014
CAN015	45.316	-77.764	YELLOW	NORTH	Combermere, ON	*P. resinosa*	28-Jul-2014
CAN016	45.316	-77.764	YELLOW	NORTH	Combermere, ON	*P. resinosa*	28-Jul-2014
CAN017	45.316	-77.764	YELLOW	NORTH	Combermere, ON	*P. resinosa*	28-Jul-2014
CAN018	45.316	-77.764	YELLOW	NORTH	Combermere, ON	*P. resinosa*	28-Jul-2014
CAN019	45.316	-77.764	YELLOW	NORTH	Combermere, ON	*P. resinosa*	28-Jul-2014
CAN020	45.316	-77.764	YELLOW	NORTH	Combermere, ON	*P. resinosa*	28-Jul-2014
CAN021	45.483	-77.675	YELLOW	NORTH	Barry's Bay, ON	*P. resinosa*	28-Jul-2014
CAN022	45.483	-77.675	YELLOW	NORTH	Barry's Bay, ON	*P. resinosa*	28-Jul-2014
CAN023	45.483	-77.675	YELLOW	NORTH	Barry's Bay, ON	*P. resinosa*	28-Jul-2014
CAN024	45.483	-77.675	YELLOW	NORTH	Barry's Bay, ON	*P. resinosa*	28-Jul-2014
CAN025	45.483	-77.675	YELLOW	NORTH	Barry's Bay, ON	*P. resinosa*	28-Jul-2014
CAN026	45.486	-77.672	YELLOW	NORTH	Barry's Bay, ON	*P. resinosa*	28-Jul-2014
CAN027	45.486	-77.672	YELLOW	NORTH	Barry's Bay, ON	*P. resinosa*	28-Jul-2014
CAN028	45.486	-77.672	YELLOW	NORTH	Barry's Bay, ON	*P. resinosa*	28-Jul-2014
CAN029	45.486	-77.672	YELLOW	NORTH	Barry's Bay, ON	*P. resinosa*	28-Jul-2014

CAN030	45.486	-77.672	YELLOW	NORTH	Barry's Bay, ON	*P. resinosa*	28-Jul-2014
CAN031	45.486	-77.672	YELLOW	NORTH	Barry's Bay, ON	*P. resinosa*	28-Jul-2014
CAN032	45.486	-77.672	YELLOW	NORTH	Barry's Bay, ON	*P. resinosa*	28-Jul-2014
CAN033	45.486	-77.672	YELLOW	NORTH	Barry's Bay, ON	*P. resinosa*	28-Jul-2014
CAN034	45.486	-77.672	YELLOW	NORTH	Barry's Bay, ON	*P. resinosa*	28-Jul-2014
CAN035	45.486	-77.672	YELLOW	NORTH	Barry's Bay, ON	*P. resinosa*	28-Jul-2014
CAN036	45.798	-77.192	YELLOW	NORTH	Laurentian Valley, ON	*P. resinosa*	29-Jul-2014
CAN037a	45.834	-77.237	YELLOW	NORTH	Laurentian Valley, ON	*P. resinosa*	29-Jul-2014
CAN038	45.834	-77.237	YELLOW	NORTH	Laurentian Valley, ON	*P. resinosa*	29-Jul-2014
CAN039	45.834	-77.237	YELLOW	NORTH	Laurentian Valley, ON	*P. resinosa*	29-Jul-2014
CAN040	46.288	-78.812	YELLOW	NORTH	Papineau-Cameron, ON	*P. resinosa*	29-Jul-2014
CAN041	46.370	-81.384	YELLOW	NORTH	Greater Sudbury, ON	*P. resinosa*	30-Jul-2014
CAN042	46.288	-81.793	YELLOW	NORTH	Baldwin, ON	*P. resinosa*	30-Jul-2014
CAN043	44.601	-84.712	YELLOW	NORTH	Grayling, MI	*P. banksiana*	31-Jul-2014
CAN044	44.601	-84.712	YELLOW	NORTH	Grayling, MI	*P. banksiana*	31-Jul-2014
CAN045	44.601	-84.712	YELLOW	NORTH	Grayling, MI	*P. banksiana*	31-Jul-2014
CAN046	44.601	-84.712	YELLOW	NORTH	Grayling, MI	*P. banksiana*	31-Jul-2014
CAN047	44.601	-84.712	YELLOW	NORTH	Grayling, MI	*P. banksiana*	31-Jul-2014
CAN048	44.657	-84.696	YELLOW	NORTH	Grayling, MI	*P. resinosa*	1-Aug-2014
CAN049	44.657	-84.696	YELLOW	NORTH	Grayling, MI	*P. resinosa*	1-Aug-2014
CAN050	44.657	-84.696	YELLOW	NORTH	Grayling, MI	*P. resinosa*	1-Aug-2014
CAN051	44.657	-84.696	YELLOW	NORTH	Grayling, MI	*P. sylvestris*	1-Aug-2014
CAN052	44.657	-84.696	YELLOW	NORTH	Grayling, MI	*P. strobus*	1-Aug-2014
CAN053	44.657	-84.696	YELLOW	NORTH	Grayling, MI	*P. strobus*	1-Aug-2014
CAN054	44.657	-84.696	YELLOW	NORTH	Grayling, MI	*P. banksiana*	1-Aug-2014
CAN055	44.657	-84.696	YELLOW	NORTH	Grayling, MI	*P. banksiana*	1-Aug-2014
CAN056	44.657	-84.696	YELLOW	NORTH	Grayling, MI	*P. banksiana*	1-Aug-2014
CAN057	44.122	-85.471	YELLOW	NORTH	Tustin, MI	*P. banksiana*	1-Aug-2014
CAN059	43.759	-85.741	YELLOW	NORTH	Bitely, MI	*P. sylvestris*	1-Aug-2014

CAN060	43.789	-85.740	YELLOW	NORTH	Bitely, MI	*P. sylvestris*	1-Aug-2014
CAN061a	43.789	-85.740	YELLOW	NORTH	Bitely, MI	*P. banksiana*	1-Aug-2014
CAN062	43.769	-85.741	YELLOW	NORTH	Bitely, MI	*P. banksiana*	1-Aug-2014
CAN063	46.214	-83.099	YELLOW	NORTH	Blind River, ON	*P. resinosa*	13-Aug-2014
CAN064	46.220	-83.108	YELLOW	NORTH	Blind River, ON	*P. resinosa*	15-Aug-2014
CAN065	46.220	-83.108	YELLOW	NORTH	Blind River, ON	*P. resinosa*	15-Aug-2014
CAN066	46.220	-83.108	YELLOW	NORTH	Blind River, ON	*P. resinosa*	15-Aug-2014
CAN067	46.220	-83.108	YELLOW	NORTH	Blind River, ON	*P. resinosa*	15-Aug-2014
CAN068	46.220	-83.108	YELLOW	NORTH	Blind River, ON	*P. resinosa*	15-Aug-2014
CAN069	46.214	-83.099	YELLOW	NORTH	Blind River, ON	*P. resinosa*	15-Aug-2014
CAN070	46.214	-83.099	YELLOW	NORTH	Blind River, ON	*P. resinosa*	15-Aug-2014
CAN071	46.214	-83.099	YELLOW	NORTH	Blind River, ON	*P. resinosa*	15-Aug-2014
CAN072	46.214	-83.099	YELLOW	NORTH	Blind River, ON	*P. resinosa*	15-Aug-2014
CAN073	46.214	-83.099	YELLOW	NORTH	Blind River, ON	*P. resinosa*	15-Aug-2014
CAN074	46.214	-83.099	YELLOW	NORTH	Blind River, ON	*P. resinosa*	15-Aug-2014
CAN075	46.201	-82.358	YELLOW	NORTH	Spanish, ON	*P. resinosa*	15-Aug-2014
CAN076	46.201	-82.358	YELLOW	NORTH	Spanish, ON	*P. resinosa*	15-Aug-2014
CAN077	46.212	-82.081	YELLOW	NORTH	Sables-Spanish River, ON	*P. banksiana*	15-Aug-2014
CAN078	46.221	-82.024	YELLOW	NORTH	Sables-Spanish River, ON	*P. resinosa*	15-Aug-2014
CAN079	46.221	-82.024	YELLOW	NORTH	Sables-Spanish River, ON	*P. sylvestris*	15-Aug-2014
CAN080	46.569	-81.232	YELLOW	NORTH	Chelmsford, ON	*P. banksiana*	16-Aug-2014
CAN082	46.587	-81.382	YELLOW	NORTH	Dowling, ON	*P. resinosa*	16-Aug-2014
CAN083	46.732	-81.589	YELLOW	NORTH	Cartier, ON	*P. resinosa*	16-Aug-2014
CAN084	46.732	-81.589	YELLOW	NORTH	Cartier, ON	*P. banksiana*	16-Aug-2014
CAN090	47.032	-83.152	YELLOW	NORTH	Algmoa, Unorganized, North Part, ON	*P. banksiana*	17-Aug-2014
CAN092	46.719	-83.425	YELLOW	NORTH	Algmoa, Unorganized, ON	*P. resinosa*	17-Aug-2014
CAN093	46.719	-83.425	YELLOW	NORTH	Algmoa, Unorganized, ON	*P. resinosa*	17-Aug-2014
CAN094	46.719	-83.425	YELLOW	NORTH	Algmoa, Unorganized, ON	*P. resinosa*	17-Aug-2014

CAN095	46.719	-83.425	YELLOW	NORTH	Algmoa, Unorganized, ON	*P. strobus*	17-Aug-2014
CAN096	46.719	-83.425	YELLOW	NORTH	Algmoa, Unorganized, ON	*P. strobus*	17-Aug-2014
CAN097	46.719	-83.425	YELLOW	NORTH	Algmoa, Unorganized, ON	*P. strobus*	17-Aug-2014
CAN098	44.335	-90.730	YELLOW	NORTH	Black River Falls, WI	*P. banksiana/P. strobus*	21-Aug-2014
CAN099	43.799	-85.736	YELLOW	NORTH	Bitely, MI	*P. resinosa*	11-Sep-2014
CAN100	44.704	-84.906	YELLOW	NORTH	Grayling, MI	*P. banksiana*	11-Sep-2014
CAN101	45.924	-86.302	YELLOW	NORTH	Manistique, MI	*P. banksiana*	12-Sep-2014
CAN102	45.892	-86.520	YELLOW	NORTH	Manistique, MI	*P. banksiana*	12-Sep-2014
CAN103	44.348	-90.346	YELLOW	NORTH	City Point, WI	*P. banksiana*	13-Sep-2014
CAN104	44.341	-90.411	YELLOW	NORTH	Pittsville, WI	*P. banksiana*	13-Sep-2014
CAN105	44.341	-90.411	YELLOW	NORTH	Pittsville, WI	*P. banksiana*	13-Sep-2014
CAN106	44.341	-90.411	YELLOW	NORTH	Pittsville, WI	*P. banksiana*	13-Sep-2014
CAN107	44.130	-90.393	YELLOW	NORTH	Warrens, WI	*P. banksiana*	13-Sep-2014
CAN108	44.130	-90.393	YELLOW	NORTH	Warrens, WI	*P. banksiana*	13-Sep-2014
CAN109	44.199	-90.136	YELLOW	NORTH	Necedah, WI	*P. banksiana*	13-Sep-2014
CAN111	44.036	-90.082	YELLOW	NORTH	Necedah, WI	*P. banksiana*	13-Sep-2014
LL002	38.014	-84.504	YELLOW	CENTRAL	Lexington, KY	*P. echinata*	13-Jun-2012
LL003	38.014	-84.504	YELLOW	CENTRAL	Lexington, KY	*P. echinata*	13-Jun-2012
LL004	38.014	-84.504	YELLOW	CENTRAL	Lexington, KY	*P. echinata*	13-Jun-2012
LL005	38.014	-84.504	YELLOW	CENTRAL	Lexington, KY	*P. rigida*	15-Jun-2012
LL006	38.014	-84.504	YELLOW	CENTRAL	Lexington, KY	*P. rigida*	15-Jun-2012
LL007	38.014	-84.504	YELLOW	CENTRAL	Lexington, KY	*P. virginiana*	15-Jun-2012
LL009	35.929	-84.914	YELLOW	CENTRAL	Crossville, TN	*P. virginiana*	11-Jul-2013
LL010	36.928	-84.619	YELLOW	CENTRAL	Bronston, KY	*P. virginiana*	14-Jul-2013
LL011	37.071	-84.211	YELLOW	CENTRAL	London, KY	*P. echinata*	16-Jul-2013
LL012	37.071	-84.211	YELLOW	CENTRAL	London, KY	*P. echinata*	16-Jul-2013
LL013	37.071	-84.211	YELLOW	CENTRAL	London, KY	*P. echinata*	16-Jul-2013
LL014	37.071	-84.211	YELLOW	CENTRAL	London, KY	*P. echinata*	16-Jul-2013

LL015	37.071	-84.211	YELLOW	CENTRAL	London, KY	*P. echinata*	16-Jul-2013
LL017	38.014	-84.504	YELLOW	CENTRAL	Lexington, KY	*P. echinata*	18-Jul-2013
LL030	34.115	-79.940	YELLOW	SOUTH	Florence county, SC	*P. palustris*	5-Aug-2013
LL031	40.550	-74.431	MIXED	CENTRAL	Piscataway, NJ	*P. sylvestris*	14-Aug-2013
LL032	46.183	-82.948	YELLOW	NORTH	Blind River, ON	*P. resinosa*	15-Aug-2013
LL033	46.183	-82.948	YELLOW	NORTH	Blind River, ON	*NA*	15-Aug-2013
LL034	44.939	-75.671	YELLOW	NORTH	Oxford Mills, ON	*P. resinosa*	16-Aug-2013
LL035	46.296	-83.550	YELLOW	NORTH	Little Rapids, ON	*P. resinosa*	18-Aug-2013
LL036	46.422	-83.375	YELLOW	NORTH	Whamcliffe, ON	*P. resinosa*	18-Aug-2013
LL037	46.207	-83.060	YELLOW	NORTH	Mississaugi River, ON	*P. resinosa*	19-Aug-2013
LL038	46.183	-82.948	YELLOW	NORTH	Blind River, ON	*P. resinosa*	15-Aug-2013
LL039	46.791	-84.030	YELLOW	NORTH	Searchmont, ON	*P. banksiana*	22-Aug-2013
LL040	46.791	-84.030	YELLOW	NORTH	Searchmont, ON	*P. banksiana*	22-Aug-2013
LL041	46.372	-82.606	YELLOW	NORTH	Elliot Lake, ON	*P. resinosa*	23-Aug-2013
LL042	37.249	-77.725	WHITE	CENTRAL	Amelia, VA	*P. echinata*	18-Sep-2013
LL045	29.474	-82.861	YELLOW	SOUTH	Chiefland, FL	*P. palustris*	7-Apr-2014
LL047	38.014	-84.504	YELLOW	CENTRAL	Lexington, KY	*P. echinata*	9-Jun-2014
LL048	38.014	-84.504	YELLOW	CENTRAL	Lexington, KY	*P. echinata*	9-Jun-2014
LL049	38.014	-84.504	YELLOW	CENTRAL	Lexington, KY	*P. echinata*	9-Jun-2014
LL050	38.014	-84.504	YELLOW	CENTRAL	Lexington, KY	*P. echinata*	9-Jun-2014
LL051	38.014	-84.504	YELLOW	CENTRAL	Lexington, KY	*P. echinata*	9-Jun-2014
LL052	38.014	-84.504	YELLOW	CENTRAL	Lexington, KY	*P. virginiana*	9-Jun-2014
LL053	38.014	-84.504	YELLOW	CENTRAL	Lexington, KY	*P. virginiana*	9-Jun-2014
LL054	38.014	-84.504	YELLOW	CENTRAL	Lexington, KY	*P. virginiana*	9-Jun-2014
LL055	38.014	-84.504	YELLOW	CENTRAL	Lexington, KY	*P. virginiana*	9-Jun-2014
LL056	38.014	-84.504	YELLOW	CENTRAL	Lexington, KY	*P. rigida*	9-Jun-2014
LL057	38.014	-84.504	YELLOW	CENTRAL	Lexington, KY	*P. rigida*	9-Jun-2014
LL058	38.014	-84.504	YELLOW	CENTRAL	Lexington, KY	*P. virginiana*	19-Jun-2014
LL059	38.014	-84.504	YELLOW	CENTRAL	Lexington, KY	*P. virginiana*	19-Jun-2014

LL060	38.014	-84.504	YELLOW	CENTRAL	Lexington, KY	*P. virginiana*	19-Jun-2014
LL061	38.014	-84.504	YELLOW	CENTRAL	Lexington, KY	*P. virginiana*	19-Jun-2014
LL062	38.014	-84.504	YELLOW	CENTRAL	Lexington, KY	*P. echinata*	19-Jun-2014
LL063	38.014	-84.504	YELLOW	CENTRAL	Lexington, KY	*P. echinata*	19-Jun-2014
LL064	38.014	-84.504	YELLOW	CENTRAL	Lexington, KY	*P. rigida*	19-Jun-2014
LL065	38.014	-84.504	YELLOW	CENTRAL	Lexington, KY	*P. rigida*	19-Jun-2014
LL066	38.014	-84.504	YELLOW	CENTRAL	Lexington, KY	*P. rigida*	19-Jun-2014
LL067	38.014	-84.504	YELLOW	CENTRAL	Lexington, KY	*P. rigida*	19-Jun-2014
LL068	35.782	-78.640	YELLOW	NORTH	Raleigh, NC	*P. palustris*	25-Jun-2014
LL069	38.014	-84.504	YELLOW	CENTRAL	Lexington, KY	*P. virginiana*	27-Jun-2014
LL070	38.014	-84.504	YELLOW	CENTRAL	Lexington, KY	*P. rigida*	27-Jun-2014
LL071	38.014	-84.504	YELLOW	CENTRAL	Lexington, KY	*P. echinata*	27-Jun-2014
LL072	38.014	-84.504	YELLOW	CENTRAL	Lexington, KY	*P. virginiana*	27-Jun-2014
LL073	38.014	-84.504	YELLOW	CENTRAL	Lexington, KY	*P. echinata*	27-Jun-2014
LL074	38.014	-84.504	YELLOW	CENTRAL	Lexington, KY	*P. echinata*	30-Jun-2014
LL075	38.014	-84.504	YELLOW	CENTRAL	Lexington, KY	*P. rigida*	30-Jun-2014
LL076	38.014	-84.504	YELLOW	CENTRAL	Lexington, KY	*P. virginiana*	30-Jun-2014
LL077	38.014	-84.504	YELLOW	CENTRAL	Lexington, KY	*P. virginiana*	30-Jun-2014
LL078	38.014	-84.504	YELLOW	CENTRAL	Lexington, KY	*P. rigida*	30-Jun-2014
LL081	38.014	-84.504	YELLOW	CENTRAL	Lexington, KY	*P. rigida*	24-Jul-2014
LL082	41.645	-70.230	WHITE	CENTRAL	West Yarmouth, MA	*P. rigida*	5-Aug-2014
LL083	41.645	-70.230	WHITE	CENTRAL	West Yarmouth, MA	*P. rigida*	5-Aug-2014
LL084	41.645	-70.230	WHITE	CENTRAL	West Yarmouth, MA	*P. rigida*	5-Aug-2014
LL086	38.014	-84.504	YELLOW	CENTRAL	Lexington, KY	*P. echinata*	28-Aug-2014
LL087	38.014	-84.504	YELLOW	CENTRAL	Lexington, KY	*P. echinata*	28-Aug-2014
LL088	38.014	-84.504	YELLOW	CENTRAL	Lexington, KY	*P. echinata*	28-Aug-2014
LL089	38.014	-84.504	YELLOW	CENTRAL	Lexington, KY	*P. echinata*	28-Aug-2014
LL090	38.014	-84.504	YELLOW	CENTRAL	Lexington, KY	*P. echinata*	28-Aug-2014
LL091	38.014	-84.504	YELLOW	CENTRAL	Lexington, KY	*P. echinata*	28-Aug-2014

LL092	38.014	-84.504	YELLOW	CENTRAL	Lexington, KY	*P. virginiana*	28-Aug-2014
LL093	38.014	-84.504	YELLOW	CENTRAL	Lexington, KY	*P. virginiana*	28-Aug-2014
LL094	38.014	-84.504	YELLOW	CENTRAL	Lexington, KY	*P. virginiana*	28-Aug-2014
LL095	38.014	-84.504	YELLOW	CENTRAL	Lexington, KY	*P. virginiana*	28-Aug-2014
LL096	38.014	-84.504	YELLOW	CENTRAL	Lexington, KY	*P. virginiana*	28-Aug-2014
LL097	38.044	-84.497	YELLOW	CENTRAL	Lexington, KY	*P. mugho*	3-Sep-2014
LL098	38.044	-84.497	YELLOW	CENTRAL	Lexington, KY	*P. mugho*	3-Sep-2014
LL099	38.044	-84.497	YELLOW	CENTRAL	Lexington, KY	*P. mugho*	3-Sep-2014
LL100	38.023	-84.494	YELLOW	CENTRAL	Lexington, KY	*P. nigra*	3-Sep-2014
LL101	38.014	-84.504	YELLOW	CENTRAL	Lexington, KY	*P. rigida*	4-Sep-2014
LL102	38.014	-84.504	YELLOW	CENTRAL	Lexington, KY	*P. rigida*	4-Sep-2014
LL103	38.014	-84.504	YELLOW	CENTRAL	Lexington, KY	*P. echinata*	4-Sep-2014
LL104	38.014	-84.504	YELLOW	CENTRAL	Lexington, KY	*P. echinata*	4-Sep-2014
LL105	38.014	-84.504	YELLOW	CENTRAL	Lexington, KY	*P. echinata*	4-Sep-2014
LL106	38.014	-84.504	YELLOW	CENTRAL	Lexington, KY	*P. virginiana*	4-Sep-2014
LL107	38.014	-84.504	YELLOW	CENTRAL	Lexington, KY	*P. virginiana*	4-Sep-2014
LL108	38.014	-84.504	YELLOW	CENTRAL	Lexington, KY	*P. virginiana*	4-Sep-2014
LL109	38.024	-84.532	YELLOW	CENTRAL	Lexington, KY	*P. mugho*	21-Sep-2014
LL110	38.024	-84.532	YELLOW	CENTRAL	Lexington, KY	*P. mugho*	21-Sep-2014
LL111	38.024	-84.532	YELLOW	CENTRAL	Lexington, KY	*P. mugho*	21-Sep-2014
LL112	38.024	-84.532	YELLOW	CENTRAL	Lexington, KY	*P. mugho*	21-Sep-2014
LL113	38.024	-84.532	YELLOW	CENTRAL	Lexington, KY	*P. mugho*	21-Sep-2014
LL116	38.014	-84.504	YELLOW	CENTRAL	Lexington, KY	*P. rigida*	2-Jun-2015
LL117	37.984	-84.418	YELLOW	CENTRAL	Lexington, KY	*P. taeda*	4-Jun-2015
LL121	38.014	-84.504	YELLOW	CENTRAL	Lexington, KY	*P. rigida*	17-Jun-2015
LL122	38.014	-84.504	YELLOW	CENTRAL	Lexington, KY	*P. rigida*	17-Jun-2015
LL123	38.033	-84.507	YELLOW	CENTRAL	Lexington, KY	*P. mugho*	18-Jun-2015
LL124	38.033	-84.507	YELLOW	CENTRAL	Lexington, KY	*P. mugho*	18-Jun-2015
LL125	37.984	-84.418	YELLOW	CENTRAL	Lexington, KY	*P. taeda*	18-Jun-2015

LL126	37.984	-84.418	YELLOW	CENTRAL	Lexington, KY	*P. taeda*	18-Jun-2015
LL127	37.984	-84.418	YELLOW	CENTRAL	Lexington, KY	*P. taeda*	18-Jun-2015
LL128	38.044	-84.497	YELLOW	CENTRAL	Lexington, KY	*P. mugho*	18-Jun-2015
LL129	38.044	-84.497	YELLOW	CENTRAL	Lexington, KY	*P. mugho*	18-Jun-2015
LL130	38.044	-84.497	YELLOW	CENTRAL	Lexington, KY	*P. mugho*	18-Jun-2015
LL131	38.044	-84.497	YELLOW	CENTRAL	Lexington, KY	*P. mugho*	18-Jun-2015
LL132	38.024	-84.532	YELLOW	CENTRAL	Lexington, KY	*P. mugho*	19-Jun-2015
LL133	38.024	-84.532	YELLOW	CENTRAL	Lexington, KY	*P. mugho*	19-Jun-2015
LL134	38.024	-84.532	YELLOW	CENTRAL	Lexington, KY	*P. mugho*	19-Jun-2015
LL135	38.024	-84.532	YELLOW	CENTRAL	Lexington, KY	*P. mugho*	19-Jun-2015
LL136	38.014	-84.504	YELLOW	CENTRAL	Lexington, KY	*P. virginiana*	19-Jun-2015
LL137	38.014	-84.504	YELLOW	CENTRAL	Lexington, KY	*P. virginiana*	19-Jun-2015
LL138	38.014	-84.504	YELLOW	CENTRAL	Lexington, KY	*P. echinata*	19-Jun-2015
LL139	38.014	-84.504	YELLOW	CENTRAL	Lexington, KY	*P. echinata*	19-Jun-2015
LL140	38.014	-84.504	YELLOW	CENTRAL	Lexington, KY	*P. virginiana*	19-Jun-2015
LL142	35.980	-85.015	YELLOW	CENTRAL	Crossville, TN	*P. virginiana*	23-Jun-2015
LL143	35.980	-85.015	YELLOW	CENTRAL	Crossville, TN	*P. virginiana*	23-Jun-2015
LL144	35.980	-85.015	YELLOW	CENTRAL	Crossville, TN	*P. virginiana*	23-Jun-2015
LL145	35.980	-85.015	YELLOW	CENTRAL	Crossville, TN	*P. virginiana*	23-Jun-2015
LL146	35.980	-85.015	YELLOW	CENTRAL	Crossville, TN	*P. virginiana*	23-Jun-2015
LL147	35.980	-85.015	YELLOW	CENTRAL	Crossville, TN	*P. virginiana*	23-Jun-2015
LL148	38.015	-84.501	YELLOW	CENTRAL	Lexington, KY	*P. virginiana*	25-Jun-2015
LL149	38.015	-84.501	YELLOW	CENTRAL	Lexington, KY	*P. virginiana*	25-Jun-2015
LL150	38.015	-84.501	YELLOW	CENTRAL	Lexington, KY	*P. virginiana*	25-Jun-2015
LL151	38.015	-84.501	YELLOW	CENTRAL	Lexington, KY	*P. virginiana*	25-Jun-2015
LL152	38.015	-84.501	YELLOW	CENTRAL	Lexington, KY	*P. virginiana*	25-Jun-2015
LL153	38.015	-84.501	YELLOW	CENTRAL	Lexington, KY	*P. virginiana*	25-Jun-2015
LL154	38.015	-84.501	YELLOW	CENTRAL	Lexington, KY	*P. virginiana*	25-Jun-2015
LL155	38.015	-84.501	YELLOW	CENTRAL	Lexington, KY	*P. virginiana*	25-Jun-2015

LL156	38.015	-84.501	YELLOW	CENTRAL	Lexington, KY	*P. virginiana*	25-Jun-2015
LL157	38.015	-84.501	YELLOW	CENTRAL	Lexington, KY	*P. virginiana*	25-Jun-2015
LL158	38.015	-84.501	YELLOW	CENTRAL	Lexington, KY	*P. virginiana*	25-Jun-2015
LL159	38.015	-84.501	YELLOW	CENTRAL	Lexington, KY	*P. virginiana*	25-Jun-2015
LL160	38.015	-84.501	YELLOW	CENTRAL	Lexington, KY	*P. virginiana*	25-Jun-2015
LL161	38.015	-84.501	YELLOW	CENTRAL	Lexington, KY	*P. virginiana*	25-Jun-2015
LL162	38.015	-84.501	YELLOW	CENTRAL	Lexington, KY	*P. virginiana*	25-Jun-2015
LL163	38.015	-84.501	YELLOW	CENTRAL	Lexington, KY	*P. virginiana*	25-Jun-2015
LL164	38.015	-84.501	YELLOW	CENTRAL	Lexington, KY	*P. virginiana*	25-Jun-2015
LL165	38.015	-84.501	YELLOW	CENTRAL	Lexington, KY	*P. virginiana*	25-Jun-2015
LL166	38.015	-84.501	YELLOW	CENTRAL	Lexington, KY	*P. virginiana*	25-Jun-2015
LL167	38.015	-84.501	YELLOW	CENTRAL	Lexington, KY	*P. virginiana*	25-Jun-2015
LL168	38.015	-84.501	YELLOW	CENTRAL	Lexington, KY	*P. virginiana*	25-Jun-2015
LL169	38.015	-84.501	YELLOW	CENTRAL	Lexington, KY	*P. virginiana*	29-Jun-2015
LL170	38.015	-84.501	YELLOW	CENTRAL	Lexington, KY	*P. virginiana*	29-Jun-2015
LL171	38.015	-84.501	YELLOW	CENTRAL	Lexington, KY	*P. virginiana*	29-Jun-2015
LL172	38.015	-84.501	YELLOW	CENTRAL	Lexington, KY	*P. virginiana*	29-Jun-2015
LL173	38.015	-84.501	YELLOW	CENTRAL	Lexington, KY	*P. virginiana*	29-Jun-2015
LL174	38.015	-84.501	YELLOW	CENTRAL	Lexington, KY	*P. virginiana*	29-Jun-2015
LL175	38.015	-84.501	YELLOW	CENTRAL	Lexington, KY	*P. virginiana*	29-Jun-2015
LL176	38.015	-84.501	YELLOW	CENTRAL	Lexington, KY	*P. virginiana*	29-Jun-2015
LL177	38.015	-84.501	YELLOW	CENTRAL	Lexington, KY	*P. virginiana*	29-Jun-2015
LL178	38.014	-84.504	YELLOW	CENTRAL	Lexington, KY	*P. virginiana*	30-Jun-2015
LL179	38.014	-84.504	YELLOW	CENTRAL	Lexington, KY	*P. echinata*	3-Jul-2015
LL180	38.402	-85.586	YELLOW	CENTRAL	Goshen, KY	*P. echinata*	4-Jul-2015
LL181	38.402	-85.586	YELLOW	CENTRAL	Goshen, KY	*P. echinata*	4-Jul-2015
LL184	38.014	-84.504	YELLOW	CENTRAL	Lexington, KY	*P. rigida*	8-Jul-2015
LL191	38.014	-84.504	YELLOW	CENTRAL	Lexington, KY	*P. echinata*	20-Jul-2015
LL193	37.806	-83.678	YELLOW	CENTRAL	Stanton, KY	*P. virginiana*	5-Aug-2015

LL194	37.806	-83.678	YELLOW	CENTRAL	Stanton, KY	*P. virginiana*	5-Aug-2015
LL195	37.806	-83.678	YELLOW	CENTRAL	Stanton, KY	*P. virginiana*	5-Aug-2015
LL196	37.805	-83.656	YELLOW	CENTRAL	Stanton, KY	*P. virginiana*	5-Aug-2015
LL200	38.014	-84.504	YELLOW	CENTRAL	Lexington, KY	*P. virginiana*	13-Aug-2015
LL204	38.014	-84.504	YELLOW	CENTRAL	Lexington, KY	*P. virginiana*	20-Aug-2015
LL205	38.014	-84.504	YELLOW	CENTRAL	Lexington, KY	*P. virginiana*	20-Aug-2015
LL206	38.014	-84.504	YELLOW	CENTRAL	Lexington, KY	*P. virginiana*	20-Aug-2015
LL207	38.014	-84.504	YELLOW	CENTRAL	Lexington, KY	*P. virginiana*	20-Aug-2015
LL208	38.033	-84.507	YELLOW	CENTRAL	Lexington, KY	*P. mugho*	24-Aug-2015
LL209	38.014	-84.504	YELLOW	CENTRAL	Lexington, KY	*P. virginiana*	28-Aug-2015
LL210	38.014	-84.504	YELLOW	CENTRAL	Lexington, KY	*P. virginiana*	28-Aug-2015
LL211	38.014	-84.504	YELLOW	CENTRAL	Lexington, KY	*P. virginiana*	28-Aug-2015
LL212	38.014	-84.504	YELLOW	CENTRAL	Lexington, KY	*P. virginiana*	28-Aug-2015
LL213	38.014	-84.504	YELLOW	CENTRAL	Lexington, KY	*P. virginiana*	28-Aug-2015
LL214	38.014	-84.504	YELLOW	CENTRAL	Lexington, KY	*P. virginiana*	28-Aug-2015
LL215	38.014	-84.504	YELLOW	CENTRAL	Lexington, KY	*P. virginiana*	28-Aug-2015
LL216	38.014	-84.504	YELLOW	CENTRAL	Lexington, KY	*P. echinata*	28-Aug-2015
LL218	38.014	-84.504	YELLOW	CENTRAL	Lexington, KY	*P. virginiana*	10-Sep-2015
LL219	38.014	-84.504	YELLOW	CENTRAL	Lexington, KY	*P. virginiana*	10-Sep-2015
LL220	38.014	-84.504	YELLOW	CENTRAL	Lexington, KY	*P. virginiana*	10-Sep-2015
LL221	38.014	-84.504	YELLOW	CENTRAL	Lexington, KY	*P. virginiana*	10-Sep-2015
LL222	38.014	-84.504	YELLOW	CENTRAL	Lexington, KY	*P. virginiana*	10-Sep-2015
LL223	38.014	-84.504	YELLOW	CENTRAL	Lexington, KY	*P. virginiana*	10-Sep-2015
LL224	38.024	-84.532	YELLOW	CENTRAL	Lexington, KY	*P. mugho*	12-Sep-2015
LL225	38.014	-84.504	YELLOW	CENTRAL	Lexington, KY	*P. virginiana*	14-Sep-2015
LL226	38.014	-84.504	YELLOW	CENTRAL	Lexington, KY	*P. virginiana*	14-Sep-2015
LL227	38.014	-84.504	YELLOW	CENTRAL	Lexington, KY	*P. virginiana*	14-Sep-2015
LL228	38.014	-84.504	YELLOW	CENTRAL	Lexington, KY	*P. virginiana*	14-Sep-2015
LL229	38.014	-84.504	YELLOW	CENTRAL	Lexington, KY	*P. virginiana*	14-Sep-2015

LL230	38.014	-84.504	YELLOW	CENTRAL	Lexington, KY	*P. virginiana*	14-Sep-2015
LL242	37.984	-84.418	YELLOW	CENTRAL	Lexington, KY	*P. taeda*	7-Jun-2016
LL243	37.984	-84.418	YELLOW	CENTRAL	Lexington, KY	*P. taeda*	7-Jun-2016
LL245	38.014	-84.504	YELLOW	CENTRAL	Lexington, KY	*P. virginiana*	9-Jun-2016
LL246	37.984	-84.418	YELLOW	CENTRAL	Lexington, KY	*P. taeda*	13-Jun-2016
LL247	38.014	-84.504	YELLOW	CENTRAL	Lexington, KY	*P. virginiana*	16-Jun-2016
LL248	38.014	-84.504	YELLOW	CENTRAL	Lexington, KY	*P. virginiana*	16-Jun-2016
LL249	38.014	-84.504	YELLOW	CENTRAL	Lexington, KY	*P. virginiana*	16-Jun-2016
LL250	38.014	-84.504	YELLOW	CENTRAL	Lexington, KY	*P. virginiana*	16-Jun-2016
LL251	38.014	-84.504	YELLOW	CENTRAL	Lexington, KY	*P. virginiana*	16-Jun-2016
LL252	38.044	-84.497	YELLOW	CENTRAL	Lexington, KY	*P. mugho*	23-Jun-2016
LL253	38.044	-84.497	YELLOW	CENTRAL	Lexington, KY	*P. mugho*	23-Jun-2016
LL254	38.014	-84.504	YELLOW	CENTRAL	Lexington, KY	*P. rigida*	24-Jun-2016
LL255	38.014	-84.504	YELLOW	CENTRAL	Lexington, KY	*P. virginiana*	24-Jun-2016
LL256	38.014	-84.504	YELLOW	CENTRAL	Lexington, KY	*P. virginiana*	24-Jun-2016
LL257	38.014	-84.504	YELLOW	CENTRAL	Lexington, KY	*P. virginiana*	24-Jun-2016
LL258	38.014	-84.504	YELLOW	CENTRAL	Lexington, KY	*P. virginiana*	24-Jun-2016
LL259	38.014	-84.504	YELLOW	CENTRAL	Lexington, KY	*P. virginiana*	24-Jun-2016
LL260	38.014	-84.504	YELLOW	CENTRAL	Lexington, KY	*P. virginiana*	24-Jun-2016
LL261	38.044	-84.497	YELLOW	CENTRAL	Lexington, KY	*P. mugho*	24-Jun-2016
LL262	38.044	-84.497	YELLOW	CENTRAL	Lexington, KY	*P. mugho*	24-Jun-2016
LL263	38.044	-84.497	YELLOW	CENTRAL	Lexington, KY	*P. mugho*	24-Jun-2016
LL264	38.044	-84.497	YELLOW	CENTRAL	Lexington, KY	*P. mugho*	24-Jun-2016
LL265	38.044	-84.497	YELLOW	CENTRAL	Lexington, KY	*P. mugho*	24-Jun-2016
LL266	38.044	-84.497	YELLOW	CENTRAL	Lexington, KY	*P. mugho*	24-Jun-2016
LL267	38.044	-84.497	YELLOW	CENTRAL	Lexington, KY	*P. mugho*	24-Jun-2016
LL268	38.044	-84.497	YELLOW	CENTRAL	Lexington, KY	*P. mugho*	24-Jun-2016
LL269	38.044	-84.497	YELLOW	CENTRAL	Lexington, KY	*P. mugho*	24-Jun-2016
LL270	38.044	-84.497	YELLOW	CENTRAL	Lexington, KY	*P. mugho*	24-Jun-2016

LL271	38.044	-84.497	YELLOW	CENTRAL	Lexington, KY	*P. mugho*	24-Jun-2016
LL272	38.044	-84.497	YELLOW	CENTRAL	Lexington, KY	*P. mugho*	24-Jun-2016
LL273	38.044	-84.497	YELLOW	CENTRAL	Lexington, KY	*P. mugho*	24-Jun-2016
LL274	38.044	-84.497	YELLOW	CENTRAL	Lexington, KY	*P. mugho*	24-Jun-2016
LL275	38.044	-84.497	YELLOW	CENTRAL	Lexington, KY	*P. mugho*	24-Jun-2016
LL276	38.044	-84.497	YELLOW	CENTRAL	Lexington, KY	*P. mugho*	24-Jun-2016
LL277	35.980	-85.015	YELLOW	CENTRAL	Crossville, TN	*P. virginiana*	25-Jun-2016
LL278	35.980	-85.015	YELLOW	CENTRAL	Crossville, TN	*P. virginiana*	25-Jun-2016
LL279	38.014	-84.504	YELLOW	CENTRAL	Lexington, KY	*P. virginiana*	24-Aug-2016
LL280	38.014	-84.504	YELLOW	CENTRAL	Lexington, KY	*P. virginiana*	24-Aug-2016
LL281	38.014	-84.504	YELLOW	CENTRAL	Lexington, KY	*P. virginiana*	24-Aug-2016
LL282	38.014	-84.504	YELLOW	CENTRAL	Lexington, KY	*P. virginiana*	24-Aug-2016
LL283	38.014	-84.504	YELLOW	CENTRAL	Lexington, KY	*P. virginiana*	24-Aug-2016
LL284	38.014	-84.504	YELLOW	CENTRAL	Lexington, KY	*P. virginiana*	24-Aug-2016
LL285	38.014	-84.504	YELLOW	CENTRAL	Lexington, KY	*P. virginiana*	24-Aug-2016
LL286	38.014	-84.504	YELLOW	CENTRAL	Lexington, KY	*P. virginiana*	24-Aug-2016
LL287	38.014	-84.504	YELLOW	CENTRAL	Lexington, KY	*P. virginiana*	24-Aug-2016
LL288	38.014	-84.504	YELLOW	CENTRAL	Lexington, KY	*P. virginiana*	24-Aug-2016
LL289	38.014	-84.504	YELLOW	CENTRAL	Lexington, KY	*P. virginiana*	2-Sep-2016
LL290	38.014	-84.504	YELLOW	CENTRAL	Lexington, KY	*P. virginiana*	2-Sep-2016
LL291	38.014	-84.504	YELLOW	CENTRAL	Lexington, KY	*P. virginiana*	2-Sep-2016
LL292	38.014	-84.504	YELLOW	CENTRAL	Lexington, KY	*P. virginiana*	2-Sep-2016
LL293	38.014	-84.504	YELLOW	CENTRAL	Lexington, KY	*P. virginiana*	2-Sep-2016
LL294	38.014	-84.504	YELLOW	CENTRAL	Lexington, KY	*P. virginiana*	2-Sep-2016
LL295	38.014	-84.504	YELLOW	CENTRAL	Lexington, KY	*P. virginiana*	2-Sep-2016
LL296	38.014	-84.504	YELLOW	CENTRAL	Lexington, KY	*P. virginiana*	2-Sep-2016
LL297	38.014	-84.504	YELLOW	CENTRAL	Lexington, KY	*P. virginiana*	2-Sep-2016
LL298	38.014	-84.504	YELLOW	CENTRAL	Lexington, KY	*P. virginiana*	2-Sep-2016
LL299	38.014	-84.504	YELLOW	CENTRAL	Lexington, KY	*P. virginiana*	2-Sep-2016

LL300	38.014	-84.504	YELLOW	CENTRAL	Lexington, KY	*P. virginiana*	2-Sep-2016
LL301	38.014	-84.504	YELLOW	CENTRAL	Lexington, KY	*P. virginiana*	2-Sep-2016
LL302	38.014	-84.504	YELLOW	CENTRAL	Lexington, KY	*P. virginiana*	2-Sep-2016
LL303	38.014	-84.504	YELLOW	CENTRAL	Lexington, KY	*P. virginiana*	2-Sep-2016
LL304	38.014	-84.504	YELLOW	CENTRAL	Lexington, KY	*P. virginiana*	2-Sep-2016
LL305	38.014	-84.504	YELLOW	CENTRAL	Lexington, KY	*P. virginiana*	2-Sep-2016
RB001	40.680	-74.234	YELLOW	CENTRAL	Union, NJ	*P. strobus*	5-Sep-2009
RB002	38.171	-83.556	YELLOW	CENTRAL	Morehead, KY	*P. rigida*	25-Jul-2011
RB003	40.603	-74.475	MIXED	CENTRAL	Greenbrook, NJ	*P. mugho*	1-Jul-2011
RB004	44.350	-89.822	YELLOW	NORTH	Grand Rapids, WI	*P. banksiana*	28-Jul-2011
RB006	37.997	-84.672	YELLOW	CENTRAL	London, KY	*P. echinata*	2-Aug-2011
RB008	32.138	-82.969	YELLOW	SOUTH	Helena, GA	*P. elliottii*	4-Aug-2011
RB009	32.523	-83.496	YELLOW	SOUTH	Dry Branch, GA	*P. taeda*	7-Aug-2011
RB010	32.523	-83.496	YELLOW	SOUTH	Dry Branch, GA	*P. echinata*	7-Aug-2011
RB011	32.523	-83.496	YELLOW	SOUTH	Dry Branch, GA	*P. echinata*	7-Aug-2011
RB012	32.523	-83.496	YELLOW	SOUTH	Dry Branch, GA	*P. echinata*	7-Aug-2011
RB015	44.461	-85.992	YELLOW	NORTH	Springdale Township, MI	*P. banksiana*	4-Aug-2011
RB016	37.066	-84.159	YELLOW	CENTRAL	Laurel Co, KY	*P. echinata*	17-Aug-2011
RB017	37.984	-84.511	YELLOW	CENTRAL	Lexingon, KY	*P. mugho*	18-Aug-2011
RB018	43.797	-71.915	YELLOW	CENTRAL	Dorchester, NH	*P. resinosa*	5-Aug-2011
RB019	37.066	-84.159	YELLOW	CENTRAL	Laurel Co, KY	*P. echinata*	6-Sep-2011
RB020	37.066	-84.159	YELLOW	CENTRAL	Laurel Co, KY	*P. echinata*	6-Sep-2011
RB021	37.066	-84.159	YELLOW	CENTRAL	Laurel Co, KY	*P. echinata*	6-Sep-2011
RB022	38.024	-84.494	YELLOW	CENTRAL	Lexington, KY	*P. nigra*	7-Sep-2011
RB024	44.936	-75.668	YELLOW	NORTH	Pattersons Corners, ON	*young pinus resinosa*	31-Aug-2011
RB025	41.268	-78.280	MIXED	CENTRAL	Jay Township, PN	*P. mugho*	17-Oct-2011
RB027	33.990	-83.796	YELLOW	CENTRAL/SOUTH	Auburn, GA	*P. echinata*	25-Oct-2011
RB028	39.690	-74.593	WHITE	CENTRAL	Washington, NJ	*P. rigida*	9-Sep-2010

RB029	38.209	-84.390	YELLOW	CENTRAL	Scotch Plains, NJ	*P. sylvestris*	9/2010
RB039	37.071	-84.211	YELLOW	CENTRAL	London, KY	*P. echinata*	29-May-2012
RB040	38.209	-84.390	YELLOW	CENTRAL	Lexington, KY	*P. sylvestris*	29-May-2012
RB042	38.024	-84.494	YELLOW	CENTRAL	Lexington, KY	*P. nigra*	30-May-2012
RB044	27.692	-82.420	YELLOW	SOUTH	Ruskin, FL	*P. elliottii*	5-Jun-2012
RB046	27.618	-81.815	YELLOW	SOUTH	Bowling Green, FL	*P. palustris*	8-Jun-2012
RB047	27.618	-81.815	YELLOW	SOUTH	Bowling Green, FL	*P. palustris*	8-Jun-2012
RB048	27.618	-81.815	YELLOW	SOUTH	Bowling Green, FL	*P. palustris*	8-Jun-2012
RB049	29.507	-81.860	YELLOW	SOUTH	Interlachen, FL	*P. palustris*	9-Jun-2012
RB050	29.507	-81.860	YELLOW	SOUTH	Interlachen, FL	*P. palustris*	9-Jun-2012
RB051	29.507	-81.860	YELLOW	SOUTH	Interlachen, FL	*P. palustris*	9-Jun-2012
RB052	29.507	-81.860	YELLOW	SOUTH	Interlachen, FL	*P. palustris*	9-Jun-2012
RB053	29.507	-81.860	YELLOW	SOUTH	Interlachen, FL	*P. palustris*	9-Jun-2012
RB054	29.507	-81.860	YELLOW	SOUTH	Interlachen, FL	*P. palustris*	9-Jun-2012
RB055	29.507	-81.860	YELLOW	SOUTH	Interlachen, FL	*P. palustris*	9-Jun-2012
RB056	29.507	-81.860	YELLOW	SOUTH	Interlachen, FL	*P. palustris*	9-Jun-2012
RB057	29.320	-81.727	YELLOW	SOUTH	Salt Springs, FL	*P. palustris*	10-Jun-2012
RB058	29.320	-81.727	YELLOW	SOUTH	Salt Springs, FL	*P. palustris*	10-Jun-2012
RB059	29.320	-81.727	YELLOW	SOUTH	Salt Springs, FL	*P. palustris*	10-Jun-2012
RB060	29.320	-81.727	YELLOW	SOUTH	Salt Springs, FL	*P. palustris*	10-Jun-2012
RB061	29.320	-81.727	YELLOW	SOUTH	Salt Springs, FL	*P. palustris*	10-Jun-2012
RB062	29.320	-81.727	YELLOW	SOUTH	Salt Springs, FL	*P. palustris*	10-Jun-2012
RB063	29.320	-81.727	YELLOW	SOUTH	Salt Springs, FL	*P. palustris*	10-Jun-2012
RB064	29.507	-82.960	YELLOW	SOUTH	Fanning Springs, FL	*P. palustris*	11-Jun-2012
RB065	29.507	-82.960	YELLOW	SOUTH	Fanning Springs, FL	*P. palustris*	11-Jun-2012
RB066	29.508	-82.958	YELLOW	SOUTH	Fanning Springs, FL	*P. palustris*	11-Jun-2012
RB067	29.508	-82.958	YELLOW	SOUTH	Fanning Springs, FL	*P. palustris*	11-Jun-2012
RB068	30.191	-84.370	YELLOW	SOUTH	Crawfordville, FL	*P. palustris*	12-Jun-2012
RB069	30.191	-84.370	YELLOW	SOUTH	Crawfordville, FL	*P. palustris*	12-Jun-2012

RB071	32.843	-87.952	YELLOW	CENTRAL/SOUTH	Eutaw, AL	*P. echinata*	16-Jun-2012
RB073	38.014	-84.504	YELLOW	CENTRAL	Lexington, KY	*P. rigida*	21-Jun-2012
RB074	37.984	-84.418	YELLOW	CENTRAL	Lexington, KY	*P. taeda*	27-Jun-2012
RB075	32.239	-80.859	YELLOW	SOUTH	Bluffton, SC	*P. palustris*	25-Jun-2012
RB076	38.014	-84.504	YELLOW	CENTRAL	Lexington, KY	*P. virginiana*	5-Jul-2012
RB077	43.759	-85.741	YELLOW	NORTH	Brohman, MI	*P. banksiana*	20-Jul-2012
RB078	43.759	-85.741	YELLOW	NORTH	Brohman, MI	*P. banksiana*	20-Jul-2012
RB079	43.759	-85.741	YELLOW	NORTH	Brohman, MI	*P. banksiana*	20-Jul-2012
RB080	43.796	-85.740	YELLOW	NORTH	Bitely, MI	*P. banksiana*	20-Jul-2012
RB081	43.796	-85.740	YELLOW	NORTH	Bitely, MI	*P. banksiana*	20-Jul-2012
RB082	43.796	-85.740	YELLOW	NORTH	Bitely, MI	*P. banksiana*	20-Jul-2012
RB083	43.796	-85.740	YELLOW	NORTH	Bitely, MI	*P. banksiana*	20-Jul-2012
RB084	43.796	-85.740	YELLOW	NORTH	Bitely, MI	*P. banksiana*	20-Jul-2012
RB085	43.796	-85.740	YELLOW	NORTH	Bitely, MI	*P. banksiana*	20-Jul-2012
RB086	43.796	-85.740	YELLOW	NORTH	Bitely, MI	*P. banksiana*	20-Jul-2012
RB087	43.796	-85.740	YELLOW	NORTH	Bitely, MI	*P. banksiana*	20-Jul-2012
RB088	43.796	-85.740	YELLOW	NORTH	Bitely, MI	*P. banksiana*	20-Jul-2012
RB089	43.796	-85.740	YELLOW	NORTH	Bitely, MI	*P. banksiana*	20-Jul-2012
RB090	44.657	-84.414	YELLOW	NORTH	Grayling, MI	*P. sylvestris*	21-Jul-2012
RB091	44.657	-84.414	YELLOW	NORTH	Grayling, MI	*P. banksiana*	21-Jul-2012
RB092	44.657	-84.414	YELLOW	NORTH	Grayling, MI	*P. banksiana*	21-Jul-2012
RB093	44.657	-84.414	YELLOW	NORTH	Grayling, MI	*P. banksiana*	21-Jul-2012
RB094	45.504	-84.615	YELLOW	NORTH	Glaque Beach, MI	*P. banksiana*	21-Jul-2012
RB095	46.094	-85.339	YELLOW	NORTH	Naubinway, MI	*P. banksiana*	22-Jul-2012
RB096	46.096	-85.394	YELLOW	NORTH	Naubinway, MI	*P. banksiana*	22-Jul-2012
RB097	46.096	-85.394	YELLOW	NORTH	Naubinway, MI	*P. banksiana*	22-Jul-2012
RB098	46.096	-85.394	YELLOW	NORTH	Naubinway, MI	*P. banksiana*	22-Jul-2012
RB099	45.924	-86.303	YELLOW	NORTH	Manistique, MI	*P. banksiana*	22-Jul-2012
RB100	46.354	-89.179	YELLOW	NORTH	Bruce Crossing, MI	*P. banksiana*	23-Jul-2012

RB101	44.985	-88.449	YELLOW	NORTH	How, WI	*P. resinosa*	24-Jul-2012
RB102	44.985	-88.449	YELLOW	NORTH	How, WI	*P. resinosa*	24-Jul-2012
RB103	44.985	-88.449	YELLOW	NORTH	How, WI	*P. resinosa*	24-Jul-2012
RB104	44.985	-88.449	YELLOW	NORTH	How, WI	*P. resinosa*	24-Jul-2012
RB105	44.985	-88.449	YELLOW	NORTH	How, WI	*P. resinosa*	24-Jul-2012
RB106	37.913	-79.896	WHITE	CENTRAL	Valley Springs, VA	*P. virginiana*	2-Aug-2012
RB107	38.212	-79.719	WHITE	CENTRAL	Mountain Grove, VA	*P. rigida*	2-Aug-2012
RB108	38.678	-79.399	WHITE	CENTRAL	Deer Run, WV	*P. rigida*	2-Aug-2012
RB110	39.934	-74.533	WHITE	CENTRAL	Browns Mills, NJ	*P. rigida*	5-Aug-2012
RB112	39.621	-74.428	WHITE	CENTRAL	Tuckerton, NJ	*P. rigida*	6-Aug-2012
RB118	38.592	-79.172	WHITE	CENTRAL	Eastern, WV	*P. virginiana*	9-Aug-2012
RB119	37.713	-79.367	WHITE	CENTRAL	Buena Vista, VA	*P. virginiana*	10-Aug-2012
RB120	38.209	-84.390	YELLOW	CENTRAL	Lexington, KY	*P. sylvestris*	20-Aug-2012
RB121	38.209	-84.390	YELLOW	CENTRAL	Lexington, KY	*P. sylvestris*	20-Aug-2012
RB122	38.010	-84.302	YELLOW	CENTRAL	Lexington, KY	*P. echinata*	20-Aug-2012
RB124	38.209	-84.390	YELLOW	CENTRAL	Scotch Plains, NJ	*P. sylvestris*	26-Aug-2012
RB125	39.717	-78.280	YELLOW	CENTRAL	Sideling Hill, MD	*P. mugho*	31-Aug-2012
RB126	38.014	-84.504	YELLOW	CENTRAL	Lexington, KY	*P. virginiana*	31-Aug-2012
RB127	38.014	-84.504	YELLOW	CENTRAL	Lexington, KY	*P. virginiana*	31-Aug-2012
RB128	38.014	-84.504	YELLOW	CENTRAL	Lexington, KY	*P. virginiana*	31-Aug-2012
RB129	38.014	-84.504	YELLOW	CENTRAL	Lexington, KY	*P. echinata*	31-Aug-2012
RB130	32.277	-80.983	YELLOW	SOUTH	Bluffton SC	*P. palustris*	30-Aug-2012
RB131	32.277	-80.983	YELLOW	SOUTH	Bluffton SC	*P. taeda*	30-Aug-2012
RB132	38.014	-84.504	YELLOW	CENTRAL	Lexington, KY	*P. rigida*	14-Sep-2012
RB133	38.014	-84.504	YELLOW	CENTRAL	Lexington, KY	*P. virginiana*	14-Sep-2012
RB134	38.014	-84.504	YELLOW	CENTRAL	Lexington, KY	*P. virginiana*	14-Sep-2012
RB135	38.014	-84.504	YELLOW	CENTRAL	Lexington, KY	*P. virginiana*	14-Sep-2012
RB136	43.759	-85.741	YELLOW	NORTH	Bitely, MI	*P. resinosa*	23-Oct-2012
RB137	27.618	-81.815	YELLOW	SOUTH	Bowling Green, FL	*P. elliottii*	23-Oct-2012

RB138	27.618	-81.815	YELLOW	SOUTH	Bowling Green, FL	*P. elliottii*	23-Oct-2012
RB139	27.618	-81.815	YELLOW	SOUTH	Bowling Green, FL	*P. elliottii*	23-Oct-2012
RB141	38.014	-84.504	MIXED	CENTRAL	Lexington, KY	*P. virginiana*	26-Jun-2013
RB142	38.209	-84.390	YELLOW	CENTRAL	Lexington, KY	*P. sylvestris*	26-Jun-2013
RB143	38.209	-84.390	YELLOW	CENTRAL	Lexington, KY	*P. sylvestris*	26-Jun-2013
RB144	38.209	-84.390	YELLOW	CENTRAL	Lexington, KY	*P. sylvestris*	26-Jun-2013
RB145	38.209	-84.390	YELLOW	CENTRAL	Lexington, KY	*P. sylvestris*	26-Jun-2013
RB146	38.209	-84.390	YELLOW	CENTRAL	Lexington, KY	*P. sylvestris*	26-Jun-2013
RB147	38.209	-84.390	YELLOW	CENTRAL	Lexington, KY	*P. sylvestris*	26-Jun-2013
RB148	38.209	-84.390	YELLOW	CENTRAL	Lexington, KY	*P. sylvestris*	26-Jun-2013
RB149	38.209	-84.390	YELLOW	CENTRAL	Lexington, KY	*P. sylvestris*	26-Jun-2013
RB150	38.209	-84.390	YELLOW	CENTRAL	Lexington, KY	*P. sylvestris*	26-Jun-2013
RB151	38.209	-84.390	YELLOW	CENTRAL	Lexington, KY	*P. sylvestris*	26-Jun-2013
RB152	38.209	-84.390	YELLOW	CENTRAL	Lexington, KY	*P. sylvestris*	26-Jun-2013
RB153	38.209	-84.390	YELLOW	CENTRAL	Lexington, KY	*P. sylvestris*	26-Jun-2013
RB154	38.209	-84.390	YELLOW	CENTRAL	Lexington, KY	*P. sylvestris*	26-Jun-2013
RB155	38.209	-84.390	YELLOW	CENTRAL	Lexington, KY	*P. sylvestris*	26-Jun-2013
RB156	38.209	-84.390	YELLOW	CENTRAL	Lexington, KY	*P. sylvestris*	26-Jun-2013
RB157	38.209	-84.390	YELLOW	CENTRAL	Lexington, KY	*P. sylvestris*	26-Jun-2013
RB159	37.984	-84.418	YELLOW	CENTRAL	Lexington, KY	*P. taeda*	27-Jun-2013
RB160	37.984	-84.418	YELLOW	CENTRAL	Lexington, KY	*P. taeda*	27-Jun-2013
RB161	37.984	-84.418	YELLOW	CENTRAL	Lexington, KY	*P. taeda*	27-Jun-2013
RB162	38.024	-84.494	YELLOW	CENTRAL	Lexington, KY	*P. nigra*	2-Jul-2013
RB163	35.280	-82.117	YELLOW	NORTH	Tryon, NC	*P. taeda*	3-Jul-2013
RB164	35.280	-82.117	YELLOW	NORTH	Tryon, NC	*P. virginiana*	3-Jul-2013
RB165	35.280	-82.118	YELLOW	NORTH	Tryon, NC	*P. virginiana*	3-Jul-2013
RB167	35.183	-81.963	YELLOW	SOUTH	Chesnee,SC	*P. virginiana*	4-Jul-2013
RB168	35.183	-81.963	YELLOW	SOUTH	Chesnee,SC	*P. virginiana*	4-Jul-2013
RB171	35.183	-81.963	YELLOW	SOUTH	Chesnee,SC	*P. taeda*	4-Jul-2013

RB172	35.183	-81.963	YELLOW	SOUTH	Chesnee,SC	*P. taeda*	4-Jul-2013
RB173	35.183	-81.963	YELLOW	SOUTH	Chesnee,SC	*P. taeda*	4-Jul-2013
RB174	35.183	-81.963	YELLOW	SOUTH	Chesnee,SC	*P. taeda*	4-Jul-2013
RB175	35.183	-81.963	YELLOW	SOUTH	Chesnee,SC	*P. taeda*	4-Jul-2013
RB176	35.183	-81.963	YELLOW	SOUTH	Chesnee,SC	*P. taeda*	4-Jul-2013
RB177	35.183	-81.963	YELLOW	SOUTH	Chesnee,SC	*P. taeda*	4-Jul-2013
RB178	35.183	-81.963	YELLOW	SOUTH	Chesnee,SC	*P. taeda*	4-Jul-2013
RB179	35.183	-81.963	YELLOW	SOUTH	Chesnee,SC	*P. taeda*	4-Jul-2013
RB180	35.183	-81.963	YELLOW	SOUTH	Chesnee,SC	*P. taeda*	4-Jul-2013
RB181	35.183	-81.963	YELLOW	SOUTH	Chesnee,SC	*P. taeda*	4-Jul-2013
RB182	35.183	-81.963	YELLOW	SOUTH	Chesnee,SC	*P. taeda*	4-Jul-2013
RB183	35.183	-81.963	YELLOW	SOUTH	Chesnee,SC	*P. taeda*	4-Jul-2013
RB184	35.183	-81.963	YELLOW	SOUTH	Chesnee,SC	*P. taeda*	4-Jul-2013
RB185	35.183	-81.963	YELLOW	SOUTH	Chesnee,SC	*P. taeda*	4-Jul-2013
RB186	35.183	-81.963	YELLOW	SOUTH	Chesnee,SC	*P. taeda*	4-Jul-2013
RB187	35.183	-81.963	YELLOW	SOUTH	Chesnee,SC	*P. taeda*	4-Jul-2013
RB188	35.183	-81.963	YELLOW	SOUTH	Chesnee,SC	*P. taeda*	4-Jul-2013
RB189	35.183	-81.963	YELLOW	SOUTH	Chesnee,SC	*P. taeda*	4-Jul-2013
RB190	35.014	-82.716	YELLOW	SOUTH	Pickens, SC	*P. virginiana*	4-Jul-2013
RB197	34.409	-81.399	YELLOW	SOUTH	Pomaria, SC	*P. taeda*	5-Jul-2013
RB198	34.409	-81.399	YELLOW	SOUTH	Pomaria, SC	*P. taeda*	5-Jul-2013
RB199	34.409	-81.399	YELLOW	SOUTH	Pomaria, SC	*P. taeda*	5-Jul-2013
RB200	34.396	-81.402	YELLOW	SOUTH	Blair, SC	*P. taeda*	5-Jul-2013
RB201	34.396	-81.402	YELLOW	SOUTH	Blair, SC	*P. taeda*	5-Jul-2013
RB202	34.396	-81.402	YELLOW	SOUTH	Blair, SC	*P. taeda*	5-Jul-2013
RB204	33.765	-80.920	YELLOW	SOUTH	Columbia, SC	*P. taeda*	6-Jul-2013
RB205	33.765	-80.920	YELLOW	SOUTH	Columbia, SC	*P. taeda*	6-Jul-2013
RB211	34.018	-78.949	YELLOW	SOUTH	Loris, SC	*P. taeda (?)*	6-Jul-2013
RB212	34.034	-78.924	YELLOW	SOUTH	Loris, SC	*P. taeda*	6-Jul-2013

RB214	34.527	-78.745	YELLOW	NORTH	Bladenboro, NC	*P. taeda*	7-Jul-2013
RB215	35.184	-79.717	YELLOW	NORTH	Norman, NC	*P. taeda*	7-Jul-2013
RB217	35.515	-79.779	YELLOW	NORTH	Seagrove, NC	*P. taeda*	7-Jul-2013
RB220	36.497	-80.104	YELLOW	NORTH	Sandy Ridge, NC	*P. virginiana*	8-Jul-2013
RB221	36.497	-80.104	YELLOW	NORTH	Sandy Ridge, NC	*P. virginiana*	8-Jul-2013
RB222	36.497	-80.104	YELLOW	NORTH	Sandy Ridge, NC	*P. virginiana*	8-Jul-2013
RB223	36.802	-79.937	WHITE	CENTRAL	Bassett, VA	*P. echinata*	8-Jul-2013
RB224	36.973	-79.608	WHITE	CENTRAL	Penhook, VA	*P. rigida*	8-Jul-2013
RB225	36.973	-79.608	WHITE	CENTRAL	Penhook, VA	*P. rigida*	8-Jul-2013
RB226	36.940	-79.289	MIXED	CENTRAL	Gretna, VA	*P. rigida*	8-Jul-2013
RB227	37.319	-78.043	WHITE	CENTRAL	Amelia, VA	*P. rigida*	8-Jul-2013
RB228	37.319	-78.043	WHITE	CENTRAL	Amelia, VA	*P. rigida*	8-Jul-2013
RB229	37.319	-78.043	WHITE	CENTRAL	Amelia, VA	*P. rigida*	8-Jul-2013
RB230	37.356	-77.862	MIXED	CENTRAL	Amelia, VA	*P. rigida*	8-Jul-2013
RB233	43.759	-85.741	YELLOW	NORTH	Bitely, MI	*P. banksiana*	16-Jul-2013
RB234	43.759	-85.741	YELLOW	NORTH	Bitely, MI	*P. banksiana*	16-Jul-2013
RB235	43.759	-85.741	YELLOW	NORTH	Bitely, MI	*P. banksiana*	16-Jul-2013
RB236	43.759	-85.741	YELLOW	NORTH	Bitely, MI	*P. banksiana*	16-Jul-2013
RB237	43.786	-85.741	YELLOW	NORTH	Bitely, MI	*P. banksiana*	16-Jul-2013
RB238	43.791	-85.740	YELLOW	NORTH	Bitely, MI	*P. banksiana*	16-Jul-2013
RB239	43.791	-85.740	YELLOW	NORTH	Bitely, MI	*P. banksiana*	16-Jul-2013
RB240	43.793	-85.740	YELLOW	NORTH	Bitely, MI	*P. banksiana*	16-Jul-2013
RB241	43.793	-85.740	YELLOW	NORTH	Bitely, MI	*P. banksiana*	16-Jul-2013
RB242	43.793	-85.740	YELLOW	NORTH	Bitely, MI	*P. banksiana*	16-Jul-2013
RB243	43.793	-85.740	YELLOW	NORTH	Bitely, MI	*P. banksiana*	16-Jul-2013
RB244	43.793	-85.740	YELLOW	NORTH	Bitely, MI	*P. banksiana*	16-Jul-2013
RB245	44.600	-84.713	YELLOW	NORTH	Grayling, MI	*P. banksiana*	16-Jul-2013
RB246	44.600	-84.713	YELLOW	NORTH	Grayling, MI	*P. banksiana*	16-Jul-2013
RB247	44.600	-84.713	YELLOW	NORTH	Grayling, MI	*P. banksiana*	16-Jul-2013

RB248	44.583	-84.700	YELLOW	NORTH	Grayling, MI	*P. banksiana*	17-Jul-2013
RB249	44.658	-84.695	YELLOW	NORTH	Grayling, MI	*P. resinosa*	17-Jul-2013
RB250	44.658	-84.695	YELLOW	NORTH	Grayling, MI	*P. resinosa*	17-Jul-2013
RB251	44.658	-84.695	YELLOW	NORTH	Grayling, MI	*P. resinosa*	17-Jul-2013
RB252	44.658	-84.695	YELLOW	NORTH	Grayling, MI	*P. resinosa*	17-Jul-2013
RB253	44.658	-84.695	YELLOW	NORTH	Grayling, MI	*P. resinosa*	17-Jul-2013
RB254	44.658	-84.695	YELLOW	NORTH	Grayling, MI	*P. banksiana*	17-Jul-2013
RB255	44.658	-84.695	YELLOW	NORTH	Grayling, MI	*P. sylvestris*	17-Jul-2013
RB256	44.658	-84.695	YELLOW	NORTH	Grayling, MI	*P. banksiana*	17-Jul-2013
RB257	44.658	-84.695	YELLOW	NORTH	Grayling, MI	*P. banksiana*	17-Jul-2013
RB258	44.658	-84.695	YELLOW	NORTH	Grayling, MI	*P. banksiana*	17-Jul-2013
RB259	44.657	-84.696	YELLOW	NORTH	Grayling, MI	*P. banksiana*	17-Jul-2013
RB260	44.657	-84.696	YELLOW	NORTH	Grayling, MI	*P. banksiana*	17-Jul-2013
RB261	44.657	-84.696	YELLOW	NORTH	Grayling, MI	*P. banksiana*	17-Jul-2013
RB266	44.985	-88.449	YELLOW	NORTH	Suring, WI	*P. resinosa*	19-Jul-2013
RB267	44.985	-88.449	YELLOW	NORTH	Suring, WI	*P. resinosa*	19-Jul-2013
RB268	44.985	-88.449	YELLOW	NORTH	Suring, WI	*P. resinosa*	19-Jul-2013
RB269	44.985	-88.449	YELLOW	NORTH	Suring, WI	*P. resinosa*	19-Jul-2013
RB270	44.985	-88.449	YELLOW	NORTH	Suring, WI	*P. resinosa*	19-Jul-2013
RB271	44.985	-88.449	YELLOW	NORTH	Suring, WI	*P. resinosa*	19-Jul-2013
RB272	44.985	-88.449	YELLOW	NORTH	Suring, WI	*P. resinosa*	19-Jul-2013
RB273	44.985	-88.449	YELLOW	NORTH	Suring, WI	*P. resinosa*	19-Jul-2013
RB274	44.985	-88.449	YELLOW	NORTH	Suring, WI	*P. resinosa*	19-Jul-2013
RB275	44.054	-89.806	YELLOW	NORTH	Arkdale, WI	*P. banksiana*	20-Jul-2013
RB278	44.112	-90.117	YELLOW	NORTH	Necedah, WI	*P. banksiana*	20-Jul-2013
RB279	44.115	-90.118	YELLOW	NORTH	Necedah, WI	*P. banksiana*	20-Jul-2013
RB280	44.115	-90.118	YELLOW	NORTH	Necedah, WI	*P. banksiana*	20-Jul-2013
RB281	44.115	-90.118	YELLOW	NORTH	Necedah, WI	*P. banksiana*	20-Jul-2013
RB282	44.115	-90.118	YELLOW	NORTH	Necedah, WI	*P. banksiana*	20-Jul-2013

RB283	44.136	-90.128	YELLOW	NORTH	Necedah, WI	*P. banksiana*	20-Jul-2013
RB284	44.207	-90.136	YELLOW	NORTH	Necedah, WI	*P. banksiana*	20-Jul-2013
RB285	44.207	-90.136	YELLOW	NORTH	Necedah, WI	*P. banksiana*	20-Jul-2013
RB286	39.631	-77.963	YELLOW	CENTRAL	Clear Spring, MD	*P. virginiana*	25-Jul-2013
RB287	39.631	-77.963	YELLOW	CENTRAL	Clear Spring, MD	*P. virginiana*	25-Jul-2013
RB288	39.631	-77.963	YELLOW	CENTRAL	Clear Spring, MD	*P. virginiana*	25-Jul-2013
RB289	39.631	-77.963	YELLOW	CENTRAL	Clear Spring, MD	*P. virginiana*	25-Jul-2013
RB290	40.387	-74.335	WHITE	CENTRAL	Old Bridge, NJ	*P. rigida*	26-Jul-2013
RB291	40.427	-74.303	MIXED	CENTRAL	Old Bridge NJ	*P. sylvestris*	26-Jul-2013
RB292	40.427	-74.303	WHITE	CENTRAL	Old Bridge, NJ	*P. sylvestris*	26-Jul-2013
RB293	43.559	-73.733	YELLOW	CENTRAL/NORTH	Warrensburg, NY	*P. sylvestris*	28-Jul-2013
RB294	43.559	-73.733	YELLOW	CENTRAL/NORTH	Warrensburg, NY	*P. sylvestris*	28-Jul-2013
RB295	43.559	-73.733	YELLOW	CENTRAL/NORTH	Warrensburg, NY	*P. sylvestris*	28-Jul-2013
RB296	43.559	-73.733	YELLOW	CENTRAL/NORTH	Warrensburg, NY	*P. sylvestris*	28-Jul-2013
RB297	43.559	-73.733	YELLOW	CENTRAL/NORTH	Warrensburg, NY	*P. sylvestris*	28-Jul-2013
RB298	43.559	-73.733	YELLOW	CENTRAL/NORTH	Warrensburg, NY	*P. strobus*	28-Jul-2013
RB299	44.000	-73.718	YELLOW	CENTRAL/NORTH	Warrensburg, NY	*P. strobus*	28-Jul-2013
RB300	44.000	-73.718	YELLOW	CENTRAL/NORTH	Warrensburg, NY	*P. strobus*	28-Jul-2013
RB301	44.000	-73.718	YELLOW	CENTRAL/NORTH	Warrensburg, NY	*P. resinosa*	28-Jul-2013
RB302	44.000	-73.718	YELLOW	CENTRAL/NORTH	Warrensburg, NY	*P. resinosa*	28-Jul-2013
RB303	44.016	-73.704	YELLOW	CENTRAL/NORTH	North Hudson, NY	*P. resinosa*	28-Jul-2013
RB304	43.819	-71.205	YELLOW	CENTRAL	Ossippee, NH	*P. rigida*	29-Jul-2013
RB305	43.819	-71.205	YELLOW	CENTRAL	Ossippee, NH	*P. rigida*	29-Jul-2013
RB306	43.819	-71.205	YELLOW	CENTRAL	Ossippee, NH	*P. rigida*	29-Jul-2013
RB307	43.819	-71.205	YELLOW	CENTRAL	Ossippee, NH	*P. rigida*	29-Jul-2013
RB308	43.676	-71.082	YELLOW	CENTRAL	Ossippee, NH	*P. rigida*	29-Jul-2013
RB309	30.239	-82.299	YELLOW	SOUTH	Sanderson, FL	*P. ellotti*	6-Aug-2013
RB310	29.508	-81.860	YELLOW	SOUTH	Fort McCoy, FL	*P. palustris*	7-Aug-2013
RB311	29.508	-81.860	YELLOW	SOUTH	Fort McCoy, FL	*P. palustris*	7-Aug-2013

RB312	29.508	-81.860	YELLOW	SOUTH	Fort McCoy, FL	*P. palustris*	7-Aug-2013
RB313	29.508	-81.860	YELLOW	SOUTH	Fort McCoy, FL	*P. palustris*	7-Aug-2013
RB314	29.508	-81.860	YELLOW	SOUTH	Fort McCoy, FL	*P. palustris*	7-Aug-2013
RB315	29.508	-81.860	YELLOW	SOUTH	Fort McCoy, FL	*P. palustris*	7-Aug-2013
RB316	29.508	-81.860	YELLOW	SOUTH	Fort McCoy, FL	*P. palustris*	7-Aug-2013
RB317	29.591	-82.362	YELLOW	SOUTH	Arrendondo, FL	*P. palustris*	7-Aug-2013
RB318	30.255	-84.685	YELLOW	SOUTH	Sopchoppy,FL	*P. palustris*	8-Aug-2013
RB319	30.255	-84.685	YELLOW	SOUTH	Sopchoppy,FL	*P. palustris*	8-Aug-2013
RB320	30.255	-84.685	YELLOW	SOUTH	Sopchoppy,FL	*P. palustris*	8-Aug-2013
RB321	30.255	-84.685	YELLOW	SOUTH	Sopchoppy,FL	*P. palustris*	8-Aug-2013
RB322	30.255	-84.685	YELLOW	SOUTH	Sopchoppy,FL	*P. palustris*	8-Aug-2013
RB323	30.255	-84.685	YELLOW	SOUTH	Sopchoppy,FL	*P. palustris*	8-Aug-2013
RB325	30.269	-84.362	YELLOW	SOUTH	Crawfordville, FL	*P. elliottii*	8-Aug-2013
RB326	30.255	-84.362	YELLOW	SOUTH	Crawfordville, FL	*P. elliottii*	8-Aug-2013
RB327	30.255	-84.362	YELLOW	SOUTH	Crawfordville, FL	*P. elliottii*	8-Aug-2013
RB328	30.255	-84.362	YELLOW	SOUTH	Crawfordville, FL	*P. elliottii*	8-Aug-2013
RB329	30.255	-84.362	YELLOW	SOUTH	Crawfordville, FL	*P. elliottii*	8-Aug-2013
RB330	30.315	-84.340	YELLOW	SOUTH	Tallahassee, FL	*P. palustris*	8-Aug-2013
RB331	30.333	-84.323	YELLOW	SOUTH	Woodville, FL	*P. palustris*	8-Aug-2013
RB332	30.333	-84.323	YELLOW	SOUTH	Woodville, FL	*P. palustris*	8-Aug-2013
RB333	31.090	-86.567	YELLOW	SOUTH	Dixie, AL	*P. elliottii*	9-Aug-2013
RB334	31.090	-86.567	YELLOW	SOUTH	Dixie, AL	*P. elliottii*	9-Aug-2013
RB335	38.014	-84.504	YELLOW	CENTRAL	Lexington KY	*P. echinata*	22-Aug-2013
RB336	38.014	-84.504	YELLOW	CENTRAL	Lexington KY	*P. echinata*	22-Aug-2013
RB337	38.014	-84.504	YELLOW	CENTRAL	Lexington KY	*P. virginiana*	22-Aug-2013
RB338	38.014	-84.504	YELLOW	CENTRAL	Lexington KY	*P. echinata*	22-Aug-2013
RB339	38.014	-84.504	YELLOW	CENTRAL	Lexington KY	*P. echinata*	22-Aug-2013
RB340	38.014	-84.504	YELLOW	CENTRAL	Lexington KY	*P. echinata*	22-Aug-2013
RB341	38.014	-84.504	YELLOW	CENTRAL	Lexington KY	*P. virginiana*	22-Aug-2013

RB342	38.014	-84.504	YELLOW	CENTRAL	Lexington KY	*P. echinata*	9-Sep-2013
RB343	38.014	-84.504	YELLOW	CENTRAL	Lexington KY	*P. rigida*	9-Sep-2013
RB344	38.014	-84.504	YELLOW	CENTRAL	Lexington KY	*P. rigida*	9-Sep-2013
RB345	38.014	-84.504	YELLOW	CENTRAL	Lexington KY	*P. rigida*	9-Sep-2013
RB346	38.014	-84.504	YELLOW	CENTRAL	Lexington KY	*P. virginiana*	9-Sep-2013
RB347	38.014	-84.504	YELLOW	CENTRAL	Lexington KY	*P. virginiana*	9-Sep-2013
RB348	38.014	-84.504	YELLOW	CENTRAL	Lexington KY	*P. virginiana*	9-Sep-2013
RB349	38.014	-84.504	YELLOW	CENTRAL	Lexington KY	*P. echinata*	9-Sep-2013
RB350	38.014	-84.504	YELLOW	CENTRAL	Lexington KY	*P. echinata*	9-Sep-2013
RB351	38.014	-84.504	YELLOW	CENTRAL	Lexington KY	*P. echinata*	9-Sep-2013
RB352	38.014	-84.504	YELLOW	CENTRAL	Lexington KY	*P. echinata*	9-Sep-2013
RB353	38.014	-84.504	YELLOW	CENTRAL	Lexington KY	*P. echinata*	9-Sep-2013
RB354	38.014	-84.504	YELLOW	CENTRAL	Lexington KY	*P. virginiana*	9-Sep-2013
RB355	38.014	-84.504	YELLOW	CENTRAL	Lexington KY	*P. virginiana*	9-Sep-2013
RB356	38.014	-84.504	YELLOW	CENTRAL	Lexington KY	*P. virginiana*	9-Sep-2013
RB357	38.014	-84.504	YELLOW	CENTRAL	Lexington KY	*P. virginiana*	9-Sep-2013
RB358	38.014	-84.504	YELLOW	CENTRAL	Lexington KY	*P. virginiana*	9-Sep-2013
RB359	38.014	-84.504	YELLOW	CENTRAL	Lexington KY	*P. virginiana*	9-Sep-2013
RB360	38.014	-84.504	YELLOW	CENTRAL	Lexington KY	*P. echinata*	9-Sep-2013
RB361	37.984	-84.418	YELLOW	CENTRAL	Lexington KY	*P. taeda*	10-Sep-2013
RB368	40.368	-74.302	WHITE	CENTRAL	Old Bridge, NJ	*P. rigida*	20-Jul-2014
RB369	40.368	-74.302	WHITE	CENTRAL	Old Bridge, NJ	*P. rigida*	20-Jul-2014
RB370	38.033	-84.507	YELLOW	CENTRAL	Lexington KY	*P. mugho*	3-Sep-2014
RB371	45.493	-77.597	YELLOW	NORTH	Wilno, ON	*P. resinosa*	19-Aug-2014
RB372	45.512	-77.447	YELLOW	NORTH	Killaloe, ON	*P. resinosa*	19-Aug-2014
RB373	46.471	-82.663	YELLOW	NORTH	Elliot Lake, ON	*P. resinosa*	22-Aug-2014
RB374	46.471	-82.663	YELLOW	NORTH	Elliot Lake, ON	*P. resinosa*	22-Aug-2014
RB375	46.471	-82.663	YELLOW	NORTH	Elliot Lake, ON	*P. resinosa*	22-Aug-2014
RB376	46.471	-82.663	YELLOW	NORTH	Elliot Lake, ON	*P. resinosa*	22-Aug-2014

RB377	46.471	-82.663	YELLOW	NORTH	Elliot Lake, ON	*P. resinosa*	22-Aug-2014
RB378	46.471	-82.663	YELLOW	NORTH	Elliot Lake, ON	*P. resinosa*	22-Aug-2014
RB379	46.439	-83.227	YELLOW	NORTH	Iron Bridge, ON	*P. resinosa*	22-Aug-2014
RB380	43.768	-85.740	YELLOW	NORTH	Brohman, MI	*P. banksiana*	15-Jul-2015
RB381	43.769	-85.741	YELLOW	NORTH	Brohman, MI	*P. banksiana*	15-Jul-2015
RB382	43.770	-85.742	YELLOW	NORTH	Bitely, MI	*P. banksiana*	15-Jul-2015
RB383	43.770	-85.742	YELLOW	NORTH	Bitely, MI	*P. banksiana*	15-Jul-2015
RB384	43.770	-85.742	YELLOW	NORTH	Bitely, MI	*P. banksiana*	15-Jul-2015
RB385	43.770	-85.742	YELLOW	NORTH	Bitely, MI	*P. banksiana*	15-Jul-2015
RB386	44.657	-84.696	YELLOW	NORTH	Grayling, MI	*P. banksiana*	16-Jul-2015
RB387	44.657	-84.696	YELLOW	NORTH	Grayling, MI	*P. banksiana*	16-Jul-2015
RB388	44.657	-84.696	YELLOW	NORTH	Grayling, MI	*P. banksiana*	16-Jul-2015
RB389	44.983	-88.448	YELLOW	NORTH	Suring, WI	*P. resinosa*	17-Jul-2015
RB390	44.983	-88.448	YELLOW	NORTH	Suring, WI	*P. resinosa*	17-Jul-2015
RB391	44.983	-88.448	YELLOW	NORTH	Suring, WI	*P. resinosa*	17-Jul-2015
RB392	44.983	-88.448	YELLOW	NORTH	Suring, WI	*P. resinosa*	17-Jul-2015
RB393	44.983	-88.448	YELLOW	NORTH	Suring, WI	*P. resinosa*	17-Jul-2015
RB394	44.983	-88.448	YELLOW	NORTH	Suring, WI	*P. resinosa*	17-Jul-2015
RB395	44.983	-88.448	YELLOW	NORTH	Suring, WI	*P. resinosa*	17-Jul-2015
RB396	44.156	-90.132	YELLOW	NORTH	Necedah, WI	*P. banksiana*	17-Jul-2015
RB397	44.156	-90.132	YELLOW	NORTH	Necedah, WI	*P. banksiana*	17-Jul-2015
RB398	44.156	-90.132	YELLOW	NORTH	Necedah, WI	*P. banksiana*	17-Jul-2015
RB399	44.156	-90.132	YELLOW	NORTH	Necedah, WI	*P. banksiana*	17-Jul-2015
RB400	44.156	-90.132	YELLOW	NORTH	Necedah, WI	*P. banksiana*	17-Jul-2015
RB401	44.036	-90.082	YELLOW	NORTH	Necedah, WI	*P. banksiana*	17-Jul-2015
RB402	44.036	-90.082	YELLOW	NORTH	Necedah, WI	*P. banksiana*	17-Jul-2015
RB403	44.154	-90.132	YELLOW	NORTH	Necedah, WI	*P. banksiana*	17-Jul-2015
RB404	38.186	-83.557	YELLOW	CENTRAL	Morehead, KY	*P. echinata*	10-Aug-2015
RB405	45.949	-86.261	YELLOW	NORTH	Manistique, MI	*P. resinosa*	16-Aug-2015

RB406	45.949	-86.261	YELLOW	NORTH	Manistique, MI	*P. resinosa*	16-Aug-2015
RB407	45.918	-86.313	YELLOW	NORTH	Thompson Township, MI	*P. banksiana*	16-Aug-2015
RB408	45.918	-86.313	YELLOW	NORTH	Thompson Township, MI	*P. banksiana*	16-Aug-2015
RB409	45.926	-86.294	YELLOW	NORTH	Thompson Township, MI	*P. resinosa*	16-Aug-2015
RB410	45.926	-86.294	YELLOW	NORTH	Thompson Township, MI	*P. resinosa*	16-Aug-2015
RB411	45.926	-86.294	YELLOW	NORTH	Thompson Township, MI	*P. resinosa*	16-Aug-2015
RB412	45.926	-86.294	YELLOW	NORTH	Thompson Township, MI	*P. resinosa*	16-Aug-2015
RB413	45.926	-86.294	YELLOW	NORTH	Thompson Township, MI	*P. resinosa*	16-Aug-2015
RB414	45.926	-86.294	YELLOW	NORTH	Thompson Township, MI	*P. resinosa*	16-Aug-2015
RB415	45.926	-86.294	YELLOW	NORTH	Thompson Township, MI	*P. resinosa*	16-Aug-2015
RB416	44.983	-88.448	YELLOW	NORTH	Suring, WI	*P. resinosa*	17-Aug-2015
RB417	44.983	-88.448	YELLOW	NORTH	Suring, WI	*P. resinosa*	17-Aug-2015
RB418	44.983	-88.448	YELLOW	NORTH	Suring, WI	*P. resinosa*	17-Aug-2015
RB419	44.983	-88.448	YELLOW	NORTH	Suring, WI	*P. resinosa*	17-Aug-2015
RB420	44.983	-88.448	YELLOW	NORTH	Suring, WI	*P. resinosa*	17-Aug-2015
RB421	44.983	-88.448	YELLOW	NORTH	Suring, WI	*P. resinosa*	17-Aug-2015
RB425	44.862	-89.637	YELLOW	NORTH	Rothschild, WI	*P. banksiana*	9-Jul-2016
RB426	44.862	-89.637	YELLOW	NORTH	Rothschild, WI	*P. banksiana*	9-Jul-2016
RB427	44.862	-89.637	YELLOW	NORTH	Rothschild, WI	*P. banksiana*	9-Jul-2016
RB428	44.862	-89.637	YELLOW	NORTH	Rothschild, WI	*P. banksiana*	9-Jul-2016
RB429	44.862	-89.637	YELLOW	NORTH	Rothschild, WI	*P. banksiana*	9-Jul-2016
RB430	44.862	-89.637	YELLOW	NORTH	Rothschild, WI	*P. banksiana*	9-Jul-2016
RB431	44.844	-89.691	YELLOW	NORTH	Mosinee, WI	*P. resinosa*	9-Jul-2016
RB432	44.844	-89.691	YELLOW	NORTH	Mosinee, WI	*P. resinosa*	9-Jul-2016
RB433	44.844	-89.691	YELLOW	NORTH	Mosinee, WI	*P. banksiana*	9-Jul-2016
RB434	44.844	-89.691	YELLOW	NORTH	Mosinee, WI	*P. banksiana*	9-Jul-2016
RB435	44.844	-89.691	YELLOW	NORTH	Mosinee, WI	*P. banksiana*	9-Jul-2016
RB437	44.026	-89.705	YELLOW	NORTH	Friendship, WI	*P. banksiana*	9-Jul-2016
RB438	44.026	-89.705	YELLOW	NORTH	Friendship, WI	*P. banksiana*	9-Jul-2016

RB439	44.026	-89.705	YELLOW	NORTH	Friendship, WI	*P. banksiana*	9-Jul-2016
RB440	44.026	-89.705	YELLOW	NORTH	Friendship, WI	*P. banksiana*	9-Jul-2016
RB441	44.036	-90.082	YELLOW	NORTH	Necedah, WI	*P. banksiana*	9-Jul-2016
RB442	44.036	-90.082	YELLOW	NORTH	Necedah, WI	*P. banksiana*	9-Jul-2016
RB443	44.036	-90.082	YELLOW	NORTH	Necedah, WI	*P. banksiana*	9-Jul-2016
RB444	44.731	-84.749	YELLOW	NORTH	Frederic, MI	*P. resinosa*	23-Jul-2016
RB445	44.731	-84.749	YELLOW	NORTH	Frederic, MI	*P. resinosa*	23-Jul-2016
RB446	44.731	-84.749	YELLOW	NORTH	Frederic, MI	*P. resinosa*	23-Jul-2016
RB447	44.731	-84.749	YELLOW	NORTH	Frederic, MI	*P. resinosa*	23-Jul-2016
RB448	44.731	-84.749	YELLOW	NORTH	Frederic, MI	*P. resinosa*	23-Jul-2016
RB449	44.731	-84.749	YELLOW	NORTH	Frederic, MI	*P. resinosa*	23-Jul-2016
RB450	44.731	-84.749	YELLOW	NORTH	Frederic, MI	*P. resinosa*	23-Jul-2016
RB451	44.720	-84.745	YELLOW	NORTH	Frederic, MI	*P. resinosa*	23-Jul-2016
RB452	44.720	-84.745	YELLOW	NORTH	Frederic, MI	*P. resinosa*	23-Jul-2016
RB453	44.720	-84.745	YELLOW	NORTH	Frederic, MI	*P. resinosa*	23-Jul-2016
RB454	44.731	-84.749	YELLOW	NORTH	Frederic, MI	*P. resinosa*	23-Jul-2016
RB455	44.731	-84.749	YELLOW	NORTH	Frederic, MI	*P. resinosa*	23-Jul-2016
RB456	44.731	-84.749	YELLOW	NORTH	Frederic, MI	*P. resinosa*	23-Jul-2016
RB457	44.731	-84.749	YELLOW	NORTH	Frederic, MI	*P. resinosa*	23-Jul-2016
RB458	44.731	-84.749	YELLOW	NORTH	Frederic, MI	*P. resinosa*	23-Jul-2016
RB459	44.731	-84.749	YELLOW	NORTH	Frederic, MI	*P. resinosa*	23-Jul-2016
RB460	44.731	-84.749	YELLOW	NORTH	Frederic, MI	*P. resinosa*	23-Jul-2016
RB461	44.731	-84.749	YELLOW	NORTH	Frederic, MI	*P. resinosa*	23-Jul-2016
RB462	44.731	-84.749	YELLOW	NORTH	Frederic, MI	*P. banksiana*	23-Jul-2016
RB463	44.731	-84.749	YELLOW	NORTH	Frederic, MI	*P. banksiana*	23-Jul-2016
RB464	44.731	-84.749	YELLOW	NORTH	Frederic, MI	*P. banksiana*	23-Jul-2016
RB465	44.731	-84.749	YELLOW	NORTH	Frederic, MI	*P. banksiana*	23-Jul-2016
RB466	44.731	-84.749	YELLOW	NORTH	Frederic, MI	*P. banksiana*	23-Jul-2016
RB467	44.731	-84.749	YELLOW	NORTH	Frederic, MI	*P. banksiana*	23-Jul-2016

RB468	44.731	-84.749	YELLOW	NORTH	Frederic, MI	*P. banksiana*	23-Jul-2016
RB469	44.731	-84.749	YELLOW	NORTH	Frederic, MI	*P. banksiana*	23-Jul-2016
RB470	44.731	-84.749	YELLOW	NORTH	Frederic, MI	*P. banksiana*	23-Jul-2016
RB471	44.731	-84.749	YELLOW	NORTH	Frederic, MI	*P. banksiana*	23-Jul-2016
RB472	44.731	-84.749	YELLOW	NORTH	Frederic, MI	*P. banksiana*	23-Jul-2016
RB473	44.731	-84.749	YELLOW	NORTH	Frederic, MI	*P. banksiana*	23-Jul-2016
RB474	44.731	-84.749	YELLOW	NORTH	Frederic, MI	*P. banksiana*	23-Jul-2016
RB475	44.731	-84.749	YELLOW	NORTH	Frederic, MI	*P. banksiana*	23-Jul-2016
RB476	44.731	-84.749	YELLOW	NORTH	Frederic, MI	*P. banksiana*	23-Jul-2016
RB477	44.731	-84.749	YELLOW	NORTH	Frederic, MI	*P. banksiana*	23-Jul-2016
RB478	44.731	-84.749	YELLOW	NORTH	Frederic, MI	*P. banksiana*	23-Jul-2016
RB479	44.731	-84.749	YELLOW	NORTH	Frederic, MI	*P. banksiana*	23-Jul-2016
RB480	46.096	-85.394	YELLOW	NORTH	Naubinway, MI	*P. resinosa*	23-Jul-2016
RB481	46.096	-85.394	YELLOW	NORTH	Naubinway, MI	*P. banksiana*	23-Jul-2016
RB482	46.096	-85.394	YELLOW	NORTH	Naubinway, MI	*P. banksiana*	23-Jul-2016
RB483	46.096	-85.394	YELLOW	NORTH	Naubinway, MI	*P. banksiana*	23-Jul-2016
RB484	44.731	-84.749	YELLOW	NORTH	Frederic, MI	*P. resinosa*	23-Jul-2016
RB485	44.731	-84.749	YELLOW	NORTH	Frederic, MI	*P. resinosa*	23-Jul-2016
