# Supplementary material for: The impact of life stage and pigment source on the evolution of novel warning signal traits
Source: Evolution. 2022 Feb 10;76(3):554–72. doi: 10.1111/evo.14443 (PMC9304160; doi:10.1111/evo.14443)
Supplement: Supplementary file 7 — Table S2. Human‐sorted white and yellow Neodiprion lecontei larvae reflected underlying differences in color. Similarly color of frozen white and yellow N. lecontei larvae recapitulate differences between fresh larvae. [file EVO-76-554-s009.docx]

**Table S2. Human-sorted white and yellow *Neodiprion lecontei* larvae reflected underlying differences in color. Similarly color of frozen white and yellow *N. lecontei* larvae recapitulate differences between fresh larvae.** We recorded reflectance spectra from 10 frozen larvae of each color (white and yellow) and from 10 living, CO_2_-immobilized larvae of each color. For each larva, we recorded nine spectra across the dorsal, lateral, and ventral surfaces (three spectra per surface) and computed an average S1B value (summary statistic that correlates with carotenoid content) as described in the main text. We then performed a two-way ANOVA in R with human-designated color category (white or yellow) and larval treatment (fresh or frozen) as main effects, plus an interaction term, followed by a Tukey’s Honest Significant Difference test with FDR correction for multiple testing. Significant adjusted p-values (<0.05) are bolded. A significant “Category” term indicates that there are significant differences in color between larvae that were sorted as yellow or white. Although freezing also has an impact on larval color (significant treatment effect), the larval color morphs do not respond differently to freezing (the interaction term is not significant). Also note that white and yellow larvae differ significantly in color (and in the same direction) regardless of whether both are fresh or both are frozen (Fresh yellow vs. Fresh white and Frozen yellow vs. Frozen white comparisons).

*Two-way ANOVA table*

|  | DF | Sum Sq. | Mean Sq. | F-value | P-value |
| --- | --- | --- | --- | --- | --- |
| Category (White or Yellow) | 1 | 0.0241 | 0.0241 | 8.39 | **0.00637** |
| Treatment (Fresh or Frozen) | 1 | 0.109 | 0.109 | 38.0 | **4.16e-07** |
| Category:Treatment | 1 | 0.00452 | 0.00452 | 1.57 | 0.218 |
| Residuals | 36 | 0.103 | 0.00287 |  |  |

*Tukey HSD test*

| Comparison | Diff | Lwr | Upr | P. adj. |
| --- | --- | --- | --- | --- |
| Frozen:White-Fresh:White | -0.0279 | -0.0924 | 0.0367 | 0.654 |
| Fresh:Yellow-Fresh:White | -0.0833 | -0.148 | -0.0187 | **0.00708** |
| Frozen:Yellow-Fresh:White | -0.154 | -0.218 | -0.0891 | **0.0000012** |
| Fresh:Yellow-Frozen:White | -0.0554 | -0.120 | 0.00914 | 0.114 |
| Frozen:Yellow-Frozen:White | -0.126 | -0.190 | -0.0612 | **0.0000405** |
| Frozen:Yellow-Fresh:Yellow | -0.0704 | -0.135 | -0.00580 | **0.0282** |
