# Supplementary material for: The impact of life stage and pigment source on the evolution of novel warning signal traits
Source: Evolution. 2022 Feb 10;76(3):554–72. doi: 10.1111/evo.14443 (PMC9304160; doi:10.1111/evo.14443)
Supplement: Supplementary file 8 — Table S3. Sampling locations for all individuals included in population genomic analyses. [file EVO-76-554-s004.docx]

**Table S3. Sampling locations for all individuals included in population genomic analyses.** Specimens noted with a dagger (†) were adult females; all other specimens were larvae of unknown sex, but confirmed to be diploid via heterozygosity estimates. Full sequences for the adapter-ligated barcodes and Illumina indexes are listed in Tables S3 and S4, respectively.

| **ID** | **Location** | **Latitude** | **Longitude** | **Host** | **Color** | **Index** | **Barcode** |
| --- | --- | --- | --- | --- | --- | --- | --- |
| LL004_01† | Lexington, KY | 38.014 | -84.504 | *P. echinata* | Yellow | ATCACGAT | TGGCACAGA |
| LL005_01† | Lexington, KY | 38.014 | -84.504 | *P. rigida* | Yellow | TGACCAAT | TGGCAACAGA |
| LL010 | Bronston, KY | 36.9279 | -84.6193 | *P. virginiana* | Yellow | GTGAAACG | AACTGG |
| LL011 | London, KY | 37.07133 | -84.2114 | *P. echinata* | Yellow | AGTCAACA | CTAAGCA |
| LL013 | London, KY | 37.07133 | -84.2114 | *P. echinata* | Yellow | TGACCAAT | ACAACT |
| LL014 | London, KY | 37.07133 | -84.2114 | *P. echinata* | Yellow | CCGTCCCG | ACTGCGAT |
| LL015_01 | London, KY | 37.07133 | -84.2114 | *P. echinata* | Yellow | ATCACGAT | TCAGAGAT |
| LL042 | Ford, VA | 37.24904 | -77.7253 | *P. echinata* | White | TTAGGCAT | GCAAGCCAT |
| LL058_2 | Lexington, KY | 38.014 | -84.504 | *P. virginiana* | Yellow | AGTCAACA | ACGGTACT |
| LL062_1 | Lexington, KY | 38.014 | -84.504 | *P. echinata* | Yellow | CGATGTAT | TGACGCCA |
| LL064 | Lexington, KY | 38.014 | -84.504 | *P. rigida* | Yellow | TTAGGCAT | ACGGTACT |
| LL070_1 | Lexington, KY | 38.014 | -84.504 | *P. rigida* | Yellow | CGATGTAT | ATATCGCCA |
| LL073_2R | Lexington, KY | 38.014 | -84.504 | *P. echinata* | Yellow | CGATGTAT | AAGACGCT |
| LL074_5 | Lexington, KY | 38.014 | -84.504 | *P. echinata* | Yellow | ATCACGAT | ACAACCAACT |
| LL084 | West Yarmouth, MA | 41.64545 | -70.2302 | *P. rigida* | White | ATCACGAT | GAGCGACAT |
| LL084_02 | West Yarmouth, MA | 41.64545 | -70.2302 | *P. rigida* | White | CCGTCCCG | GAGCGACAT |
| LL084_03 | West Yarmouth, MA | 41.64545 | -70.2302 | *P. rigida* | White | GTGAAACG | CTCGCGG |
| LL102 | Lexington, KY | 38.014 | -84.504 | *P. rigida* | Yellow | TTAGGCAT | AACGCACATT |
| LL137 | Lexington, KY | 38.014 | -84.504 | *P. virginiana* | Yellow | CGATGTAT | CTCGCGG |
| LL142_02 | Crossville, TN | 35.98003 | -85.0152 | *P. virginiana* | Yellow | AGTCAACA | CAACCACACA |
| LL144_01 | Crossville, TN | 35.98003 | -85.0152 | *P. virginiana* | Yellow | GTCCGCAC | TCAGAGAT |
| LL180_02 | Goshen, KY | 38.40235 | -85.5859 | *P. echinata* | Yellow | TGACCAAT | TATGT |
| LL181 | Goshen, KY | 38.40235 | -85.5859 | *P. echinata* | Yellow | GTGAAACG | CACCA |
| LL194_02 | Stanton, KY | 37.80602 | -83.6779 | *P. virginiana* | Yellow | TGACCAAT | TGCTT |
| LL195_02 | Stanton, KY | 37.80602 | -83.6779 | *P. virginiana* | Yellow | GTCCGCAC | CTTGA |
| RB020Db | London, KY | 37.066 | -84.159 | *P. echinata* | Yellow | GTCCGCAC | ATTAT |
| RB028_01† | Egg Harbor, NJ | 39.69028 | -74.593 | *P. rigida* | White | GTCCGCAC | CAACCACACA |
| RB076_01 | Lexington, KY | 38.014 | -84.504 | *P. virginiana* | Yellow | GTGAAACG | TGACGCCA |
| RB107_01 | Mountain Grove, VA | 38.212 | -79.719 | *P. rigida* | White | GTGAAACG | CCGAACA |
| RB108_01 | Deer Run, WV | 38.678 | -79.399 | *P. rigida* | White | GTGAAACG | CGTCGCCACT |
| RB110.01† | Browns Mill, NJ | 39.934 | -74.533 | *P. rigida* | White | AGTCAACA | ACAACCAACT |
| RB112_01 | Tuckerton, NJ | 39.621 | -74.428 | *P. rigida* | White | TGACCAAT | CCTTGCCATT |
| RB118_01 | Brandywine, WV | 38.592 | -79.172 | *P. virginiana* | White | CGATGTAT | GCAAGCCAT |
| RB119_01 | Buena Vista, VA | 37.713 | -79.367 | *P. virginiana* | White | TGACCAAT | AACGCACATT |
| RB126_01 | Lexington, KY | 38.014 | -84.504 | *P. virginiana* | Yellow | GTGAAACG | AACGTGCCT |
| RB164 | Tyron, NC | 35.28019 | -82.1175 | *P. virginiana* | Yellow | AGTCAACA | TATTCGCAT |
| RB165 | Tyron, NC | 35.28014 | -82.1179 | *P. virginiana* | Yellow | TTAGGCAT | CTCGCGG |
| RB167_01 | Chesnee, SC | 35.18269 | -81.963 | *P. virginiana* | Yellow | ATCACGAT | GGCTTA |
| RB190 | Pickens, SC | 35.01364 | -82.7156 | *P. virginiana* | Yellow | CCGTCCCG | ACGGTACT |
| RB220 | Sandy Ridge, NC | 36.49728 | -80.1035 | *P. virginiana* | Yellow | AGTCAACA | GGTGCACATT |
| RB221 | Sandy Ridge, NC | 36.49728 | -80.1035 | *P. virginiana* | Yellow | GTCCGCAC | TCACTG |
| RB222 | Sandy Ridge, NC | 36.49728 | -80.1035 | *P. virginiana* | Yellow | ATCACGAT | GCGTCCT |
| RB223 | Bassett, VA | 36.80192 | -79.9371 | *P. echinata* | Mixed | ATCACGAT | ACGGTACT |
| RB224 | Penhook, VA | 36.97347 | -79.6076 | *P. rigida* | White | ATCACGAT | AAGACGCT |
| RB225 | Penhook, VA | 36.97347 | -79.6076 | *P. rigida* | White | AGTCAACA | GCAAGCCAT |
| RB226_old | Gretna, VA | 36.93961 | -79.2893 | *P. rigida* | Mixed | ATCACGAT | ACCAGGA |
| RB227 | Amelia, VA | 37.31906 | -78.0428 | *P. rigida* | White | AGTCAACA | CTCGCGG |
| RB228_new | Amelia, VA | 37.31906 | -78.0428 | *P. rigida* | White | ATCACGAT | CTCGCGG |
| RB229 | Amelia, VA | 37.31906 | -78.0428 | *P. rigida* | White | CCGTCCCG | ATGAGCAA |
| RB286 | Clear Springs, MD | 39.63111 | -77.9631 | *P. virginiana* | Yellow | GTCCGCAC | TAGCGGAT |
| RB287 | Clear Springs, MD | 39.63111 | -77.9631 | *P. virginiana* | Yellow | AGTCAACA | AACGCACATT |
| RB288_02 | Clear Springs, MD | 39.63111 | -77.9631 | *P. virginiana* | Yellow | TGACCAAT | GGTGT |
| RB289 | Clear Springs, MD | 39.63111 | -77.9631 | *P. virginiana* | Yellow | GTGAAACG | ATAGAT |
| RB290 | Old Bridge, NJ | 40.38742 | -74.3351 | *P. rigida* | White | TGACCAAT | CTTGA |
| RB304 | Ossippee, NH | 43.81939 | -71.2052 | *P. rigida* | Yellow | GTCCGCAC | ATGAGCAA |
| RB305 | Ossippee, NH | 43.81939 | -71.2052 | *P. rigida* | Yellow | AGTCAACA | CTCTCGCAT |
| RB306 | Ossippee, NH | 43.81939 | -71.2052 | *P. rigida* | Yellow | CGATGTAT | TATTCGCAT |
| RB307 | Ossippee, NH | 43.81939 | -71.2052 | *P. rigida* | Yellow | TGACCAAT | AACGTGCCT |
| RB308 | Ossippee, NH | 43.67597 | -71.0816 | *P. rigida* | Yellow | TTAGGCAT | GCCTACCT |
| RB339 | Lexington, KY | 38.014 | -84.504 | *P. echinata* | Yellow | GTCCGCAC | CCTTGCCATT |
| RB343_01 | Lexington, KY | 38.014 | -84.504 | *P. rigida* | Yellow | ATCACGAT | GGAACGA |
| RB355 | Lexington, KY | 38.014 | -84.504 | *P. virginiana* | Yellow | AGTCAACA | GGTGT |
| RB368 | Old Bridge, NJ | 40.36814 | -74.3022 | *P. rigida* | White | TGACCAAT | GGTGCACATT |
| RB369 | Old Bridge, NJ | 40.36814 | -74.3022 | *P. rigida* | White | TTAGGCAT | TGGCAACAGA |
| RB404 | Morehead, KY | 38.18613 | -83.5568 | *P. echinata* | Yellow | GTGAAACG | TCACTG |
