# Supplementary material for: The impact of life stage and pigment source on the evolution of novel warning signal traits
Source: Evolution. 2022 Feb 10;76(3):554–72. doi: 10.1111/evo.14443 (PMC9304160; doi:10.1111/evo.14443)
Supplement: Supplementary file 9 — Table S4 – Sequences for adapters containing variable‐length barcodes from Burford Reiskind et al. (2016). [file EVO-76-554-s012.docx]

**Table S4 – Sequences for adapters containing variable-length barcodes from Burford Reiskind *et al.* (2016).**

| **Barcode** | **P1.1 sequence** | **P1.2 sequence** |
| --- | --- | --- |
| ATTAT | ACACTCTTTCCCTACACGACGCTCTTCCGATCTATTATCATG | /5Phos/ATAATAGATCGGAAGAGCGTCGTGTAGGGAAAGAGTGT |
| CACCA | ACACTCTTTCCCTACACGACGCTCTTCCGATCTCACCACATG | /5Phos/TGGTGAGATCGGAAGAGCGTCGTGTAGGGAAAGAGTGT |
| CCTCG | ACACTCTTTCCCTACACGACGCTCTTCCGATCTCCTCGCATG | /5Phos/CGAGGAGATCGGAAGAGCGTCGTGTAGGGAAAGAGTGT |
| CTTGA | ACACTCTTTCCCTACACGACGCTCTTCCGATCTCTTGACATG | /5Phos/TCAAGAGATCGGAAGAGCGTCGTGTAGGGAAAGAGTGT |
| GGATA | ACACTCTTTCCCTACACGACGCTCTTCCGATCTGGATACATG | /5Phos/TATCCAGATCGGAAGAGCGTCGTGTAGGGAAAGAGTGT |
| GGTGT | ACACTCTTTCCCTACACGACGCTCTTCCGATCTGGTGTCATG | /5Phos/ACACCAGATCGGAAGAGCGTCGTGTAGGGAAAGAGTGT |
| TATGT | ACACTCTTTCCCTACACGACGCTCTTCCGATCTTATGTCATG | /5Phos/ACATAAGATCGGAAGAGCGTCGTGTAGGGAAAGAGTGT |
| TGCTT | ACACTCTTTCCCTACACGACGCTCTTCCGATCTTGCTTCATG | /5Phos/AAGCAAGATCGGAAGAGCGTCGTGTAGGGAAAGAGTGT |
| AACTGG | ACACTCTTTCCCTACACGACGCTCTTCCGATCTAACTGGCATG | /5Phos/CCAGTTAGATCGGAAGAGCGTCGTGTAGGGAAAGAGTGT |
| ACAACT | ACACTCTTTCCCTACACGACGCTCTTCCGATCTACAACTCATG | /5Phos/AGTTGTAGATCGGAAGAGCGTCGTGTAGGGAAAGAGTGT |
| ATAGAT | ACACTCTTTCCCTACACGACGCTCTTCCGATCTATAGATCATG | /5Phos/ATCTATAGATCGGAAGAGCGTCGTGTAGGGAAAGAGTGT |
| CAGATA | ACACTCTTTCCCTACACGACGCTCTTCCGATCTCAGATACATG | /5Phos/TATCTGAGATCGGAAGAGCGTCGTGTAGGGAAAGAGTGT |
| GAAGTG | ACACTCTTTCCCTACACGACGCTCTTCCGATCTGAAGTGCATG | /5Phos/CACTTCAGATCGGAAGAGCGTCGTGTAGGGAAAGAGTGT |
| GGCTTA | ACACTCTTTCCCTACACGACGCTCTTCCGATCTGGCTTACATG | /5Phos/TAAGCCAGATCGGAAGAGCGTCGTGTAGGGAAAGAGTGT |
| TCTTGG | ACACTCTTTCCCTACACGACGCTCTTCCGATCTTCTTGGCATG | /5Phos/CCAAGAAGATCGGAAGAGCGTCGTGTAGGGAAAGAGTGT |
| TCACTG | ACACTCTTTCCCTACACGACGCTCTTCCGATCTTCACTGCATG | /5Phos/CAGTGAAGATCGGAAGAGCGTCGTGTAGGGAAAGAGTGT |
| ACCAGGA | ACACTCTTTCCCTACACGACGCTCTTCCGATCTACCAGGACATG | /5Phos/TCCTGGTAGATCGGAAGAGCGTCGTGTAGGGAAAGAGTGT |
| CCACTCA | ACACTCTTTCCCTACACGACGCTCTTCCGATCTCCACTCACATG | /5Phos/TGAGTGGAGATCGGAAGAGCGTCGTGTAGGGAAAGAGTGT |
| CCGAACA | ACACTCTTTCCCTACACGACGCTCTTCCGATCTCCGAACACATG | /5Phos/TGTTCGGAGATCGGAAGAGCGTCGTGTAGGGAAAGAGTGT |
| CTAAGCA | ACACTCTTTCCCTACACGACGCTCTTCCGATCTCTAAGCACATG | /5Phos/TGCTTAGAGATCGGAAGAGCGTCGTGTAGGGAAAGAGTGT |
| CTCGCGG | ACACTCTTTCCCTACACGACGCTCTTCCGATCTCTCGCGGCATG | /5Phos/CCGCGAGAGATCGGAAGAGCGTCGTGTAGGGAAAGAGTGT |
| GCGTCCT | ACACTCTTTCCCTACACGACGCTCTTCCGATCTGCGTCCTCATG | /5Phos/AGGACGCAGATCGGAAGAGCGTCGTGTAGGGAAAGAGTGT |
| GGAACGA | ACACTCTTTCCCTACACGACGCTCTTCCGATCTGGAACGACATG | /5Phos/TCGTTCCAGATCGGAAGAGCGTCGTGTAGGGAAAGAGTGT |
| TAGCCAA | ACACTCTTTCCCTACACGACGCTCTTCCGATCTTAGCCAACATG | /5Phos/TTGGCTAAGATCGGAAGAGCGTCGTGTAGGGAAAGAGTGT |

| ACTGCGAT | ACACTCTTTCCCTACACGACGCTCTTCCGATCTACTGCGATCATG | /5Phos/ATCGCAGTAGATCGGAAGAGCGTCGTGTAGGGAAAGAGTGT |
| --- | --- | --- |
| ATGAGCAA | ACACTCTTTCCCTACACGACGCTCTTCCGATCTATGAGCAACATG | /5Phos/TTGCTCATAGATCGGAAGAGCGTCGTGTAGGGAAAGAGTGT |
| GCCTACCT | ACACTCTTTCCCTACACGACGCTCTTCCGATCTGCCTACCTCATG | /5Phos/AGGTAGGCAGATCGGAAGAGCGTCGTGTAGGGAAAGAGTGT |
| TAGCGGAT | ACACTCTTTCCCTACACGACGCTCTTCCGATCTTAGCGGATCATG | /5Phos/ATCCGCTAAGATCGGAAGAGCGTCGTGTAGGGAAAGAGTGT |
| TGACGCCA | ACACTCTTTCCCTACACGACGCTCTTCCGATCTTGACGCCACATG | /5Phos/TGGCGTCAAGATCGGAAGAGCGTCGTGTAGGGAAAGAGTGT |
| ACGGTACT | ACACTCTTTCCCTACACGACGCTCTTCCGATCTACGGTACTCATG | /5Phos/AGTACCGTAGATCGGAAGAGCGTCGTGTAGGGAAAGAGTGT |
| AAGACGCT | ACACTCTTTCCCTACACGACGCTCTTCCGATCTAAGACGCTCATG | /5Phos/AGCGTCTTAGATCGGAAGAGCGTCGTGTAGGGAAAGAGTGT |
| TCAGAGAT | ACACTCTTTCCCTACACGACGCTCTTCCGATCTTCAGAGATCATG | /5Phos/ATCTCTGAAGATCGGAAGAGCGTCGTGTAGGGAAAGAGTGT |
| ATATCGCCA | ACACTCTTTCCCTACACGACGCTCTTCCGATCTATATCGCCACATG | /5Phos/TGGCGATATAGATCGGAAGAGCGTCGTGTAGGGAAAGAGTGT |
| GAGCGACAT | ACACTCTTTCCCTACACGACGCTCTTCCGATCTGAGCGACATCATG | /5Phos/ATGTCGCTCAGATCGGAAGAGCGTCGTGTAGGGAAAGAGTGT |
| GCAAGCCAT | ACACTCTTTCCCTACACGACGCTCTTCCGATCTGCAAGCCATCATG | /5Phos/ATGGCTTGCAGATCGGAAGAGCGTCGTGTAGGGAAAGAGTGT |
| AACGTGCCT | ACACTCTTTCCCTACACGACGCTCTTCCGATCTAACGTGCCTCATG | /5Phos/AGGCACGTTAGATCGGAAGAGCGTCGTGTAGGGAAAGAGTGT |
| TATTCGCAT | ACACTCTTTCCCTACACGACGCTCTTCCGATCTTATTCGCATCATG | /5Phos/ATGCGAATAAGATCGGAAGAGCGTCGTGTAGGGAAAGAGTGT |
| TCACGGAAG | ACACTCTTTCCCTACACGACGCTCTTCCGATCTTCACGGAAGCATG | /5Phos/CTTCCGTGAAGATCGGAAGAGCGTCGTGTAGGGAAAGAGTGT |
| TGGCACAGA | ACACTCTTTCCCTACACGACGCTCTTCCGATCTTGGCACAGACATG | /5Phos/TCTGTGCCAAGATCGGAAGAGCGTCGTGTAGGGAAAGAGTGT |
| CTCTCGCAT | ACACTCTTTCCCTACACGACGCTCTTCCGATCTCTCTCGCATCATG | /5Phos/ATGCGAGAGAGATCGGAAGAGCGTCGTGTAGGGAAAGAGTGT |
| AACGCACATT | ACACTCTTTCCCTACACGACGCTCTTCCGATCTAACGCACATTCATG | /5Phos/AATGTGCGTTAGATCGGAAGAGCGTCGTGTAGGGAAAGAGTGT |
| CCTTGCCATT | ACACTCTTTCCCTACACGACGCTCTTCCGATCTCCTTGCCATTCATG | /5Phos/AATGGCAAGGAGATCGGAAGAGCGTCGTGTAGGGAAAGAGTGT |
| CGTCGCCACT | ACACTCTTTCCCTACACGACGCTCTTCCGATCTCGTCGCCACTCATG | /5Phos/AGTGGCGACGAGATCGGAAGAGCGTCGTGTAGGGAAAGAGTGT |
| CGTGGACAGT | ACACTCTTTCCCTACACGACGCTCTTCCGATCTCGTGGACAGTCATG | /5Phos/ACTGTCCACGAGATCGGAAGAGCGTCGTGTAGGGAAAGAGTGT |
| GGTGCACATT | ACACTCTTTCCCTACACGACGCTCTTCCGATCTGGTGCACATTCATG | /5Phos/AATGTGCACCAGATCGGAAGAGCGTCGTGTAGGGAAAGAGTGT |
| TGGCAACAGA | ACACTCTTTCCCTACACGACGCTCTTCCGATCTTGGCAACAGACATG | /5Phos/TCTGTTGCCAAGATCGGAAGAGCGTCGTGTAGGGAAAGAGTGT |
| ACAACCAACT | ACACTCTTTCCCTACACGACGCTCTTCCGATCTACAACCAACTCATG | /5Phos/AGTTGGTTGTAGATCGGAAGAGCGTCGTGTAGGGAAAGAGTGT |
| CAACCACACA | ACACTCTTTCCCTACACGACGCTCTTCCGATCTCAACCACACACATG | /5Phos/TGTGTGGTTGAGATCGGAAGAGCGTCGTGTAGGGAAAGAGTGT |
