# Supplementary material for: The impact of life stage and pigment source on the evolution of novel warning signal traits
Source: Evolution. 2022 Feb 10;76(3):554–72. doi: 10.1111/evo.14443 (PMC9304160; doi:10.1111/evo.14443)
Supplement: Supplementary file 10 — Table S5 – Sequences for PCR primers, including degenerate bases. [file EVO-76-554-s006.docx]

**Table S5 – Sequences for PCR primers, including degenerate bases.** PCR1 is the universal primer used for all reactions, and the PCR2 primers contain their respective Illumina index sequences in their “Name”. When ordering, degenerate bases should be hand-mixed to ensure equal base composition.

| **Name** | **Sequence** |
| --- | --- |
| PCR1 | AATGATACGGCGACCACCGAGATCTACACTCTTTCCCTACACGACG |
| PCR2_ATCACGAT | CAAGCAGAAGACGGCATACGAGATNNNNATCGTGATGTGACTGGAGTTCAGACGTGTGC |
| PCR2_CGATGTAT | CAAGCAGAAGACGGCATACGAGATNNNNATACATCGGTGACTGGAGTTCAGACGTGTGC |
| PCR2_TTAGGCAT | CAAGCAGAAGACGGCATACGAGATNNNNATGCCTAAGTGACTGGAGTTCAGACGTGTGC |
| PCR2_TGACCAAT | CAAGCAGAAGACGGCATACGAGATNNNNATTGGTCAGTGACTGGAGTTCAGACGTGTGC |
| PCR2_ACAGTGAT | CAAGCAGAAGACGGCATACGAGATNNNNATCACTGTGTGACTGGAGTTCAGACGTGTGC |
| PCR2_GGCTACAT | CAAGCAGAAGACGGCATACGAGATNNNNATGTAGCCGTGACTGGAGTTCAGACGTGTGC |
| PCR2_AGTCAACA | CAAGCAGAAGACGGCATACGAGATNNNNTGTTGACTGTGACTGGAGTTCAGACGTGTGC |
| PCR2_CCGTCCCG | CAAGCAGAAGACGGCATACGAGATNNNNCGGGACGGGTGACTGGAGTTCAGACGTGTGC |
| PCR2_GTCCGCAC | CAAGCAGAAGACGGCATACGAGATNNNNGTGCGGACGTGACTGGAGTTCAGACGTGTGC |
| PCR2_GTGAAACG | CAAGCAGAAGACGGCATACGAGATNNNNCGTTTCACGTGACTGGAGTTCAGACGTGTGC |
| PCR2_GTGGCCTT | CAAGCAGAAGACGGCATACGAGATNNNNAAGGCCACGTGACTGGAGTTCAGACGTGTGC |
| PCR2_GTTTCGGA | CAAGCAGAAGACGGCATACGAGATNNNNTCCGAAACGTGACTGGAGTTCAGACGTGTGC |
