# Supplementary material for: The impact of life stage and pigment source on the evolution of novel warning signal traits
Source: Evolution. 2022 Feb 10;76(3):554–72. doi: 10.1111/evo.14443 (PMC9304160; doi:10.1111/evo.14443)
Supplement: Supplementary file 11 — Table S6. Per‐individual sequence statistic summary. [file EVO-76-554-s008.docx]

**Table S6. Per-individual sequence statistic summary.** For each specimen, the raw read number (Raw Reads), reads retained after the quality filtering described in text (Total Retained Reads), alignments retained after all filtering (Total Retained Reads), RAD loci formed in STACKS (Loci), and average stack coverage (Coverage) are given.

| **ID** | **Raw Reads** | **Total Retained Reads** | **Total Retained Alignments** | **Loci** | **Coverage** |
| --- | --- | --- | --- | --- | --- |
| LL004_01† | 3392337 | 3386292 | 1761040 | 25507 | 64.06 |
| LL005_01† | 2859217 | 2854217 | 1464391 | 20533 | 64.35 |
| LL010 | 1117784 | 1100193 | 623894 | 16891 | 31.73 |
| LL011 | 3943010 | 3936177 | 1849571 | 19933 | 84.5 |
| LL013 | 2592174 | 2517447 | 1341972 | 19830 | 61.1 |
| LL014 | 435182 | 434545 | 254326 | 10432 | 18.55 |
| LL015_01 | 2618314 | 2614028 | 1396017 | 22615 | 55.43 |
| LL042 | 1421807 | 1419626 | 765752 | 17797 | 38.68 |
| LL058_2 | 1638435 | 1635814 | 1025323 | 18479 | 17.89 |
| LL062_1 | 595932 | 594914 | 340148 | 10776 | 27.02 |
| LL064 | 2945519 | 2940999 | 1510039 | 19475 | 70.49 |
| LL070_1 | 536460 | 535612 | 294934 | 10131 | 10.24 |
| LL073_2R | 643236 | 642248 | 376076 | 11690 | 28.41 |
| LL074_5 | 2121052 | 2117733 | 1183883 | 21263 | 50.43 |
| LL084 | 1846912 | 1831375 | 984369 | 20105 | 43.15 |
| LL084_02 | 863843 | 859414 | 495786 | 15526 | 28.08 |
| LL084_03 | 564718 | 517179 | 294847 | 12061 | 18.84 |
| LL102 | 1623457 | 1620440 | 858439 | 18455 | 42.5 |
| LL137 | 1968589 | 1963700 | 1075452 | 16318 | 62.14 |
| LL142_02 | 326837 | 326241 | 180720 | 7288 | 14.85 |
| LL144_01 | 1297137 | 1295107 | 693559 | 16667 | 37.48 |
| LL180_02 | 267395 | 261535 | 158550 | 6632 | 13.72 |
| LL181 | 1493072 | 1458422 | 816972 | 17815 | 41.27 |
| LL194_02 | 1514444 | 1496147 | 815993 | 17812 | 40.52 |
| LL195_02 | 820948 | 813519 | 470504 | 14792 | 27.1 |
| RB020Db | 1092934 | 1083572 | 597498 | 15989 | 10.81 |
| RB028_01† | 522648 | 521584 | 221167 | 9325 | 15.67 |
| RB076_01 | 1121727 | 1120034 | 627012 | 17471 | 11.39 |
| RB107_01 | 1798351 | 1795127 | 951747 | 18799 | 45.78 |
| RB108_01 | 1483440 | 1481488 | 827779 | 18240 | 41.63 |
| RB110.01† | 615925 | 614969 | 332854 | 14096 | 20.02 |
| RB112_01 | 3015398 | 3011804 | 1455108 | 19906 | 66.13 |
| RB118_01 | 575688 | 574851 | 331489 | 10959 | 25.13 |
| RB119_01 | 3868679 | 3861825 | 1983946 | 22529 | 80 |
| RB126_01 | 1383786 | 1381717 | 791438 | 17903 | 40.07 |
| RB164 | 3634191 | 3628300 | 1797745 | 20178 | 82.03 |
| RB165 | 1870434 | 1865677 | 990006 | 18662 | 49.7 |
| RB167_01 | 1651619 | 1637675 | 868075 | 19402 | 39 |
| RB190 | 2180567 | 2177645 | 1195582 | 19203 | 57.3 |
| RB220 | 1353032 | 1350680 | 732886 | 17897 | 13.35 |
| RB221 | 1222920 | 1206576 | 676172 | 16682 | 35.58 |
| RB222 | 1765068 | 1761901 | 967646 | 20150 | 42.29 |
| RB223 | 2127812 | 2124632 | 1159096 | 20750 | 49.96 |
| RB224 | 2800109 | 2795584 | 1496620 | 23166 | 59.4 |
| RB225 | 890344 | 888893 | 476005 | 15951 | 26.31 |
| RB226_old | 2485213 | 2480964 | 1343856 | 22042 | 53.28 |
| RB227 | 5164322 | 5080004 | 2375809 | 21155 | 102.69 |
| RB228_new | 1869412 | 1799410 | 976671 | 20162 | 42.2 |
| RB229 | 911537 | 910028 | 515997 | 15472 | 29.21 |
| RB286 | 1354068 | 1351948 | 743790 | 16664 | 39.64 |
| RB287 | 3286964 | 3281135 | 1534479 | 19970 | 71.14 |
| RB288_02 | 1155682 | 1105050 | 590747 | 16425 | 31.42 |
| RB289 | 3260204 | 3180540 | 1648054 | 21712 | 68.77 |
| RB290 | 1374546 | 1353348 | 735191 | 17261 | 37.34 |
| RB304 | 2561735 | 2556768 | 1316229 | 19326 | 60.5 |
| RB305 | 3122874 | 3117501 | 1575124 | 19520 | 73.85 |
| RB306 | 1550475 | 1547645 | 849834 | 15426 | 19.92 |
| RB307 | 2135060 | 2131715 | 1142060 | 19231 | 53.6 |
| RB308 | 940884 | 939613 | 503080 | 16381 | 27.44 |
| RB339 | 2050440 | 2048052 | 1123669 | 18265 | 55.56 |
| RB343_01 | 2370469 | 2366356 | 1295458 | 21520 | 53.99 |
| RB355 | 5875532 | 5779433 | 2694341 | 22300 | 108.88 |
| RB368 | 2232246 | 2228235 | 1185784 | 19374 | 55.61 |
| RB369 | 2308643 | 2304329 | 1209457 | 19328 | 57.37 |
| RB404 | 334322 | 287694 | 172330 | 6282 | 16.98 |
