# Supplementary material for: The impact of life stage and pigment source on the evolution of novel warning signal traits
Source: Evolution. 2022 Feb 10;76(3):554–72. doi: 10.1111/evo.14443 (PMC9304160; doi:10.1111/evo.14443)
Supplement: Supplementary file 12 — Table S7. Random and fixed effects from the linear mixed model on Neodiprion lecontei and host plant (P. virginiana [VA], P. echinata [SL], and P. rigida) color traits. [file EVO-76-554-s001.docx]

**Table S7. Random and fixed effects from the linear mixed model on *N. lecontei* and host plant (*P. virginiana* [VA], *P. echinata* [SL]*,* and *P. rigida*) color traits.**

| *Color contrasts*  Random effects |  | |  | |  | |  | |  | |  |  |  |
| --- | --- | --- | --- | --- | --- | --- | --- | --- | --- | --- | --- | --- | --- |
| **Source of variation** | **Name** | | **σ^2^** | |  | | **SD** | |  | |  |  |  |
| Individual ID | Intercept | | 4.038 | |  | | 4.135 | |  | |  |  |  |
|  |  | |  | |  | |  | |  | |  |  |  |
| Fixed effects |  | |  | |  | |  | |  | |  |  |  |
| **Source of variation** | **Estimate** | | **s.e.** | | **DF** | | t | | **P** | |  |  |  |
| Intercept | 21.919 | | 1.607 | | 152 | | 13.644 | | <0.001* | |  |  |  |
| Larval color | -1.210 | | 2.272 | | 18 | | -0.533 | | 0.601 | |  |  |  |
| Host plant SL | 1.902 | | 1.068 | | 152 | | 1.782 | | 0.077 | |  |  |  |
| Host plant VA | 0.884 | | 1.068 | | 152 | | 0.828 | | 0.409 | |  |  |  |
| Plant part Old foliage | 5.122 | | 1.068 | | 152 | | 4.797 | | <0.001* | |  |  |  |
| Plant part Young foliage | 4.728 | | 1.068 | | 152 | | 4.428 | | <0.001* | |  |  |  |
| Larval color Y * Host plant SL | -0.139 | | 1.510 | | 152 | | -0.092 | | 0.927 | |  |  |  |
| Larval color Y * Host plant VA | -0.214 | | 1.510 | | 152 | | -0.142 | | 0.888 | |  |  |  |
| Larval color Y * Plant part Old foliage | -0.616 | | 1.510 | | 152 | | -0.408 | | 0.684 | |  |  |  |
| Larval color Y * Plant part young foliage | -0.840 | | 1.510 | | 152 | | -0.556 | | 0.579 | |  |  |  |
| *Luminance contrasts*  Random effects | |  | |  | |  | |  | |  | | |  |
| **Source of variation** | | **Name** | | **σ^2^** | |  | | **SD** | |  | | |  |
| Individual ID | | Intercept | | 5.167 | |  | | 3.227 | |  | | |  |
|  | |  | |  | |  | |  | |  | | |  |
| Fixed effects | |  | |  | |  | |  | |  | | |  |
| **Source of variation** | | **Estimate** | | **s.e.** | | **DF** | | t | | **P** | | |  |
| Intercept | | 16.729 | | 1.802 | | 152 | | 9.285 | | <0.001* | | |  |
| Larval color | | 3.240 | | 2.548 | | 18 | | 1.271 | | 0.220 | | |  |
| Host plant SL | | 12.953 | | 0.834 | | 152 | | 15.537 | | <0.001* | | |  |
| Host plant VA | | 11.994 | | 0.833 | | 152 | | 14.396 | | <0.001* | | |  |
| Plant part Old foliage | | -0.540 | | 0.837 | | 152 | | -0.645 | | <0.520 | | |  |
| Plant part Young foliage | | -11.665 | | 0.827 | | 152 | | -14.101 | | <0.001* | | |  |
| Larval color Y * Host plant SL | | 0.713 | | 1.179 | | 152 | | 0.605 | | 0.546 | | |  |
| Larval color Y * Host plant VA | | 0.612 | | 1.178 | | 152 | | 0.519 | | 0.605 | | |  |
| Larval color Y * Plant part Old foliage | | 0.325 | | 1.181 | | 152 | | 0.275 | | 0.783 | | |  |
| Larval color Y * Plant part young foliage | | -0.369 | | 1.174 | | 152 | | -0.315 | | 0.754 | | |  |
